# The Use of High-Throughput DNA Sequencing in the Investigation of Antigenic Variation: Application to *Neisseria* Species

**DOI:** 10.1371/journal.pone.0086704

**Published:** 2014-01-22

**Authors:** John K. Davies, Paul F. Harrison, Ya-Hsun Lin, Stephanie Bartley, Chen Ai Khoo, Torsten Seemann, Catherine S. Ryan, Charlene M. Kahler, Stuart A. Hill

**Affiliations:** 1 Department of Microbiology, Monash University, Clayton, Victoria, Australia; 2 Victorian Bioinformatics Consortium, Monash University, Clayton, Victoria, Australia; 3 School of Pathology and Laboratory Medicine; 4 The Marshall Centre for Infectious Diseases, Research and Training, University of Western Australia, Nedlands, Western Australia, Australia; 5 Telethon Institute of Child Health Research, University of Western Australia, Nedlands, Western Australia, Australia; 6 Department of Biological Sciences, Northern Illinois University, DeKalb, Illinois, United States of America; University of Würzburg, Germany

## Abstract

Antigenic variation occurs in a broad range of species. This process resembles gene conversion in that variant DNA is unidirectionally transferred from partial gene copies (or silent loci) into an expression locus. Previous studies of antigenic variation have involved the amplification and sequencing of individual genes from hundreds of colonies. Using the *pilE* gene from *Neisseria gonorrhoeae* we have demonstrated that it is possible to use PCR amplification, followed by high-throughput DNA sequencing and a novel assembly process, to detect individual antigenic variation events. The ability to detect these events was much greater than has previously been possible. In *N. gonorrhoeae* most silent loci contain multiple partial gene copies. Here we show that there is a bias towards using the copy at the 3′ end of the silent loci (copy 1) as the donor sequence. The *pilE* gene of *N. gonorrhoeae* and some strains of *Neisseria meningitidis* encode class I pilin, but strains of *N. meningitidis* from clonal complexes 8 and 11 encode a class II pilin. We have confirmed that the class II pili of meningococcal strain FAM18 (clonal complex 11) are non-variable, and this is also true for the class II pili of strain NMB from clonal complex 8. In addition when a gene encoding class I pilin was moved into the meningococcal strain NMB background there was no evidence of antigenic variation. Finally we investigated several members of the *opa* gene family of *N. gonorrhoeae,* where it has been suggested that limited variation occurs. Variation was detected in the *opaK* gene that is located close to *pilE*, but not at the *opaJ* gene located elsewhere on the genome. The approach described here promises to dramatically improve studies of the extent and nature of antigenic variation systems in a variety of species.

## Introduction

Antigenic variation is a genetic process that leads to high-frequency changes in cell surface components in a wide range of species. To evaluate the experimental approach described here we initially investigated antigenic variation in a gene where that process has been well studied, the *pilE* gene of *Neisseria gonorrhoeae*. *N. gonorrhoeae* (the gonococcus), and the closely related *N. meningitidis* (the meningococcus), are both strict human pathogens and must continually evade the human immune system. What partly confounds the immune system is antigenic variation of the PilE pilin subunit that assembles into the type IV pilus. In this system the expressed gene (*pilE*) changes and the so-called silent loci (*pilS*) donate variant genetic information, but remain unchanged in the process. The variant sequences recombine into the distal two thirds of the *pilE* gene [1], and the process is dependent on the presence of the RecA protein [2]. Mosaic proteins can sometimes be formed as multiple gene segments from the *pilS* loci are recombined into the expressed copy to yield a variant gene [1]. Antigenic variation in this gene is a high-frequency event, with approximately 12% of randomly selected colonies containing a variant gene sequence [1]. Two distinct classes of type IV pili are found in *N. meningitidis*: class I pili (also found in *N. gonorrhoeae*) antigenically vary, whereas at least some class II pili do not [3].

In addition to the type IV pilin system, other potential antigenic variation systems have been identified in the genome sequences of *Neisseria* species. The *opa* genes are a gene family encoding surface-exposed proteins, with the number of genes varying between strains. For *N. gonorrhoeae* strain MS11, nine intact *opa* genes have been cloned and sequenced allowing alignment of their gene sequences [4]. From these alignments two hyper-variable regions can be observed in the middle of the genes as well as a semi-variable region located towards the 5′ end. Several studies have shown DNA transformation-mediated horizontal transmission of chromosomal DNA where hyper-variable *opa* segments are exchanged between strains [5]–[7]. However, limited data has been accrued as to whether *opa* genes also engage in antigenic variation as observed with the *pil* system. Anecdotal evidence suggests that limited variation does occur in at least some *opa* genes [4].

Various methods have been used to determine the extent and nature of antigenic variation within a population [1], [8]. Recent studies have involved the amplification of the *pilE* gene from hundreds of randomly selected colonies, followed by sequencing of each amplicon using Sanger sequencing technology to detect the subset that contained variant sequences [1], [3], [9]. Because of the cost and time involved the number of genes that were eventually sequenced was limiting, leading to conclusions being based on small data sets. For example, the extent of *pilE* gene variation in *N. gonorrhoeae* was based on analysis of 497 amplicons, and that for *N. meningitidis* on 260 amplicons [1], [3]. We reasoned that high throughput sequencing of DNA extracted from a varying culture could be used to detect multiple gene variants simultaneously, especially those that might be occurring with a low frequency. The more cells in the culture that are producing a variant sequence, the more variant DNA will be found in the DNA preparation. Comparing the sequence depth of a variant sequence with the sequence depth of all DNA in the sample would therefore enable an estimation of the frequency at which variant cells were present in the culture. This approach has dramatically increased the size of the data sets allowing a more detailed picture to emerge of the extent and nature of antigenic variation.

## Materials and Methods

### Bacterial Strains and Growth Conditions

Initial experiments used gonococcal strains FA1090 and MS11 containing the *recA6* allele [10]. This allele was first introduced into strain MS11, and subsequently genomic DNA from MS11 *recA6* was used to transform strain FA1090. In these strains the *recA* gene is transcribed from a promoter that is only operational in the presence of the inducer isopropyl thiogalactopyranoside (IPTG). Growing the strain in the absence of IPTG halts RecA production, and effectively “freezes” antigenic variation. The *N. meningitidis* strains used were NMB and FAM18, both of which produce class II pilin. The details of the construction of *N. meningitidis* strain CKNM397 have been described elsewhere [11]. Briefly CKNM397 is *N. meningitidis* strain NMB producing the class I pilin from *N. meningitidis* strain MC58. The *pilE* gene and upstream sequence containing the native promoter and a G4 sequence important for antigenic variation [12], was PCR amplified from strain MC58 and integrated into the *iga* gene of strain NMB. Subsequently the NMB *pilE* gene encoding class II pilin was insertionally inactivated. The solid and liquid media, and the growth conditions used have been described previously [13].

### Amplification and Sequencing of Genes of Interest

A single colony of the relevant strain was picked from a GC agar plate and resuspended in 60–100 µl of GC broth. Neither the agar plate nor the broth contained IPTG, so in those strains containing the IPTG-inducible *recA6* allele the *recA* gene was not being expressed, and antigenic variation was absent. A portion of this suspension was plated onto GC agar and incubated overnight. Strains that contained the *recA6* allele were plated onto both GC agar and GC agar containing 2 mM IPTG. The remainder of the original suspension was retained and used to generate a reference sequence by Sanger sequencing. The cells were harvested from the agar plates, and genomic DNA was extracted from both these samples and the reference sample using the GenElute™ Bacterial Genomic DNA Kit (Sigma). The genomic DNA was used as template in PCR with KOD Hot Start DNA Polymerase (Merck). The oligonucleotide primers used to PCR amplify the genes of interest are listed in Table S1 in the Supporting Information. These were designed such that they bound approximately 300 bp upstream and downstream of the gene of interest. The PCR conditions were as follows: 95°C for 2 mins followed by 35 cycles of 95°C for 20 seconds, 55°C for 10 seconds and 70°C for 20 seconds. The PCR products were then gel-purified with QIAquick Gel Extraction Kit (QIAGEN). The PCR product from the reference sample was then subjected to Sanger sequencing using the same primers that were used for amplification.

The amplicons were prepared for high-throughput sequencing using the Illumina Genomic DNA Sample Prep Kit as per the manufacturer’s instructions. Sequence data were obtained from an Illumina Genome Analyzer II using 36-cycle (Illumina 36C Sequencing Kit V2), or 72-cycle when it became available, paired-end chemistry. The raw sequence data from each sample will be deposited in the NCBI Sequence Read Archive, and the relevant sequence quality data are shown in Table S2 of the Supporting Information.

### Assembly of Sequence Reads

Existing assembly programs, designed to handle moderate coverage of whole genome sequences, proved inadequate when faced with the extensive coverage of relatively short amplicon sequences used here. A novel assembly procedure was therefore developed to deal with the unusual nature of the high-throughput sequencing data produced. The procedure has three stages, an assembly stage to produce a large number of candidate sequences (500 per sample, or per RecA^−^/RecA^+^ pair of samples), a selection stage to pick a smaller number of sequences that explain as much of the read data as possible (50 per sample or per RecA^−^/RecA^+^ pair of samples), and finally a manual validation stage to identify and remove any mis-assemblies.

The assembly procedure is a variant on k-mer based assembly [14]. A k-mer is a string of *k* contiguous nucleotides, and a sequence of nucleotides of length *n* can be viewed as a sequence of *n*−*k*+1 overlapping k-mers. K-mer based assembly is usually based on statistics on the number of times each k-mer occurs in the read set. We added to this statistics on the number of k-mer pairs. All ordered k-mer pairs observed in the read pairs were counted. That is, each pair of k-mers occurring one after the other in a read, or one k-mer in the first read and one k-mer in the paired read. K-mer pair counts were used both in the assembly stage, as part of a seed-and-extend algorithm, and in the validation stage, where visualization of k-mer pair counts of an assembled sequence allowed manual validation of the correctness of assembled sequences. The details are discussed in a supplementary text file (Text File S1) in the Supporting Information, and the software is available for download at http://www.vicbioinformatics.com/software.assemblet.shtml.

### Nomenclature of *pilS* Silent Loci

We have continued the use of the system adopted by others to name the individual partial gene sequences located in the various silent loci [1], [3], [9]. Gonococcal strain FA1090 contains 19 partial gene copies [15]. All but one of these is found in five *pilS* loci distributed around the genome. Here for instance *pilS1c3* refers to the third partial gene copy in the silent locus *pilS1*. One additional partial gene copy is found just upstream of the *pilE* gene, and has been designated *pilEc2* [15]. Gonococcal strain MS11 also has 5 *pilS* loci, containing 13 partial gene copies [16]. An additional 2 partial gene copies are found upstream of the *pilE* gene. In some variants of this strain the *pilE* gene and upstream copies are duplicated. Only 11 of these 17 possible partial gene copies have been sequenced [16]. A summary of the *pilS* loci and the partial gene copies they contain, along with the relevant accession numbers, is shown in Table 1.

**Table 1:** Partial *pilE* gene copies found within the *pilS* loci of *N. gonorrhoeae*.

Strain	*pilS* locus	Partial genecopies	Accession number
FA1090	*pilS1*	5	U58846
	*pilS2*	6	U58848
	*pilS3*	3	U58850
	*pilS6*	3	U58849
	*pilS7*	1	U58851
	*pilEc2*	1	U58847
MS11	*pilS1*	6	M11663
	*pilS2*	2	None
	*pilS5*	1	X60748
	*pilS6*	3	X60749
	*pilS7*	1	X60750
	Upstream of *pilE*	2	None

## Results

### Analysis of Sequence Assemblies

Each of the 50 sequence assemblies emerging from the selection stage was used as a query sequence in a BLASTn search of the databases, and any assembly that did not relate to the gene and strain in question was removed from further consideration. These were mainly short assemblies with very low sequence coverage. The remaining assemblies were aligned to the reference sequence using ClustalW2. This, along with heat maps (see Text File S1 in Supplementary Information), occasionally identified additional mis-assemblies arising from the short Illumina sequence reads, and the presence of direct or inverted repeats in the amplified region. These were also removed from the analysis. The remaining assemblies were then manually searched for those that differed from the reference sequence by just one nucleotide, such as a one nucleotide insertion or deletion, or a nucleotide change. For the reasons outlined below, these assemblies were also removed from further analysis. As expected, prominent amongst the remaining assemblies was an assembly (allele 1) with high k-mer depth that was identical to the reference sequence obtained by Sanger sequencing of the amplicon. The remaining assemblies were again aligned with the reference sequence using ClustalW2. In most cases the average k-mer depth exceeded 10^5^, and in some cases 10^6^ (Table 2). The frequency with which a particular variant appeared was estimated by dividing the average k-mer depth of the variant portion of the assembly by the average k-mer depth of same-sized regions immediately on either side of the variant sequence. This is illustrated for a particular assembly containing a variant sequence in Figure S1, Panel A. In some cases regions containing variant sequences were interrupted by region(s) of conserved sequence. An example of this is shown in Figure S1, Panel B. In these cases the spike in k-mer depth resulting from the presence of the internal conserved region would inflate the average k-mer depth if this was measured across the entire region, resulting in an artificially high frequency calculation. Therefore for these assemblies the frequency was calculated by dividing the k-mer depth of each individual variable segment by the average k-mer depth of same-sized regions immediately on either side of the entire region.

**Table 2:** Average k-mer depth of allele 1, the sequence assembly that was identical to the reference sequence, for each experiment.

Strain	Gene	Experiment	Average k-mer depthRecA^−^ (×10^3^)	Average k-mer depthRecA^+^ (×10^3^)
*N. gonorrhoeae* FA1090	*pilE*	1	250	1070
*N. gonorrhoeae* FA1090	*pilE*	2	248	158
*N. gonorrhoeae* MS11	*pilE*	1	240	202
*N. gonorrhoeae* MS11	*pilE*	2	264	278
*N. meningitidis* FAM18	*pilE*	1	–	1540
*N. meningitidis* FAM18	*pilE*	2	–	1062
*N. meningitidis* NMB	*pilE*	1	–	669
*N. meningitidis* CKNM397	*pilE*	1	–	300
*N. meningitidis* CKNM397	*pilE*	2	–	220
*N. gonorrhoeae* FA1090	*opaK*	1	159	208
*N. gonorrhoeae* FA1090	*opaK*	2	87	110

### Variation at the *pilE* Gene Encoding Class I Pilin in *N. gonorrhoeae* Strain FA1090

The *pilE* of strain FA1090 *recA6* grown in the absence of IPTG was amplified by PCR and sequenced using Sanger chemistry. An alignment with the FA1090 genome sequence (accession number AE004969) revealed sequence variation in the 3′ part of the gene (Figure S2). This variation can be explained by gene conversion using sequence from a specific silent locus, *pilS1c1*. In two independent experiments a culture grown in the absence of IPTG was split, with one half cultured in the absence of IPTG, whilst the other half was grown in the presence of IPTG, allowing antigenic variation. The *pilE* gene from both cultures was amplified by PCR, and subjected to high-throughput sequencing.

In both experiments there were multiple assemblies that differed from the reference sequence by just one nucleotide. Unlike the variant sequences described below, the single nucleotide changes were not concentrated in the 3′ end of the *pilE* gene, but were scattered throughout the *pilE* gene and the flanking sequences. In both experiments, only one assembly involved a single nucleotide change that occurred at higher frequency in the presence of RecA. The same change was detected in both experiments, and involved a single nucleotide change downstream of the *pilE* gene. It therefore seems that almost all of the assemblies involving single nucleotide changes were not the result of antigenic variation. Others have reported that antigenic variation can result in single nucleotide changes [1], but in our hands it seems more likely that these are the result of low frequency mutations occurring during amplification of the gene or (less likely) sequencing errors. Such changes were therefore not considered further.

In the first experiment 29 assemblies passed the assembly and screening process described above, while in the second experiment (maybe because of a much lower average k-mer depth in the RecA^+^ sample; Table 2) just six assemblies were detected. The results are shown in Table 3 and Table 4, for the first and second experiments, respectively. All of the variants were present at basal levels in the absence of RecA, and at a much higher frequency in the presence of RecA, suggesting active gene conversion during the experiment. In the absence of RecA the average kmer depth across the variant portion of each assembly was less than 10, compared with 10^5^–10^6^ for the rest of the assembly. In each case the kmer depth fell to zero for a portion of that variant sequence. An example of this can be seen in Figure S1, panel A. We used the average kmer depth across the entire variant sequence to calculate the frequency, rather than the minimal kmer depth (zero) for just a portion of the variant sequence. In agreement with the suggestion that this represents antigenic variation, alignments of the assembled sequences are shown in Figures S3 and S4 and demonstrate that sequence variation was confined to the 3′ end of the *pilE* gene.

**Table 3:** Variant sequences detected in the first experiment with *pilE* from *N. gonorrhoeae* FA1090.

Assembly	Donorsequence	5′ sequenceidentity (nt)	Variantsequence (nt)	3′ sequenceidentity (nt)	RecA^−^frequency(×10^−3^)^a^	RecA^+^ frequency(×10^−3^)^a^	RecA^+^/RecA^−^ ratio^b^
Allele 1	Reference	–	–	–	–	–	–
Allele 2	*pilS3c1*	23	169	39	0.062	10.4	168
Allele 3	*pilS6c1*	59	82	141	0.021	7.19	342
Allele 4	*pilS2c4*	42	87	39	0.070	6.42	92
Allele 5	*pilS3c3*	4	192	40	0.104	3.78	36
Allele 6	*pilS3c1*	39	10	234	0.027	15.1	559
Allele 7	*pilS1c2*	19	84	36	0.088	4.10	47
Allele 8	*pilS7c1*	35	125	106	0.018	2.68	149
Allele 9	*pilS7c1* or	68	5	19	0.019	5.91	311
	*pilS6c2*	111	5	44			
Allele 10	*pilS6c1*	57	178	59	0.038	7.50	197
Allele 11	*pilS6c2*	40	65	47	0.013	2.86	220
Allele 12	*pilS1c1*	8	71	228	0.034	5.96	175
Allele 13	*pilS6c2*	44	12	47	0.027	4.66	173
Allele 14	*pilS2c1*	3	96	141	0.023	4.19	182
Allele 15	*pilS1c5*	55	192	41	0.057	2.36	41
Allele 16	*pilS3c3*	56	137	23	0.104	3.48	34
Allele 17	*pilS1c4*	59	51	30	0.011	3.67	334
Allele 18	*pilS1c4*	5	6	18	0.075	0.92	12
Allele 19	*pilS3c3*	39	52	29	0.010	2.31	231
Allele 20	*pilS7c1*	56	71	68	0.024	1.78	74
Allele 21	*pilS2c4*	43	43	48	0.009	0.60	67
Allele 22	*pilS7c1*	68	62	35	0.015	4.76	317
Allele 23	*pilS1c1*	11	89	228	0.043	5.25	122
Allele 24	*pilS1c5*	6	13	18	0.003	0.81	270
Allele 25	mosaic	–	117	–	0.014	3.23	231
Allele 26	*pilS3c3*	5	38	26	0.019	3.02	159
Allele 27	*pilEc2*	47	4	42	0.031	1.22	39
Allele 28	*pilS2c1* or	15	34	4	0.043	5.50	128
	*pilS3c1*	11	34	32			
Allele 29	*pilS1c2*	36	40	55	0.011	4.97	452

a The average k-mer depth of the variant portion of the assembly divided by the average k-mer depth of same-sized regions immediately on either side of the variant sequence.

b The frequency in the presence of RecA divided by the frequency in the absence of RecA.

**Table 4:** Variant sequences detected in the repeat experiment with *pilE* from *N. gonorrhoeae* FA1090.

Assembly	Donorsequence	5′ sequenceidentity (nt)	Variantsequence (nt)	3′ sequenceidentity (nt)	RecA^−^ frequency(×10^−3^)^a^	RecA^+^ frequency(×10^−3^)^a^	RecA^+^/RecA^−^ratio^b^
Allele 1	Reference	–	–	–	–	–	–
Allele 3	*pilS6c1*	59	82	141	0.027	6.11	226
Allele 4	*pilS2c4*	42	87	85	0.10	12.8	128
Allele 30	*pilS6c2*	111	165	48	0.075	3.91	52.1
Allele 31	*pilS1c2*	32	176	14	0.018	3.43	191
Allele 32	*pilS7c1*	68	263	106	0.244	1.60	6.56

a The average k-mer depth of the variant portion of the assembly divided by the average k-mer depth of same-sized regions immediately on either side of the variant sequence.

b The frequency in the presence of RecA divided by the frequency in the absence of RecA.

Of the 28 variant sequences detected in the first experiment, 27 were identical to a *pilS* locus in the FA1090 genome sequence (Table 3). In the remaining case (allele 25) a mosaic sequence was present, derived from multiple silent loci. This is possibly derived from two separate recombination events involving *pilS2c3* and *pilS2c4*. In two cases the variant sequence was identical to more than one silent locus, so it was not possible to unambiguously map the donor sequence. It was also evident that a particular silent locus can be involved in generating multiple variants. For instance, different portions of *pilS3c3* were involved in generating alleles 4, 16, 19 and 26 (Table 3, Figure S3). In the repeat experiment all five variant sequences were identical to part of a silent locus (Table 4). Two variant sequences (alleles 3 and 4) appeared in both experiments. A variety of silent loci served as the source of donor sequences, with 12 of the 19 silent copies potentially involved.

### Variation at the *pilE* Gene Encoding Class I Pilin in *N. gonorrhoeae* Strain MS11

The *pilE* gene of gonococcal strain MS11 is also antigenically variable, but reportedly at a lower frequency than in strain FA1090 [9]. In order to determine whether the approach outlined above could detect such differences, we again conducted two separate experiments, using strain MS11*recA6*. In the first experiment 15 assemblies passed the screening procedure (Table 5, Figure S5), while the second experiment yielded 8 assemblies (Table 6, Figure S6). In both experiments allele 1 was identical to both the reference sequence, and the MS11 *pilE* sequence deposited in the databases (Accession number K02078). In agreement with the earlier report [9] the frequency of antigenic variation observed was lower than that seen in strain FA1090 (Table 5 and Table 6). Rather than being barely detectable in the RecA^-^ culture, the variant sequences were present at a higher frequency than seen in FA1090, suggesting that despite single colony isolations a variant subpopulation was present. This occurred in both experiments and might suggest that the *recA* promoter is not as tightly controlled in the MS11 genetic background. As a result the presence of RecA only boosted the variant frequency approximately 10-fold in MS11, compared with approximately 100-fold in FA1090 (Table 3, Table 4). Not all silent loci in strain MS11 have been sequenced and annotated. As a result in three cases in both experiments, it was not possible to assign a specific silent locus as the source of the donor sequence. Alleles 9 and 11 appeared in both experiments.

**Table 5:** Variant sequences detected in the first experiment with *pilE* from *N. gonorrhoeae* MS11.

Assembly	Donorsequence	5′ sequenceidentity (nt)	Variantsequence (nt)	3′ sequenceidentity (nt)	RecA^−^ frequency(×10^−3^)^a^	RecA^+^ frequency(×10^−3^)^a^	RecA^+^/RecA^−^ratio^b^
Allele 1	Reference	–	–	–	–	–	–
Allele 2	*pilS1c2*	50	197	41	0.40	2.26	5.65
Allele 3	Uncertain^c^	–	166	–	0.45	2.56	5.69
Allele 4	*pilS5c1*	38	163	51	0.34	1.70	5.00
Allele 5	*pilS1c1*	34	50	102	0.22	1.73	7.86
Allele 6	*pilS1c4*	32	95	23	0.27	1.12	4.15
Allele 7	*pilS7c1*	34	12	102	1.22	5.48	4.49
Allele 8	*pilS1c1 or*	41	64	34	0.70	1.25	1.79
	*pilS1c3*	41	64	34			
Allele 9	Uncertain^c^	–	165	–	0.16	1.45	9.06
Allele 10	*pilS7c1*	45	47	37	0.32	1.68	5.25
Allele 11	*pilS6c1*	53	48	37	0.17	1.52	8.94
Allele 12	*pilS1c4*	59	44	30	0.21	1.01	4.81
Allele 13	*pilS5c1*	50	56	32	0.19	1.03	5.42
Allele 14	*pilS1c2*	41	64	40	0.21	0.82	3.90
Allele 15	Uncertain^c^	–	68	–	0.28	0.43	1.54

a The average k-mer depth of the variant portion of the assembly divided by the average k-mer depth of same-sized regions immediately on either side of the variant sequence.

b The frequency in the presence of RecA divided by the frequency in the absence of RecA.

c Not all silent loci in strain MS11 have been sequenced and annotated. The donor sequence could be one of these “missing” silent copies. Alternatively this allele may represent a mosaic sequence derived from multiple recombination events.

**Table 6:** Variant sequences detected in the repeat experiment with *pilE* from *N. gonorrhoeae* MS11.

Assembly	Donor sequence	5′ sequenceidentity (nt)	Variantsequence (nt)	3′ sequenceidentity (nt)	RecA^−^ frequency(×10^−3^)^a^	RecA^+^ frequency(×10^−3^)^a^	RecA^+^/RecA^−^ratio^b^
Allele 1	Reference	–	–	–	–	–	–
Allele 16	*pilS5c1*	7	155	51	0.44	1.96	4.45
Allele 17	Uncertain^c^	–	132	–	0.55	2.81	5.11
Allele 18	Uncertain^c^	–	204	–	0.42	2.23	5.31
Allele 9	Uncertain^c^	–	126	–	0.18	1.63	9.06
Allele 11	*pilS6c1*	53	48	37	0.12	1.23	10.3
Allele 19	*pilS7c1*	82	12	55	0.16	1.90	11.9
Allele 20	*pilS6c1*	12	53	54	0.022	0.61	27.7

a The average k-mer depth of the variant portion of the assembly divided by the average k-mer depth of same-sized regions immediately on either side of the variant sequence.

b The frequency in the presence of RecA divided by the frequency in the absence of RecA.

c Not all silent loci in strain MS11 have been sequenced and annotated. The donor sequence could be one of these “missing” silent copies. Alternatively this allele may represent a mosaic sequence derived from multiple recombination events.

### Lack of Variation in the *pilE* Gene of Strains of *N. meningitidis* that Express Class II Pilin

Meningococci also express type IV pili, with at least some of the class I pilin subunits also varying antigenically [17]. However the class II pili of *N. meningitidis* FAM18 appear not to vary [3]. We therefore searched for evidence of *pilE* variation in FAM18 and another meningococcal strain, NMB, which also produces class II pilin. In neither strain were we able to detect antigenic variation despite adequate k-mer depth (Table 2). We also looked for antigenic variation in meningococcal strain CKNM397. This strain is derived from strain NMB but produces the class I pilin from strain MC58 instead of the native class II pilin [11]. In two separate experiments we were unable to detect any antigenic variants despite adequate k-mer depth (Table 2).

### Variation at *opa* Genes

For the reasons outlined above, we also investigated whether there was any evidence of antigenic variation in the *opa* genes of *N. gonorrhoeae* strain FA1090. In an initial experiment we were unable to detect any variation in the FA1090 *opaJ* gene (NGO1922) (data not shown). However it has been reported that the *opaK* gene, situated close to the *pilE* gene, is more variable than other *opa* loci [7]. In two separate experiments, we were able to detect variant sequences in *opaK* (NGO2132) (Table 7, Figure S7 and Figure S8). In each case the variant portion of the assembly was identical to part of the *opaD* gene (NGO1513), which appeared to act as the donor sequence in these experiments. However in the first experiment the variant sequences are clearly present in the RecA^-^ sample and the presence of RecA boosts their levels only marginally (Table 7). In the second experiment both variants display a probably unrelated change in a CTCTT-repeat region known to be subject to slipped-strand mis-pairing. Other than this change allele 2 in the first experiment is identical to allele 5 in the second experiment. In this second experiment both variants appeared much more frequently in the presence of RecA (Table 7).

**Table 7:** Variation in the *opaK* gene of *N. gonorrhoeae* strain FA1090.

Experiment	Assembly	RecA^−^ frequency (×10^−3^)^a^	RecA^+^ frequency (×10^−3^)^a^	RecA^+^/RecA^−^ ratio^b^
1	Allele 1^c^	–	–	–
1	Allele 2	6.21	10.24	1.65
1	Allele 3	3.94	6.50	1.65
1	Allele 4	4.06	6.56	1.62
2	Allele 1^c^	–	–	–
2	Allele 5	0.35	9.13	26.16
2	Allele 6	1.05	5.85	5.57

a The average k-mer depth of the variant portion of the assembly divided by the average k-mer depth of same-sized regions immediately on either side of the variant sequence.

b The frequency in the presence of RecA divided by the frequency in the absence of RecA.

c Allele 1 was identical to the reference sequence.

## Discussion

Previous investigations of the extent and nature of antigenic variation in *Neisseria* have involved the amplification of genes from hundreds of individual colonies that were then sequenced by conventional Sanger sequencing technology in order to detect the subset that contained variant sequences [1], [3], [8]. Such an approach was both time-consuming and expensive. The advent of affordable deep sequencing platforms has enabled an alternative approach to such studies. Here we have used PCR amplification of the genes of interest, followed by high throughput DNA sequencing, to detect variant sequences. This involved a single PCR amplification rather than hundreds, one sequencing reaction rather than hundreds, and an improved ability to detect low-frequency variants. The data shown in Table 3 and Table 4 suggest that this approach is indeed capable of detecting examples of antigenic variation, in that the variant sequences detected in these experiments are typical of antigenic variation events. They are (a) restricted to the 3′ end of the *pilE* gene, (b) detected at a high frequency only in the presence of RecA, and (c) in almost all cases identical to a portion of a *pilS* copy. We were also able to detect antigenic variation in the *pilE* gene of *N. gonorrhoeae* strain MS11, and in agreement with an earlier report [9] this appeared to be occurring at a frequency lower than that observed for strain FA1090.

In agreement with earlier studies [1], [9] there are aspects of the antigenic variation that are difficult to explain. In the cases where it was possible to unambiguously identify the source of the donor variant sequence, it is clear that all silent loci can act in this capacity, and the number of variants generated was roughly proportional to the number of partial gene copies within the individual *pilS* loci. This was true for both strains FA1090 and MS11, and would seem to imply an underlying stochastic process. However we also observed two alleles arising in two separate experiments, again for both FA1090 and MS11. Similar results have been observed before [1], [9], exceed what might be expected by chance, and suggest some bias in the process of selection of donor sequences. There was also some bias evident in the partial gene copy, within a silent locus, that was used as a donor sequence. The copy at the 3′ end of the silent loci (copy 1) appears to be over-represented. For strain FA1090 5 of the 19 partial gene copies are designated as copy 1, so if all copies were used equally they may be expected to make up 26% of the donor sequences. In fact they make up 40% of the donor sequences. For strain MS11 this bias is even more pronounced. The use of copy 1 as a donor might be expected in 36% of the time whereas this was observed in 69% of cases.

A similar theme emerges when the variant frequency, rather than the number of variants, is examined. We summed the RecA^+^ frequencies of the individual variants to provide an overall frequency for all variants. For FA1090 this was 15.1%, and for MS11 3.6%. This is in reasonable agreement with previous studies using different methods where the variant frequency was estimated to be 12.9–13% for FA1090 [1], [9] and 5.7% for MS11 [9]. Again leaving aside those cases where a donor sequence could not be unambiguously identified, it seemed that partial gene copies 1 from the various *pilS* loci were again over-represented. For FA1090 they make up 53%, and for MS11 78%, of the unambiguous variant frequency.

The partial gene copies designated copy 1 differ from other silent copies in that they contain a sequence of approximately 250 nucleotides that has been designated the Pilus Associated Repeat (PAR) [15]. PAR is found immediately downstream of the end of the *pilE* gene, and each copy 1 of the *pilS* loci. Within the PAR in FA1090 are two previously described repeats, RS4 (32 nt) and the Sma/Cla repeat (65 nt) [15]. PAR is identically located in strain MS11, although some copies lack the RS4 repeat [16]. Previous work suggested that deletion of the Sma/Cla repeat downstream of the *pilE* gene results in a decrease in the amount of antigenic variation [18]. Our results suggest that PAR sequences associated with copy 1 in the various *pilS* loci influence both the source of the donor sequence and the frequency of antigenic variation associated with these specific partial gene copies.

It has previously been reported that antigenic variation is undetectable in the *pilE* gene of *N. meningitidis* strain FAM18 expressing class II pilin [3]. Despite potentially having the ability to detect variants at a much lower frequency than in the previous report, we were also unable to detect variation in this gene, or in the equivalent gene from *N. meningitidis* strain NMB. Strains producing class II pili have only two silent loci, as opposed to eight *pilS* copies in other meningococcal strains. Also the *pilE* gene expressing class II pilin is located elsewhere on the chromosome, whereas in those meningococcal strains that produce class I pili the silent loci are adjacent to *pilE*. Both of these factors might adversely affect the ability of the *pilE* gene from class II-producing strains to undergo antigenic variation. In addition, a DNA structure in the *pilE* promoter region of *N. gonorrhoeae* that is necessary for pilin antigenic variation has recently been described [12]. This guanine quartet structure is degenerate in meningococcal strains that produce class II pili, and this would also adversely affect the frequency of antigenic variation. However CKNM397 contains the *pilE* gene, and its native promoter, from strain MC58. The promoter region includes the sequences from MC58 that can form the guanine quartet structure. The absence of variation in this strain suggests that although this DNA structure is necessary for antigenic variation [12], by itself it is not sufficient to allow this process, at least in this genetic background. It seems that additional factor(s), present in class I-producing but not class II-producing strains, are needed for antigenic variation. Both FAM18 and NMB are disease-causing, rather than carriage, isolates. It therefore seems that antigenic variation of *pilE* is not essential for virulence, despite the fact that this process can be observed in all *N. gonorrhoeae* and many *N. meningitidis* strains.

We have also investigated another gene family where it has been suggested that antigenic variation might be occurring. In initial experiments with the *opaJ* gene of *N. gonorrhoeae* strain FA1090, we were unable to detect any variation. However it has been reported that the *opaK* gene, located close to *pilE*, is more variable than other *opa* genes [7]. In two separate experiments with *opaK* we were able to detect sequence variants. However only in the second experiment were they clearly more frequent when the RecA protein was present. There were also distinct differences from the variation observed in *pilE*. Firstly the number of different sequence variants detected was much smaller than seen in *pilE*. Secondly, compared with *pilE*, there was a more distinct bias in the source of the donor sequences. In every case the variant portion of the sequence was identical to part of the *opaD* gene, suggesting that this gene alone was donating sequence to yield variants of *opaK*.

Here we have demonstrated that PCR amplification of the genes of interest, followed by high throughput DNA sequencing, can be used to investigate antigenic variation. This approach could therefore be applied to a wide range of antigenic variation systems at a level that has not previously been possible. For example *Borrelia burgdorferi*, the causative agent of Lyme disease has a surface-exposed lipoprotein, VlsE, that undergoes antigenic variation [19], [20]. The Msp2 and Msp3 systems of *Anaplasma marginale* [21] and the VlhA system of *Mycoplasma synoviae* [22] are additional examples of antigenic variation of surface components that could be explored using this approach. These genes all contain both conserved and variable segments. Given the data depth that can be achieved using this approach, it should therefore be possible to undertake more systematic searches for conserved segments in these antigenically variable genes.

## Supporting Information

Figure S1 **K-mer depth across assemblies containing variant sequence segments.** The green trace depicts the k-mer depth in an amplicon derived from a culture grown in the presence of RecA, and therefore antigenic variation. The blue trace shows the k-mer depth for an amplicon obtained from a culture grown in the absence of RecA and therefore no antigenic variation. The vertical pale green bar shows the variant segment of the assembly across which the k-mer depth was averaged, and the vertical grey bars the same-sized conserved sequences on either side across which the k-mer depth was averaged, for the frequency calculation. **A.** An assembly containing a single variant segment. **B.** An assembly where a conserved region of more than k bases interrupts a variant segment, resulting in a spike of k-mer depth (marked with a downward arrow) that would result in an artificially high frequency calculation if the k-mer depth was averaged across the entire region.
(TIF)

Figure S2 **Alignment of the sequence of the** ***pilE*** **gene from the stock of** ***N. gonorrhoeae*** **strain FA1090 used in these experiments (top), and the FA1090 genome sequence (bottom).** Blue text indicates sequence flanking the *pilE* gene (black text). Sequence differences are highlighted in yellow. The grey shading highlights the extent of the sequence identity between the *pilE* genome sequence and *pilS1c1*, flanking the variant sequence.
(DOC)

Figure S3 **Alignment of the variant sequences detected in the first experiment with** ***pilE*** **in** ***N. gonorrhoeae*** **FA1090.** The allele 1 assembly is identical to the reference sequence obtained by Sanger sequencing of the amplicon. Blue text indicates sequence flanking the *pilE* gene (black text). Sequence differences are highlighted in yellow. The grey shading highlights the extent of the sequence identity between the *pilE* sequence and the various silent copies, flanking the variant sequence. Where the variant sequence was identical to part of two silent copies, the larger of the two regions of sequence identity is shown.
(DOC)

Figure S4 **Alignment of the variant sequences detected in the repeat experiment with** ***pilE*** **in** ***N. gonorrhoeae*** **FA1090.** The seq1 assembly is identical to the reference sequence obtained by Sanger sequencing of the amplicon. Blue text indicates sequence flanking the *pilE* gene (black text). Sequence differences are highlighted in yellow. The grey shading highlights the extent of the sequence identity between the *pilE* sequence and the various silent copies, flanking the variant sequence.
(DOC)

Figure S5 **Alignment of the variant sequences detected in the first experiment with** ***pilE*** **in** ***N. gonorrhoeae*** **MS11.** The allele 1 assembly is identical to the reference sequence obtained by Sanger sequencing of the amplicon. Blue text indicates sequence flanking the *pilE* gene (black text). Sequence differences are highlighted in yellow. The grey shading highlights the extent of the sequence identity between the *pilE* sequence and the various silent copies, flanking the variant sequence.
(DOC)

Figure S6 **Alignment of the variant sequences detected in the repeat experiment with** ***pilE*** **in** ***N. gonorrhoeae*** **MS11.** The allele 1 assembly is identical to the reference sequence obtained by Sanger sequencing of the amplicon. Blue text indicates sequence flanking the *pilE* gene (black text). Sequence differences are highlighted in yellow. The grey shading highlights the extent of the sequence identity between the *pilE* sequence and the various silent copies, flanking the variant sequence.
(DOC)

Figure S7 **Alignment of the variant sequences detected in the first experiment with** ***opaK*** **in** ***N. gonorrhoeae*** **FA1090.** The allele 1 assembly is identical to the reference sequence obtained by Sanger sequencing of the amplicon. Blue text indicates sequence flanking the *opaK* gene (black text). Sequence differences are highlighted in yellow.
(DOC)

Figure S8 **Alignment of the variant sequences detected in the repeat experiment with** ***opaK*** **in** ***N. gonorrhoeae*** **FA1090.** The allele 1 assembly is identical to the reference sequence obtained by Sanger sequencing of the amplicon. Blue text indicates sequence flanking the *opaK* gene (black text). Sequence differences are highlighted in yellow.
(DOC)

Table S1 **Oligonucleotide primers used to amplify the genes of interest.**
(DOC)

Table S2 **Sequence quality data.**
(DOC)

Text File S1 **Assembly of sequence reads.**
(DOC)
