# Supplementary figures and images for: The Use of High-Throughput DNA Sequencing in the Investigation of Antigenic Variation: Application to Neisseria Species
Source: PLoS One. 2014 Jan 22;9(1):e86704. doi: 10.1371/journal.pone.0086704 (PMC3899283; doi:10.1371/journal.pone.0086704)

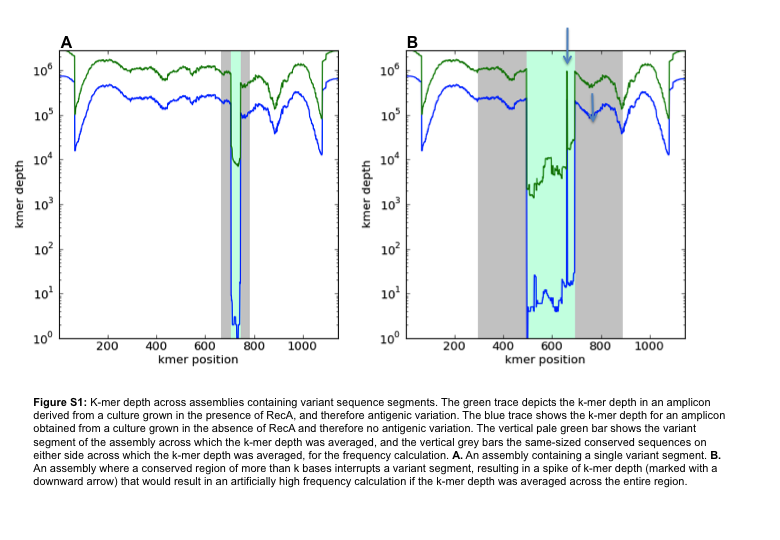

Supplement: Figure S1 — K-mer depth across assemblies containing variant sequence segments. The green trace depicts the k-mer depth in an amplicon derived from a culture grown in the presence of RecA, and therefore antigenic variation. The blue trace shows the k-mer depth for an amplicon obtained from a culture grown in the absence of RecA and therefore no antigenic variation. The vertical pale green bar shows the variant segment of the assembly across which the k-mer depth was averaged, and the vertical grey bars the same-sized conserved sequences on either side across which the k-mer depth was averaged, for the frequency calculation. A. An assembly containing a single variant segment. B. An assembly where a conserved region of more than k bases interrupts a variant segment, resulting in a spike of k-mer depth (marked with a downward arrow) that would result in an artificially high frequency calculation if the k-mer depth was averaged across the entire region. (TIF) [file pone.0086704.s001.tif]
