# Supplementary material for: The Use of High-Throughput DNA Sequencing in the Investigation of Antigenic Variation: Application to Neisseria Species
Source: PLoS One. 2014 Jan 22;9(1):e86704. doi: 10.1371/journal.pone.0086704 (PMC3899283; doi:10.1371/journal.pone.0086704)
Supplement: Figure S2 — Alignment of the sequence of the pilE gene from the stock of N. gonorrhoeae strain FA1090 used in these experiments (top), and the FA1090 genome sequence (bottom). Blue text indicates sequence flanking the pilE gene (black text). Sequence differences are highlighted in yellow. The grey shading highlights the extent of the sequence identity between the pilE genome sequence and pilS1c1, flanking the variant sequence. (DOC) [file pone.0086704.s002.doc]

PilE 1 TATTCTAACGCGTAAATTCAAAAATCTCAAATTCCGACCCAATCAACACACCCGATACCC 60

||||||||||||||||||||||||||||||||||||||||||||||||||||||||||||

Genome 2038359 TATTCTAACGCGTAAATTCAAAAATCTCAAATTCCGACCCAATCAACACACCCGATACCC 2038300

PilE 61 CATGCCAATAAAAAAGTAACGAAAATCGGCACTAAAACTGACAATTTTCGACACTGCCGC 120

||||||||||||||||||||||||||||||||||||||||||||||||||||||||||||

Genome 2038299 CATGCCAATAAAAAAGTAACGAAAATCGGCACTAAAACTGACAATTTTCGACACTGCCGC 2038240

PilE 121 CCCCTACTTCCGCAAACCACACCCACCtaaaagaaaatacaaaataaaaacaattatata 180

||||||||||||||||||||||||||||||||||||||||||||||||||||||||||||

Genome 2038239 CCCCTACTTCCGCAAACCACACCCACCTAAAAGAAAATACAAAATAAAAACAATTATATA 2038180

PilE 181 gagataaacgcataaaatttcacctcaaaacataaaatCGGCACGAATCTTGCTTTATAA 240

||||||||||||||||||||||||||||||||||||||||||||||||||||||||||||

Genome 2038179 GAGATAAACGCATAAAATTTCACCTCAAAACATAAAATCGGCACGAATCTTGCTTTATAA 2038120

PilE 241 TACGCAGTTGTCGCAACAAAAAACCGATGGTTAAATACATTGCATGATGCCGATGGCGTA 300

||||||||||||||||||||||||||||||||||||||||||||||||||||||||||||

Genome 2038119 TACGCAGTTGTCGCAACAAAAAACCGATGGTTAAATACATTGCATGATGCCGATGGCGTA 2038060

PilE 301 AGCCTGAGGCATTTCCCCTTTCAATTAGGAGTAATTTTATGAATACCCTTCAAAAAGGCT 360

||||||||||||||||||||||||||||||||||||||||||||||||||||||||||||

Genome 2038059 AGCCTGAGGCATTTCCCCTTTCAATTAGGAGTAATTTTATGAATACCCTTCAAAAAGGCT 2038000

PilE 361 TTACCCTTATCGAGCTGATGATTGTGATCGCTATCGTCGGCATTTTGGCGGCAGTCGCCC 420

||||||||||||||||||||||||||||||||||||||||||||||||||||||||||||

Genome 2037999 TTACCCTTATCGAGCTGATGATTGTGATCGCTATCGTCGGCATTTTGGCGGCAGTCGCCC 2037940

PilE 421 TTCCCGCCTACCAAGACTACACCGCCCGCGCGCAAGTTTCCGAAGCCATCCTTTTGGCCG 480

||||||||||||||||||||||||||||||||||||||||||||||||||||||||||||

Genome 2037939 TTCCCGCCTACCAAGACTACACCGCCCGCGCGCAAGTTTCCGAAGCCATCCTTTTGGCCG 2037880

PilE 481 AAGGTCAAAAATCAGCCGTTACCGGGTATTACCTGAATCACGGCATATGGCCGGAAGACA 540

||||||||||||||||||||||||||||||||||||||||||||||||||||||||||||

Genome 2037879 AAGGTCAAAAATCAGCCGTTACCGGGTATTACCTGAATCACGGCATATGGCCGGAAGACA 2037820

PilE 541 ACACTTCTGCCGGCGTGGCATcccccccTCCGACATCAAAGGCAAATATGTTCAAAGCGT 600

||||||||||||||||||||||||||||||||||||||||||||||||||||||||||||

Genome 2037819 ACACTTCTGCCGGCGTGGCATCCCCCCCTCCGACATCAAAGGCAAATATGTTCAAAGCGT 2037760

PilE 601 TACGGTCGCAAACGGCGTCGTTACCGCCGAAATGAAACCAAGCGGCGTAAACAAAGAAAT 660

||||||||||||||||||||||||||||||||||||| || ||||||||||||||||||

Genome 2037759 TACGGTCGCAAACGGCGTCGTTACCGCCGAAATGAAATCAGACGGCGTAAACAAAGAAAT 2037700

PilE 661 CAAAGGCAAAAAACTCTCCCTGTGGGCCAAGCGTGAAGACGGTTCGGTAAAATGGTTCTG 720

|||||||||||||||||||||||||| || |||| |||||||||||||||||||||||||

Genome 2037699 CAAAGGCAAAAAACTCTCCCTGTGGGGCAGGCGTCAAGACGGTTCGGTAAAATGGTTCTG 2037640

PilE 721 CGGACAGCCGGTTAAGCGCGACGCCGGCGCCAAAGCCGACGACGTCAAAGCCGACGCCGC 780

|||||||||||||| |||| | || |||||||||||||||||||||||||||||||||

Genome 2037639 CGGACAGCCGGTTACGCGCAA---CGACGCCAAAGCCGACGACGTCAAAGCCGACGCCGC 2037583

PilE 781 CAACGCCATCGAAACCAAGCACCTGCCGTCAACCTGCCGCGATGAATCATCTGCCACCTA 840

|||||||||||||||||||||||||||||||||||||||||||||| || |||||| ||

Genome 2037582 CAACGCCATCGAAACCAAGCACCTGCCGTCAACCTGCCGCGATGAACCAACTGCCAAATA 2037523

PilE 841 AGGCAAATTAGGCCTTAAATTTTAAATAAATCAAGCGGTAAGTGATTTCCCACGGCCGCC 900

|||||||||||||||||||||||||||||||||| |||||||||||||||||||||||||

Genome 2037522 AGGCAAATTAGGCCTTAAATTTTAAATAAATCAAACGGTAAGTGATTTCCCACGGCCGCC 2037463

PilE 901 CGGATCAACCCGGGCGGCTTGTCTTTTAAGGGTTTGCAAGGCGGGCGGGGTCGTCCGTTC 960

||||||||||||||||||||||||||||||||||||||||||||||||||||||||||||

Genome 2037462 CGGATCAACCCGGGCGGCTTGTCTTTTAAGGGTTTGCAAGGCGGGCGGGGTCGTCCGTTC 2037403

PilE 961 CGGTGGAAATAATATATCGATTGCGCTTCAAGGCCCTGCATGTGCCTCATTGCCACCCGT 1020

||||||||||||||||||||||||||||||||||||||||||||||||||||||||||||

Genome 2037402 CGGTGGAAATAATATATCGATTGCGCTTCAAGGCCCTGCATGTGCCTCATTGCCACCCGT 2037343

PilE 1021 TTAAACACGGTTTTTATCTGACAGGCGCGCAATCCGCCCCCTCATTTGTTAATCCGCCAT 1080

||||||||||||||||||||||||||||||||||||||||||||||||||||||||||||

Genome 2037342 TTAAACACGGTTTTTATCTGACAGGCGCGCAATCCGCCCCCTCATTTGTTAATCCGCCAT 2037283

PilE 1081 ATTGTATTGAAACACCGCCCGGAACCCGATATAATCCGCCCTTCAACATCAGTGAAAATC 1140

||||||||||||||||||||||||||||||||||||||||||||||||||||||||||||

Genome 2037282 ATTGTATTGAAACACCGCCCGGAACCCGATATAATCCGCCCTTCAACATCAGTGAAAATC 2037223

PilE 1141 tttttttAACCGGTTAAACCGAATAAGGAGCCGAA 1175

|||||||||||||||||||||||||||||||||||

Genome 2037222 TTTTTTTAACCGGTTAAACCGAATAAGGAGCCGAA 2037188

**Figure S2:** Alignment of the sequence of the *pilE* gene from the stock of *N. gonorrhoeae* strain FA1090 used in these experiments (top), and the FA1090 genome sequence (bottom). Blue text indicates sequence flanking the *pilE* gene (black text). Sequence differences are highlighted in yellow. The grey shading highlights the extent of the sequence identity between the *pilE* genome sequence and *pilS1c1*, flanking the variant sequence.
