# Supplementary material for: The Use of High-Throughput DNA Sequencing in the Investigation of Antigenic Variation: Application to Neisseria Species
Source: PLoS One. 2014 Jan 22;9(1):e86704. doi: 10.1371/journal.pone.0086704 (PMC3899283; doi:10.1371/journal.pone.0086704)
Supplement: Figure S3 — Alignment of the variant sequences detected in the first experiment with pilE in N. gonorrhoeae FA1090. The allele 1 assembly is identical to the reference sequence obtained by Sanger sequencing of the amplicon. Blue text indicates sequence flanking the pilE gene (black text). Sequence differences are highlighted in yellow. The grey shading highlights the extent of the sequence identity between the pilE sequence and the various silent copies, flanking the variant sequence. Where the variant sequence was identical to part of two silent copies, the larger of the two regions of sequence identity is shown. (DOC) [file pone.0086704.s003.doc]

allele 3 TATTCTAACGCGTAAATTCAAAAATCTCAAATTCCGACCCAATCAACACACCCGATACCC 60

allele 14 TATTCTAACGCGTAAATTCAAAAATCTCAAATTCCGACCCAATCAACACACCCGATACCC 60

allele 8 TATTCTAACGCGTAAATTCAAAAATCTCAAATTCCGACCCAATCAACACACCCGATACCC 60

allele 19 TATTCTAACGCGTAAATTCAAAAATCTCAAATTCCGACCCAATCAACACACCCGATACCC 60

allele 11 TATTCTAACGCGTAAATTCAAAAATCTCAAATTCCGACCCAATCAACACACCCGATACCC 60

allele 21 TATTCTAACGCGTAAATTCAAAAATCTCAAATTCCGACCCAATCAACACACCCGATACCC 60

allele 17 TATTCTAACGCGTAAATTCAAAAATCTCAAATTCCGACCCAATCAACACACCCGATACCC 60

allele 6 TATTCTAACGCGTAAATTCAAAAATCTCAAATTCCGACCCAATCAACACACCCGATACCC 60

allele 29 TATTCTAACGCGTAAATTCAAAAATCTCAAATTCCGACCCAATCAACACACCCGATACCC 60

allele 1 TATTCTAACGCGTAAATTCAAAAATCTCAAATTCCGACCCAATCAACACACCCGATACCC 60

allele 27 TATTCTAACGCGTAAATTCAAAAATCTCAAATTCCGACCCAATCAACACACCCGATACCC 60

allele 9 TATTCTAACGCGTAAATTCAAAAATCTCAAATTCCGACCCAATCAACACACCCGATACCC 60

allele 22 TATTCTAACGCGTAAATTCAAAAATCTCAAATTCCGACCCAATCAACACACCCGATACCC 60

allele 13 TATTCTAACGCGTAAATTCAAAAATCTCAAATTCCGACCCAATCAACACACCCGATACCC 60

allele 24 TATTCTAACGCGTAAATTCAAAAATCTCAAATTCCGACCCAATCAACACACCCGATACCC 60

allele 18 TATTCTAACGCGTAAATTCAAAAATCTCAAATTCCGACCCAATCAACACACCCGATACCC 60

allele 20 TATTCTAACGCGTAAATTCAAAAATCTCAAATTCCGACCCAATCAACACACCCGATACCC 60

allele 7 TATTCTAACGCGTAAATTCAAAAATCTCAAATTCCGACCCAATCAACACACCCGATACCC 60

allele 4 TATTCTAACGCGTAAATTCAAAAATCTCAAATTCCGACCCAATCAACACACCCGATACCC 60

allele 28 TATTCTAACGCGTAAATTCAAAAATCTCAAATTCCGACCCAATCAACACACCCGATACCC 60

allele 5 TATTCTAACGCGTAAATTCAAAAATCTCAAATTCCGACCCAATCAACACACCCGATACCC 60

allele 16 TATTCTAACGCGTAAATTCAAAAATCTCAAATTCCGACCCAATCAACACACCCGATACCC 60

allele 26 TATTCTAACGCGTAAATTCAAAAATCTCAAATTCCGACCCAATCAACACACCCGATACCC 60

allele 12 TATTCTAACGCGTAAATTCAAAAATCTCAAATTCCGACCCAATCAACACACCCGATACCC 60

allele 23 TATTCTAACGCGTAAATTCAAAAATCTCAAATTCCGACCCAATCAACACACCCGATACCC 60

allele 15 TATTCTAACGCGTAAATTCAAAAATCTCAAATTCCGACCCAATCAACACACCCGATACCC 60

allele 25 TATTCTAACGCGTAAATTCAAAAATCTCAAATTCCGACCCAATCAACACACCCGATACCC 60

allele 10 TATTCTAACGCGTAAATTCAAAAATCTCAAATTCCGACCCAATCAACACACCCGATACCC 60

allele 2 TATTCTAACGCGTAAATTCAAAAATCTCAAATTCCGACCCAATCAACACACCCGATACCC 60

************************************************************

allele 3 CATGCCAATAAAAAAGTAACGAAAATCGGCACTAAAACTGACAATTTTCGACACTGCCGC 120

allele 14 CATGCCAATAAAAAAGTAACGAAAATCGGCACTAAAACTGACAATTTTCGACACTGCCGC 120

allele 8 CATGCCAATAAAAAAGTAACGAAAATCGGCACTAAAACTGACAATTTTCGACACTGCCGC 120

allele 19 CATGCCAATAAAAAAGTAACGAAAATCGGCACTAAAACTGACAATTTTCGACACTGCCGC 120

allele 11 CATGCCAATAAAAAAGTAACGAAAATCGGCACTAAAACTGACAATTTTCGACACTGCCGC 120

allele 21 CATGCCAATAAAAAAGTAACGAAAATCGGCACTAAAACTGACAATTTTCGACACTGCCGC 120

allele 17 CATGCCAATAAAAAAGTAACGAAAATCGGCACTAAAACTGACAATTTTCGACACTGCCGC 120

allele 6 CATGCCAATAAAAAAGTAACGAAAATCGGCACTAAAACTGACAATTTTCGACACTGCCGC 120

allele 29 CATGCCAATAAAAAAGTAACGAAAATCGGCACTAAAACTGACAATTTTCGACACTGCCGC 120

allele 1 CATGCCAATAAAAAAGTAACGAAAATCGGCACTAAAACTGACAATTTTCGACACTGCCGC 120

allele 27 CATGCCAATAAAAAAGTAACGAAAATCGGCACTAAAACTGACAATTTTCGACACTGCCGC 120

allele 9 CATGCCAATAAAAAAGTAACGAAAATCGGCACTAAAACTGACAATTTTCGACACTGCCGC 120

allele 22 CATGCCAATAAAAAAGTAACGAAAATCGGCACTAAAACTGACAATTTTCGACACTGCCGC 120

allele 13 CATGCCAATAAAAAAGTAACGAAAATCGGCACTAAAACTGACAATTTTCGACACTGCCGC 120

allele 24 CATGCCAATAAAAAAGTAACGAAAATCGGCACTAAAACTGACAATTTTCGACACTGCCGC 120

allele 18 CATGCCAATAAAAAAGTAACGAAAATCGGCACTAAAACTGACAATTTTCGACACTGCCGC 120

allele 20 CATGCCAATAAAAAAGTAACGAAAATCGGCACTAAAACTGACAATTTTCGACACTGCCGC 120

allele 7 CATGCCAATAAAAAAGTAACGAAAATCGGCACTAAAACTGACAATTTTCGACACTGCCGC 120

allele 4 CATGCCAATAAAAAAGTAACGAAAATCGGCACTAAAACTGACAATTTTCGACACTGCCGC 120

allele 28 CATGCCAATAAAAAAGTAACGAAAATCGGCACTAAAACTGACAATTTTCGACACTGCCGC 120

allele 5 CATGCCAATAAAAAAGTAACGAAAATCGGCACTAAAACTGACAATTTTCGACACTGCCGC 120

allele 16 CATGCCAATAAAAAAGTAACGAAAATCGGCACTAAAACTGACAATTTTCGACACTGCCGC 120

allele 26 CATGCCAATAAAAAAGTAACGAAAATCGGCACTAAAACTGACAATTTTCGACACTGCCGC 120

allele 12 CATGCCAATAAAAAAGTAACGAAAATCGGCACTAAAACTGACAATTTTCGACACTGCCGC 120

allele 23 CATGCCAATAAAAAAGTAACGAAAATCGGCACTAAAACTGACAATTTTCGACACTGCCGC 120

allele 15 CATGCCAATAAAAAAGTAACGAAAATCGGCACTAAAACTGACAATTTTCGACACTGCCGC 120

allele 25 CATGCCAATAAAAAAGTAACGAAAATCGGCACTAAAACTGACAATTTTCGACACTGCCGC 120

allele 10 CATGCCAATAAAAAAGTAACGAAAATCGGCACTAAAACTGACAATTTTCGACACTGCCGC 120

allele 2 CATGCCAATAAAAAAGTAACGAAAATCGGCACTAAAACTGACAATTTTCGACACTGCCGC 120

************************************************************

allele 3 CCCCTACTTCCGCAAACCACACCCACCTAAAAGAAAATACAAAATAAAAACAATTATATA 180

allele 14 CCCCTACTTCCGCAAACCACACCCACCTAAAAGAAAATACAAAATAAAAACAATTATATA 180

allele 8 CCCCTACTTCCGCAAACCACACCCACCTAAAAGAAAATACAAAATAAAAACAATTATATA 180

allele 19 CCCCTACTTCCGCAAACCACACCCACCTAAAAGAAAATACAAAATAAAAACAATTATATA 180

allele 11 CCCCTACTTCCGCAAACCACACCCACCTAAAAGAAAATACAAAATAAAAACAATTATATA 180

allele 21 CCCCTACTTCCGCAAACCACACCCACCTAAAAGAAAATACAAAATAAAAACAATTATATA 180

allele 17 CCCCTACTTCCGCAAACCACACCCACCTAAAAGAAAATACAAAATAAAAACAATTATATA 180

allele 6 CCCCTACTTCCGCAAACCACACCCACCTAAAAGAAAATACAAAATAAAAACAATTATATA 180

allele 29 CCCCTACTTCCGCAAACCACACCCACCTAAAAGAAAATACAAAATAAAAACAATTATATA 180

allele 1 CCCCTACTTCCGCAAACCACACCCACCTAAAAGAAAATACAAAATAAAAACAATTATATA 180

allele 27 CCCCTACTTCCGCAAACCACACCCACCTAAAAGAAAATACAAAATAAAAACAATTATATA 180

allele 9 CCCCTACTTCCGCAAACCACACCCACCTAAAAGAAAATACAAAATAAAAACAATTATATA 180

allele 22 CCCCTACTTCCGCAAACCACACCCACCTAAAAGAAAATACAAAATAAAAACAATTATATA 180

allele 13 CCCCTACTTCCGCAAACCACACCCACCTAAAAGAAAATACAAAATAAAAACAATTATATA 180

allele 24 CCCCTACTTCCGCAAACCACACCCACCTAAAAGAAAATACAAAATAAAAACAATTATATA 180

allele 18 CCCCTACTTCCGCAAACCACACCCACCTAAAAGAAAATACAAAATAAAAACAATTATATA 180

allele 20 CCCCTACTTCCGCAAACCACACCCACCTAAAAGAAAATACAAAATAAAAACAATTATATA 180

allele 7 CCCCTACTTCCGCAAACCACACCCACCTAAAAGAAAATACAAAATAAAAACAATTATATA 180

allele 4 CCCCTACTTCCGCAAACCACACCCACCTAAAAGAAAATACAAAATAAAAACAATTATATA 180

allele 28 CCCCTACTTCCGCAAACCACACCCACCTAAAAGAAAATACAAAATAAAAACAATTATATA 180

allele 5 CCCCTACTTCCGCAAACCACACCCACCTAAAAGAAAATACAAAATAAAAACAATTATATA 180

allele 16 CCCCTACTTCCGCAAACCACACCCACCTAAAAGAAAATACAAAATAAAAACAATTATATA 180

allele 26 CCCCTACTTCCGCAAACCACACCCACCTAAAAGAAAATACAAAATAAAAACAATTATATA 180

allele 12 CCCCTACTTCCGCAAACCACACCCACCTAAAAGAAAATACAAAATAAAAACAATTATATA 180

allele 23 CCCCTACTTCCGCAAACCACACCCACCTAAAAGAAAATACAAAATAAAAACAATTATATA 180

allele 15 CCCCTACTTCCGCAAACCACACCCACCTAAAAGAAAATACAAAATAAAAACAATTATATA 180

allele 25 CCCCTACTTCCGCAAACCACACCCACCTAAAAGAAAATACAAAATAAAAACAATTATATA 180

allele 10 CCCCTACTTCCGCAAACCACACCCACCTAAAAGAAAATACAAAATAAAAACAATTATATA 180

allele 2 CCCCTACTTCCGCAAACCACACCCACCTAAAAGAAAATACAAAATAAAAACAATTATATA 180

************************************************************

allele 3 GAGATAAACGCATAAAATTTCACCTCAAAACATAAAATCGGCACGAATCTTGCTTTATAA 240

allele 14 GAGATAAACGCATAAAATTTCACCTCAAAACATAAAATCGGCACGAATCTTGCTTTATAA 240

allele 8 GAGATAAACGCATAAAATTTCACCTCAAAACATAAAATCGGCACGAATCTTGCTTTATAA 240

allele 19 GAGATAAACGCATAAAATTTCACCTCAAAACATAAAATCGGCACGAATCTTGCTTTATAA 240

allele 11 GAGATAAACGCATAAAATTTCACCTCAAAACATAAAATCGGCACGAATCTTGCTTTATAA 240

allele 21 GAGATAAACGCATAAAATTTCACCTCAAAACATAAAATCGGCACGAATCTTGCTTTATAA 240

allele 17 GAGATAAACGCATAAAATTTCACCTCAAAACATAAAATCGGCACGAATCTTGCTTTATAA 240

allele 6 GAGATAAACGCATAAAATTTCACCTCAAAACATAAAATCGGCACGAATCTTGCTTTATAA 240

allele 29 GAGATAAACGCATAAAATTTCACCTCAAAACATAAAATCGGCACGAATCTTGCTTTATAA 240

allele 1 GAGATAAACGCATAAAATTTCACCTCAAAACATAAAATCGGCACGAATCTTGCTTTATAA 240

allele 27 GAGATAAACGCATAAAATTTCACCTCAAAACATAAAATCGGCACGAATCTTGCTTTATAA 240

allele 9 GAGATAAACGCATAAAATTTCACCTCAAAACATAAAATCGGCACGAATCTTGCTTTATAA 240

allele 22 GAGATAAACGCATAAAATTTCACCTCAAAACATAAAATCGGCACGAATCTTGCTTTATAA 240

allele 13 GAGATAAACGCATAAAATTTCACCTCAAAACATAAAATCGGCACGAATCTTGCTTTATAA 240

allele 24 GAGATAAACGCATAAAATTTCACCTCAAAACATAAAATCGGCACGAATCTTGCTTTATAA 240

allele 18 GAGATAAACGCATAAAATTTCACCTCAAAACATAAAATCGGCACGAATCTTGCTTTATAA 240

allele 20 GAGATAAACGCATAAAATTTCACCTCAAAACATAAAATCGGCACGAATCTTGCTTTATAA 240

allele 7 GAGATAAACGCATAAAATTTCACCTCAAAACATAAAATCGGCACGAATCTTGCTTTATAA 240

allele 4 GAGATAAACGCATAAAATTTCACCTCAAAACATAAAATCGGCACGAATCTTGCTTTATAA 240

allele 28 GAGATAAACGCATAAAATTTCACCTCAAAACATAAAATCGGCACGAATCTTGCTTTATAA 240

allele 5 GAGATAAACGCATAAAATTTCACCTCAAAACATAAAATCGGCACGAATCTTGCTTTATAA 240

allele 16 GAGATAAACGCATAAAATTTCACCTCAAAACATAAAATCGGCACGAATCTTGCTTTATAA 240

allele 26 GAGATAAACGCATAAAATTTCACCTCAAAACATAAAATCGGCACGAATCTTGCTTTATAA 240

allele 12 GAGATAAACGCATAAAATTTCACCTCAAAACATAAAATCGGCACGAATCTTGCTTTATAA 240

allele 23 GAGATAAACGCATAAAATTTCACCTCAAAACATAAAATCGGCACGAATCTTGCTTTATAA 240

allele 15 GAGATAAACGCATAAAATTTCACCTCAAAACATAAAATCGGCACGAATCTTGCTTTATAA 240

allele 25 GAGATAAACGCATAAAATTTCACCTCAAAACATAAAATCGGCACGAATCTTGCTTTATAA 240

allele 10 GAGATAAACGCATAAAATTTCACCTCAAAACATAAAATCGGCACGAATCTTGCTTTATAA 240

allele 2 GAGATAAACGCATAAAATTTCACCTCAAAACATAAAATCGGCACGAATCTTGCTTTATAA 240

************************************************************

allele 3 TACGCAGTTGTCGCAACAAAAAACCGATGGTTAAATACATTGCATGATGCCGATGGCGTA 300

allele 14 TACGCAGTTGTCGCAACAAAAAACCGATGGTTAAATACATTGCATGATGCCGATGGCGTA 300

allele 8 TACGCAGTTGTCGCAACAAAAAACCGATGGTTAAATACATTGCATGATGCCGATGGCGTA 300

allele 19 TACGCAGTTGTCGCAACAAAAAACCGATGGTTAAATACATTGCATGATGCCGATGGCGTA 300

allele 11 TACGCAGTTGTCGCAACAAAAAACCGATGGTTAAATACATTGCATGATGCCGATGGCGTA 300

allele 21 TACGCAGTTGTCGCAACAAAAAACCGATGGTTAAATACATTGCATGATGCCGATGGCGTA 300

allele 17 TACGCAGTTGTCGCAACAAAAAACCGATGGTTAAATACATTGCATGATGCCGATGGCGTA 300

allele 6 TACGCAGTTGTCGCAACAAAAAACCGATGGTTAAATACATTGCATGATGCCGATGGCGTA 300

allele 29 TACGCAGTTGTCGCAACAAAAAACCGATGGTTAAATACATTGCATGATGCCGATGGCGTA 300

allele 1 TACGCAGTTGTCGCAACAAAAAACCGATGGTTAAATACATTGCATGATGCCGATGGCGTA 300

allele 27 TACGCAGTTGTCGCAACAAAAAACCGATGGTTAAATACATTGCATGATGCCGATGGCGTA 300

allele 9 TACGCAGTTGTCGCAACAAAAAACCGATGGTTAAATACATTGCATGATGCCGATGGCGTA 300

allele 22 TACGCAGTTGTCGCAACAAAAAACCGATGGTTAAATACATTGCATGATGCCGATGGCGTA 300

allele 13 TACGCAGTTGTCGCAACAAAAAACCGATGGTTAAATACATTGCATGATGCCGATGGCGTA 300

allele 24 TACGCAGTTGTCGCAACAAAAAACCGATGGTTAAATACATTGCATGATGCCGATGGCGTA 300

allele 18 TACGCAGTTGTCGCAACAAAAAACCGATGGTTAAATACATTGCATGATGCCGATGGCGTA 300

allele 20 TACGCAGTTGTCGCAACAAAAAACCGATGGTTAAATACATTGCATGATGCCGATGGCGTA 300

allele 7 TACGCAGTTGTCGCAACAAAAAACCGATGGTTAAATACATTGCATGATGCCGATGGCGTA 300

allele 4 TACGCAGTTGTCGCAACAAAAAACCGATGGTTAAATACATTGCATGATGCCGATGGCGTA 300

allele 28 TACGCAGTTGTCGCAACAAAAAACCGATGGTTAAATACATTGCATGATGCCGATGGCGTA 300

allele 5 TACGCAGTTGTCGCAACAAAAAACCGATGGTTAAATACATTGCATGATGCCGATGGCGTA 300

allele 16 TACGCAGTTGTCGCAACAAAAAACCGATGGTTAAATACATTGCATGATGCCGATGGCGTA 300

allele 26 TACGCAGTTGTCGCAACAAAAAACCGATGGTTAAATACATTGCATGATGCCGATGGCGTA 300

allele 12 TACGCAGTTGTCGCAACAAAAAACCGATGGTTAAATACATTGCATGATGCCGATGGCGTA 300

allele 23 TACGCAGTTGTCGCAACAAAAAACCGATGGTTAAATACATTGCATGATGCCGATGGCGTA 300

allele 15 TACGCAGTTGTCGCAACAAAAAACCGATGGTTAAATACATTGCATGATGCCGATGGCGTA 300

allele 25 TACGCAGTTGTCGCAACAAAAAACCGATGGTTAAATACATTGCATGATGCCGATGGCGTA 300

allele 10 TACGCAGTTGTCGCAACAAAAAACCGATGGTTAAATACATTGCATGATGCCGATGGCGTA 300

allele 2 TACGCAGTTGTCGCAACAAAAAACCGATGGTTAAATACATTGCATGATGCCGATGGCGTA 300

************************************************************

allele 3 AGCCTGAGGCATTTCCCCTTTCAATTAGGAGTAATTTTATGAATACCCTTCAAAAAGGCT 360

allele 14 AGCCTGAGGCATTTCCCCTTTCAATTAGGAGTAATTTTATGAATACCCTTCAAAAAGGCT 360

allele 8 AGCCTGAGGCATTTCCCCTTTCAATTAGGAGTAATTTTATGAATACCCTTCAAAAAGGCT 360

allele 19 AGCCTGAGGCATTTCCCCTTTCAATTAGGAGTAATTTTATGAATACCCTTCAAAAAGGCT 360

allele 11 AGCCTGAGGCATTTCCCCTTTCAATTAGGAGTAATTTTATGAATACCCTTCAAAAAGGCT 360

allele 21 AGCCTGAGGCATTTCCCCTTTCAATTAGGAGTAATTTTATGAATACCCTTCAAAAAGGCT 360

allele 17 AGCCTGAGGCATTTCCCCTTTCAATTAGGAGTAATTTTATGAATACCCTTCAAAAAGGCT 360

allele 6 AGCCTGAGGCATTTCCCCTTTCAATTAGGAGTAATTTTATGAATACCCTTCAAAAAGGCT 360

allele 29 AGCCTGAGGCATTTCCCCTTTCAATTAGGAGTAATTTTATGAATACCCTTCAAAAAGGCT 360

allele 1 AGCCTGAGGCATTTCCCCTTTCAATTAGGAGTAATTTTATGAATACCCTTCAAAAAGGCT 360

allele 27 AGCCTGAGGCATTTCCCCTTTCAATTAGGAGTAATTTTATGAATACCCTTCAAAAAGGCT 360

allele 9 AGCCTGAGGCATTTCCCCTTTCAATTAGGAGTAATTTTATGAATACCCTTCAAAAAGGCT 360

allele 22 AGCCTGAGGCATTTCCCCTTTCAATTAGGAGTAATTTTATGAATACCCTTCAAAAAGGCT 360

allele 13 AGCCTGAGGCATTTCCCCTTTCAATTAGGAGTAATTTTATGAATACCCTTCAAAAAGGCT 360

allele 24 AGCCTGAGGCATTTCCCCTTTCAATTAGGAGTAATTTTATGAATACCCTTCAAAAAGGCT 360

allele 18 AGCCTGAGGCATTTCCCCTTTCAATTAGGAGTAATTTTATGAATACCCTTCAAAAAGGCT 360

allele 20 AGCCTGAGGCATTTCCCCTTTCAATTAGGAGTAATTTTATGAATACCCTTCAAAAAGGCT 360

allele 7 AGCCTGAGGCATTTCCCCTTTCAATTAGGAGTAATTTTATGAATACCCTTCAAAAAGGCT 360

allele 4 AGCCTGAGGCATTTCCCCTTTCAATTAGGAGTAATTTTATGAATACCCTTCAAAAAGGCT 360

allele 28 AGCCTGAGGCATTTCCCCTTTCAATTAGGAGTAATTTTATGAATACCCTTCAAAAAGGCT 360

allele 5 AGCCTGAGGCATTTCCCCTTTCAATTAGGAGTAATTTTATGAATACCCTTCAAAAAGGCT 360

allele 16 AGCCTGAGGCATTTCCCCTTTCAATTAGGAGTAATTTTATGAATACCCTTCAAAAAGGCT 360

allele 26 AGCCTGAGGCATTTCCCCTTTCAATTAGGAGTAATTTTATGAATACCCTTCAAAAAGGCT 360

allele 12 AGCCTGAGGCATTTCCCCTTTCAATTAGGAGTAATTTTATGAATACCCTTCAAAAAGGCT 360

allele 23 AGCCTGAGGCATTTCCCCTTTCAATTAGGAGTAATTTTATGAATACCCTTCAAAAAGGCT 360

allele 15 AGCCTGAGGCATTTCCCCTTTCAATTAGGAGTAATTTTATGAATACCCTTCAAAAAGGCT 360

allele 25 AGCCTGAGGCATTTCCCCTTTCAATTAGGAGTAATTTTATGAATACCCTTCAAAAAGGCT 360

allele 10 AGCCTGAGGCATTTCCCCTTTCAATTAGGAGTAATTTTATGAATACCCTTCAAAAAGGCT 360

allele 2 AGCCTGAGGCATTTCCCCTTTCAATTAGGAGTAATTTTATGAATACCCTTCAAAAAGGCT 360

************************************************************

allele 3 TTACCCTTATCGAGCTGATGATTGTGATCGCTATCGTCGGCATTTTGGCGGCAGTCGCCC 420

allele 14 TTACCCTTATCGAGCTGATGATTGTGATCGCTATCGTCGGCATTTTGGCGGCAGTCGCCC 420

allele 8 TTACCCTTATCGAGCTGATGATTGTGATCGCTATCGTCGGCATTTTGGCGGCAGTCGCCC 420

allele 19 TTACCCTTATCGAGCTGATGATTGTGATCGCTATCGTCGGCATTTTGGCGGCAGTCGCCC 420

allele 11 TTACCCTTATCGAGCTGATGATTGTGATCGCTATCGTCGGCATTTTGGCGGCAGTCGCCC 420

allele 21 TTACCCTTATCGAGCTGATGATTGTGATCGCTATCGTCGGCATTTTGGCGGCAGTCGCCC 420

allele 17 TTACCCTTATCGAGCTGATGATTGTGATCGCTATCGTCGGCATTTTGGCGGCAGTCGCCC 420

allele 6 TTACCCTTATCGAGCTGATGATTGTGATCGCTATCGTCGGCATTTTGGCGGCAGTCGCCC 420

allele 29 TTACCCTTATCGAGCTGATGATTGTGATCGCTATCGTCGGCATTTTGGCGGCAGTCGCCC 420

allele 1 TTACCCTTATCGAGCTGATGATTGTGATCGCTATCGTCGGCATTTTGGCGGCAGTCGCCC 420

allele 27 TTACCCTTATCGAGCTGATGATTGTGATCGCTATCGTCGGCATTTTGGCGGCAGTCGCCC 420

allele 9 TTACCCTTATCGAGCTGATGATTGTGATCGCTATCGTCGGCATTTTGGCGGCAGTCGCCC 420

allele 22 TTACCCTTATCGAGCTGATGATTGTGATCGCTATCGTCGGCATTTTGGCGGCAGTCGCCC 420

allele 13 TTACCCTTATCGAGCTGATGATTGTGATCGCTATCGTCGGCATTTTGGCGGCAGTCGCCC 420

allele 24 TTACCCTTATCGAGCTGATGATTGTGATCGCTATCGTCGGCATTTTGGCGGCAGTCGCCC 420

allele 18 TTACCCTTATCGAGCTGATGATTGTGATCGCTATCGTCGGCATTTTGGCGGCAGTCGCCC 420

allele 20 TTACCCTTATCGAGCTGATGATTGTGATCGCTATCGTCGGCATTTTGGCGGCAGTCGCCC 420

allele 7 TTACCCTTATCGAGCTGATGATTGTGATCGCTATCGTCGGCATTTTGGCGGCAGTCGCCC 420

allele 4 TTACCCTTATCGAGCTGATGATTGTGATCGCTATCGTCGGCATTTTGGCGGCAGTCGCCC 420

allele 28 TTACCCTTATCGAGCTGATGATTGTGATCGCTATCGTCGGCATTTTGGCGGCAGTCGCCC 420

allele 5 TTACCCTTATCGAGCTGATGATTGTGATCGCTATCGTCGGCATTTTGGCGGCAGTCGCCC 420

allele 16 TTACCCTTATCGAGCTGATGATTGTGATCGCTATCGTCGGCATTTTGGCGGCAGTCGCCC 420

allele 26 TTACCCTTATCGAGCTGATGATTGTGATCGCTATCGTCGGCATTTTGGCGGCAGTCGCCC 420

allele 12 TTACCCTTATCGAGCTGATGATTGTGATCGCTATCGTCGGCATTTTGGCGGCAGTCGCCC 420

allele 23 TTACCCTTATCGAGCTGATGATTGTGATCGCTATCGTCGGCATTTTGGCGGCAGTCGCCC 420

allele 15 TTACCCTTATCGAGCTGATGATTGTGATCGCTATCGTCGGCATTTTGGCGGCAGTCGCCC 420

allele 25 TTACCCTTATCGAGCTGATGATTGTGATCGCTATCGTCGGCATTTTGGCGGCAGTCGCCC 420

allele 10 TTACCCTTATCGAGCTGATGATTGTGATCGCTATCGTCGGCATTTTGGCGGCAGTCGCCC 420

allele 2 TTACCCTTATCGAGCTGATGATTGTGATCGCTATCGTCGGCATTTTGGCGGCAGTCGCCC 420

************************************************************

allele 3 TTCCCGCCTACCAAGACTACACCGCCCGCGCGCAAGTTTCCGAAGCCATCCTTTTGGCCG 480

allele 14 TTCCCGCCTACCAAGACTACACCGCCCGCGCGCAAGTTTCCGAAGCCATCCTTTTGGCCG 480

allele 8 TTCCCGCCTACCAAGACTACACCGCCCGCGCGCAAGTTTCCGAAGCCATCCTTTTGGCCG 480

allele 19 TTCCCGCCTACCAAGACTACACCGCCCGCGCGCAAGTTTCCGAAGCCATCCTTTTGGCCG 480

allele 11 TTCCCGCCTACCAAGACTACACCGCCCGCGCGCAAGTTTCCGAAGCCATCCTTTTGGCCG 480

allele 21 TTCCCGCCTACCAAGACTACACCGCCCGCGCGCAAGTTTCCGAAGCCATCCTTTTGGCCG 480

allele 17 TTCCCGCCTACCAAGACTACACCGCCCGCGCGCAAGTTTCCGAAGCCATCCTTTTGGCCG 480

allele 6 TTCCCGCCTACCAAGACTACACCGCCCGCGCGCAAGTTTCCGAAGCCATCCTTTTGGCCG 480

allele 29 TTCCCGCCTACCAAGACTACACCGCCCGCGCGCAAGTTTCCGAAGCCATCCTTTTGGCCG 480

allele 1 TTCCCGCCTACCAAGACTACACCGCCCGCGCGCAAGTTTCCGAAGCCATCCTTTTGGCCG 480

allele 27 TTCCCGCCTACCAAGACTACACCGCCCGCGCGCAAGTTTCCGAAGCCATCCTTTTGGCCG 480

allele 9 TTCCCGCCTACCAAGACTACACCGCCCGCGCGCAAGTTTCCGAAGCCATCCTTTTGGCCG 480

allele 22 TTCCCGCCTACCAAGACTACACCGCCCGCGCGCAAGTTTCCGAAGCCATCCTTTTGGCCG 480

allele 13 TTCCCGCCTACCAAGACTACACCGCCCGCGCGCAAGTTTCCGAAGCCATCCTTTTGGCCG 480

allele 24 TTCCCGCCTACCAAGACTACACCGCCCGCGCGCAAGTTTCCGAAGCCATCCTTTTGGCCG 480

allele 18 TTCCCGCCTACCAAGACTACACCGCCCGCGCGCAAGTTTCCGAAGCCATCCTTTTGGCCG 480

allele 20 TTCCCGCCTACCAAGACTACACCGCCCGCGCGCAAGTTTCCGAAGCCATCCTTTTGGCCG 480

allele 7 TTCCCGCCTACCAAGACTACACCGCCCGCGCGCAAGTTTCCGAAGCCATCCTTTTGGCCG 480

allele 4 TTCCCGCCTACCAAGACTACACCGCCCGCGCGCAAGTTTCCGAAGCCATCCTTTTGGCCG 480

allele 28 TTCCCGCCTACCAAGACTACACCGCCCGCGCGCAAGTTTCCGAAGCCATCCTTTTGGCCG 480

allele 5 TTCCCGCCTACCAAGACTACACCGCCCGCGCGCAAGTTTCCGAAGCCATCCTTTTGGCCG 480

allele 16 TTCCCGCCTACCAAGACTACACCGCCCGCGCGCAAGTTTCCGAAGCCATCCTTTTGGCCG 480

allele 26 TTCCCGCCTACCAAGACTACACCGCCCGCGCGCAAGTTTCCGAAGCCATCCTTTTGGCCG 480

allele 12 TTCCCGCCTACCAAGACTACACCGCCCGCGCGCAAGTTTCCGAAGCCATCCTTTTGGCCG 480

allele 23 TTCCCGCCTACCAAGACTACACCGCCCGCGCGCAAGTTTCCGAAGCCATCCTTTTGGCCG 480

allele 15 TTCCCGCCTACCAAGACTACACCGCCCGCGCGCAAGTTTCCGAAGCCATCCTTTTGGCCG 480

allele 25 TTCCCGCCTACCAAGACTACACCGCCCGCGCGCAAGTTTCCGAAGCCATCCTTTTGGCCG 480

allele 10 TTCCCGCCTACCAAGACTACACCGCCCGCGCGCAAGTTTCCGAAGCCATCCTTTTGGCCG 480

allele 2 TTCCCGCCTACCAAGACTACACCGCCCGCGCGCAAGTTTCCGAAGCCATCCTTTTGGCCG 480

************************************************************

allele 3 AAGGTCAAAAATCAGCCGTTACCGGGTATTACCTGAATCACGGCATATGGCCGGAAGACA 540

allele 14 AAGGTCAAAAATCAGCCGTTACCGGGTATTACCTGAATCACGGCATATGGCCGGAAGACA 540

allele 8 AAGGTCAAAAATCAGCCGTTACCGGGTATTACCTGAATCACGGCATATGGCCGGAAGACA 540

allele 19 AAGGTCAAAAATCAGCCGTTACCGGGTATTACCTGAATCACGGCATATGGCCGGAAGACA 540

allele 11 AAGGTCAAAAATCAGCCGTTACCGGGTATTACCTGAATCACGGCATATGGCCGGAAGACA 540

allele 21 AAGGTCAAAAATCAGCCGTTACCGGGTATTACCTGAATCACGGCATATGGCCGGAAGACA 540

allele 17 AAGGTCAAAAATCAGCCGTTACCGGGTATTACCTGAATCACGGCATATGGCCGGAAGACA 540

allele 6 AAGGTCAAAAATCAGCCGTTACCGGGTATTACCTGAATCACGGCATATGGCCGGAAGACA 540

allele 29 AAGGTCAAAAATCAGCCGTTACCGGGTATTACCTGAATCACGGCATATGGCCGGAAGACA 540

allele 1 AAGGTCAAAAATCAGCCGTTACCGGGTATTACCTGAATCACGGCATATGGCCGGAAGACA 540

allele 27 AAGGTCAAAAATCAGCCGTTACCGGGTATTACCTGAATCACGGCATATGGCCGGAAGACA 540

allele 9 AAGGTCAAAAATCAGCCGTTACCGGGTATTACCTGAATCACGGCATATGGCCGGAAGACA 540

allele 22 AAGGTCAAAAATCAGCCGTTACCGGGTATTACCTGAATCACGGCATATGGCCGGAAGACA 540

allele 13 AAGGTCAAAAATCAGCCGTTACCGGGTATTACCTGAATCACGGCATATGGCCGGAAGACA 540

allele 24 AAGGTCAAAAATCAGCCGTTACCGGGTATTACCTGAATCACGGCATATGGCCCAAAGACA 540

allele 18 AAGGTCAAAAATCAGCCGTTACCGGGTATTACCTGAATCACGGCATATGGCCGGAAGACA 540

allele 20 AAGGTCAAAAATCAGCCGTCACCGAGTATTACCTGAATCACGGCACATGGCCGGAAAACA 540

allele 7 AAGGTCAAAAATCAGCCGTTACCGGGTATTACCTGAATCACGGCATATGGCCGGAAGACA 540

allele 4 AAGGTCAAAAATCAGCCGTTACCGGGTATTACCTGAATCACGGCATATGGCCGGAAGACA 540

allele 28 AAGGTCAAAAATCAGCCGTTACCGGGTATTACCTGAATCACGGCATATGGCCGGAAGACA 540

allele 5 AAGGTCAAAAATCAGCCGTTACCGAGTATTACCTGAATCACGGCATATGGCCGAAAGACA 540

allele 16 AAGGTCAAAAATCAGCCGTCACCGAGTATTACCTGAATCACGGCATATGGCCGAAAGACA 540

allele 26 AAGGTCAAAAATCAGCCGTTACCGGGTATTACCTGAATCACGGCATATGGCCGGAAGACA 540

allele 12 AAGGTCAAAAATCAGCCGTTACCGGGTATTACCTGAATCACGGCATATGGCCGGAAGACA 540

allele 23 AAGGTCAAAAATCAGCCGTTACCGGGTATTACCTGAATCACGGCACATGGCCGAAAGACA 540

allele 15 AAGGTCAAAAATCAGCCGTTACCGAGTATTACCTGAATCACGGCGAATGGCCCAAAGACA 540

allele 25 AAGGTCAAAAATCAGCCGTTACCGGGTATTACCTGAATCACGGCATATGGCCGGAAAACA 540

allele 10 AAGGTCAAAAATCAGCCGTTACCGGGTATTACCTGAATAACGGCAAATGGCCCGCCGACA 540

allele 2 AAGGTCAAAAATCAGCCGTTACCGGGTATTACCTGAATCACGGCGAATGGCCGGAAAACA 540

******************* **** ************* ***** ****** ***

allele 3 ACACTTCTGCCGGCGTGGCATCCCCC-CCTCCG--ACATCAAAGGCAAATATGTTCAAAG 597

allele 14 ACACTTCTGCCGGCGTGGCATCCCCC-CCTCCG--ACATCAAAGGCAAATATGTTCAAAG 597

allele 8 ACACTTCTGCCGGCGTGGCATCCCCC-CCTCCG--ACATCAAAGGCAAATATGTTCAAAG 597

allele 19 ACACTTCTGCCGGCGTGGCATCCCCC-CCTCCG--ACATCAAAGGCAAATATGTTCAAAG 597

allele 11 ACACTTCTGCCGGCGTGGCATCCCCC-CCTCCG--ACATCAAAGGCAAATATGTTCAAAG 597

allele 21 ACACTTCTGCCGGCGTGGCATCCCCC-CCTCCG--ACATCAAAGGCAAATATGTTCAAAG 597

allele 17 ACACTTCTGCCGGCGTGGCATCCCCC-CCTCCG--ACATCAAAGGCAAATATGTTCAAAG 597

allele 6 ACACTTCTGCCGGCGTGGCATCCCCC-CCTCCG--ACATCAAAGGCAAATATGTTCAAAG 597

allele 29 ACACTTCTGCCGGCGTGGCATCCCCC-CCTCCG--ACATCAAAGGCAAATATGTTCAAAG 597

allele 1 ACACTTCTGCCGGCGTGGCATCCCCC-CCTCCG--ACATCAAAGGCAAATATGTTCAAAG 597

allele 27 ACACTTCTGCCGGCGTGGCATCCCCC-CCTCCG--ACATCAAAGGCAAATATGTTCAAAG 597

allele 9 ACACTTCTGCCGGCGTGGCATCCCCC-CCTCCG--ACATCAAAGGCAAATATGTTCAAAG 597

allele 22 ACACTTCTGCCGGCGTGGCATCCCCC-CCTCCG--ACATCAAAGGCAAATATGTTCAAAG 597

allele 13 ACACTTCTGCCGGCGTGGCATCCCCC-CCTCCG--ACATCAAAGGCAAATATGTTCAAAG 597

allele 24 ACGGCTCTGCCGGCGTGGCATCCCCC-CCTCCG--ACATCAAAGGCAAATATGTTCAAAG 597

allele 18 ACACTTCTGCCGGCGTGGCATCCCCCGCC---G--AAATCAAAGGCAAATATGTTCAAAG 595

allele 20 ACACTTCTGCCGGCGTGGCATCCTCCGCCACCG--ACATCAAAGGCAAATATGTTCAAAG 598

allele 7 ACACTTCTGCCGGCGTGGCATCCCCC-CCTCCG--ACATCAAAGGCAAATATGTTCAAAG 597

allele 4 ACACTTCTGCCGGCGTGGCATCCCCC-CCACCG--ACATCAAAGGCAAATATGTTAAAAG 597

allele 28 ACACTTCTGCCGGCGTGGCATCCCCC-CCTCCG--ACATCAAAGGCAAATATGTTCAAAG 597

allele 5 ACACTTCTGCCGGCGTGGCAAACCCC-AC--CG--ACATCAAAGGCAAATATGTTGAAAG 595

allele 16 ACACTTCTGCCGGCGTGGCAAACCCC-AC--CG--ACATCAAAGGCAAATATGTTGAAAG 595

allele 26 ACACTTCTGCCGGCGTGGCATCCCCC-CCTCCG--ACATCAAAGGCAAATATGTTCAAAG 597

allele 12 ACGGTGATGCCGGCGTGGCATCCCCCGCCGACA--AAATCAAAGGCAAATATGTTCAGAA 598

allele 23 ACGGTGATGCCGGCGTGGCATCCCCCGCCGACA--AAATCAAAGGCAAATATGTTCAGAA 598

allele 15 ACGGCTCTGCCGGCGTGGCATCC---GCTTCAA--AAATCATAGGCAAATATGTTAAGGA 595

allele 25 ACGACAAGGCCGGCGTGGCATCT---TCTTCAT--CAATCAAAGGCAAATATGTTAAGGA 595

allele 10 ACGGCGCTGCCGGCGTGGCATCCCCCGCCACCG--ACATCAAAGGCAAATATGTTAAGGA 598

allele 2 ACACTTCTGCCGGCGTGGCATCC-----TCCGACAAAATCAAAGGCAAATATGTTCAGAA 595

** ************ **** ************* *

allele 3 CGTTACGGTCGCAAACGGCGTCGTTACCGCCGAAATGAAACCAAGCGGCGTAAACAAAGA 657

allele 14 CGTTACGGTCGCAAACGGCGTCGTTACCGCCGAAATGAAACCAAGCGGCGTAAACAAAGA 657

allele 8 CGTTACGGTCGCAAACGGCGTCGTTACCGCCGAAATGAAACCAAGCGGCGTAAACAAAGA 657

allele 19 CGTTACGGTCGCAAACGGCGTCGTTACCGCCGAAATGAAACCAAGCGGCGTAAACAAAGA 657

allele 11 CGTTACGGTCGCAAACGGCGTCGTTACCGCCGAAATGAAACCAAGCGGCGTAAACAAAGA 657

allele 21 CGTTACGGTCGCAAACGGCGTCGTTACCGCCGAAATGAAACCAAGCGGCGTAAACAAAGA 657

allele 17 CGTTACGGTCGCAAACGGCGTCGTTACCGCCGAAATGAAACCAAGCGGCGTAAACAAAGA 657

allele 6 CGTTACGGTCGCAAACGGCGTCGTTACCGCCGAAATGAAACCAAGCGGCGTAAACAAAGA 657

allele 29 CGTTACGGTCGCAAACGGCGTCGTTACCGCCGAAATGAAACCAAGCGGCGTAAACAAAGA 657

allele 1 CGTTACGGTCGCAAACGGCGTCGTTACCGCCGAAATGAAACCAAGCGGCGTAAACAAAGA 657

allele 27 CGTTACGGTCGCAAACGGCGTCGTTACCGCCGAAATGAAACCAAGCGGCGTAAACAAAGA 657

allele 9 CGTTACGGTCGCAAACGGCGTCGTTACCGCCGAAATGAAATCAGACGGCGTAAACAAAGA 657

allele 22 CGTTACGGTCGCAAACGGCGTCGTTACCGCCGAAATGAAATCAGACGGCGTAAACAAAGA 657

allele 13 CGTTACGGTCGCAAACGGCGTCGTTACCGCCGAAATGAAACCAAGCGGCGTAAACAAAGA 657

allele 24 CGTTACGGTCGCAAACGGCGTCGTTACCGCCGAAATGAAACCAAGCGGCGTAAACAAAGA 657

allele 18 CGTTACGGTCGCAAACGGCGTCGTTACCGCCGAAATGAAACCAAGCGGCGTAAACAAAGA 655

allele 20 CGTTACGGTCGCAAACGGCGTCGTTACCGCCGAAATGAAACCAAGCGGCGTAAACAAAGA 658

allele 7 CGTTACGGTCGCAAAAGGCGTCGTTACCGCCCAAATGAATCCAAGCGGCGTAAACAATGA 657

allele 4 CGTTACGGTCGCAAAAGGCGTCGTCACCGCCGAAATGGCTTCAACCGGCGTAAACAATGA 657

allele 28 CGTTACGGTCGCAAACGGCGTCGTTACCGCCCAAATGGCTTCAACCGGCGTAAACAAAGA 657

allele 5 CGTTACGGTCACAAACGGCGTCGTTACCGCCAAAATGCTTTCAAGCGGCGTAAACAAAGA 655

allele 16 CGTTACGGTCACAAACGGCGTCGTTACCGCCAAAATGCTTTCAAGCGGCGTAAACAAAGA 655

allele 26 CGTTACGGTCGCAAACGGCGTCGTTACCGCCGAAATGCTTTCAAGCGGCGTAAACAAAGA 657

allele 12 AGTTGAAGTCGCAAAAGGCGTCGTTACCGCCGAAATGAAACCAAGCGGCGTAAACAAAGA 658

allele 23 AGTTGAAGTCGCAAAAGGCGTCGTTACCGCCGAAATGAAACCAAGCGGCGTAAACAAAGA 658

allele 15 AGTTAAAGTCGAAAACGGCGTCGTCACCGCCCAAATGGCTTCAAGCAACGTAAACAAAGA 655

allele 25 AGTTAAAGTCGAAAACGGCGTCGTCACCGCCCAAATGGCTTCAACCGGCGTAAACAATGA 655

allele 10 AGTTAAAGTCGAAAACGGCGTCGTCACCGCCCAAATGGCTTCAACCGGCGTAAACAATGA 658

allele 2 AGTTGAAGTCGCAAAAGGCGTCGTTACCGCCCAAATGGCTTCAACCGGCGTAAACAAAGA 655

*** *** *** ******** ****** ***** ** * ********* **

allele 3 AATCAAAGGCAAAAAACTCTCCCTGTGGGCCAAGCGTGAAGACGGTTCGGTAAAATGGTT 717

allele 14 AATCAAAGGCAAAAAACTCTCCCTGTGGGCCAAGCGTGAAGACGGTTCGGTAAAATGGTT 717

allele 8 AATCAAAGGCAAAAAACTCTCCCTGTGGGCCAAGCGTGAAGACGGTTCGGTAAAATGGTT 717

allele 19 AATCAAAGGCAAAAAACTCTCCCTGTGGGCCAAGCGTGAAGACGGTTCGGTAAAATGGTT 717

allele 11 AATCAAAGGCAAAAAACTCTCCCTGTGGGCCAAGCGTGAAGACGGTTCGGTAAAATGGTT 717

allele 21 AATCAAAGGCAAAAAACTCTCCCTGTGGGCCAAGCGTGAAGACGGTTCGGTAAAATGGTT 717

allele 17 AATCAAAGGCAAAAAACTCTCCCTGTGGGCCAAGCGTGAAGACGGTTCGGTAAAATGGTT 717

allele 6 AATCAAAGGCAAAAAACTCTCCCTGTGGGCCAAGCGTGAAGACGGTTCGGTAAAATGGTT 717

allele 29 AATCAAAGGCAAAAAACTCTCCCTGTGGGCCAAGCGTGAAGACGGTTCGGTAAAATGGTT 717

allele 1 AATCAAAGGCAAAAAACTCTCCCTGTGGGCCAAGCGTGAAGACGGTTCGGTAAAATGGTT 717

allele 27 AATCAAAGGCAAAAAACTCTCCCTGTGGGCCAAGCGTGAAGACGGTTCGGTAAAATGGTT 717

allele 9 AATCAAAGGCAAAAAACTCTCCCTGTGGGCCAAGCGTGAAGACGGTTCGGTAAAATGGTT 717

allele 22 AATCCAAGGCAAAAGACTCTCCCTGTGGGCCAGGCGTGAAGCCGGTTCGGTAAAATGGTT 717

allele 13 AATCAAAGGCAAAAAACTCTCCCTGTGGGGCAGGCGTGAAAACGGTTCGGTAAAATGGTT 717

allele 24 AATCAAAGGCAAAAAACTCTCCCTGTGGGCCAAGCGTGAAGACGGTTCGGTAAAATGGTT 717

allele 18 AATCAAAGGCAAAAAACTCTCCCTGTGGGCCAAGCGTGAAGACGGTTCGGTAAAATGGTT 715

allele 20 AATCAAAGGCAAAAAACTCTCCCTGTGGGCCAAGCGTGAAGACGGTTCGGTAAAATGGTT 718

allele 7 AATCAAAGACAAAAAACTCTCCCTGTGGGCCAAGCGTGAAAACGGTTCGGTAAAATGGTT 717

allele 4 AATCAAAGGCAAAAAACTCTCCCTGTGGGCCAAGCGTGAAGACGGTTCGGTAAAATGGTT 717

allele 28 AATCCAAGGCAAAAAACTCTCCCTGTGGGCCAAGCGTGAAGACGGTTCGGTAAAATGGTT 717

allele 5 AATCCAAGGCAAAAGACTCTCCCTGTGGGCCAAGCGTGAAGCCGGTTCGGTAAAATGGTT 715

allele 16 AATCAAAGGCAAAAAACTCTCCCTGTGGGCCAAGCGTGAAGACGGTTCGGTAAAATGGTT 715

allele 26 AATCCAAGGCAAAAGACTCTCCCTGTGGGCCAAGCGTGAAGACGGTTCGGTAAAATGGTT 717

allele 12 AATCAAAGGCAAAAAACTCTCCCTGTGGGCCAAGCGTGAAGACGGTTCGGTAAAATGGTT 718

allele 23 AATCAAAGGCAAAAAACTCTCCCTGTGGGCCAAGCGTGAAGACGGTTCGGTAAAATGGTT 718

allele 15 AATCAAAGACAAAAAACTCTCCCTGTGGGCCAAGCGTGAAAACGGTTCGGTAAAATGGTT 715

allele 25 AATCAAAGGCAAAAAACTCTCCCTGTGGGCCAAGCGTGAAGACGGTTCGGTAAAATGGTT 715

allele 10 AATCAAAGGCAAAAAACTCTCCCTGTGGGCCAAGCGTCAAGACGGTTCGGTAAAATGGTT 718

allele 2 AATCCAAGGCAAAAAACTCTCCCTGTGGGCCAAGCGTCAAGACGGTTCGGTAAAATGGTT 715

**** *** ***** ************** ** **** ** ******************

allele 3 CTGCGGACAGCCGGTTAAGCGCGACGCCGGCGCCAAAACCGGCGCCGACGA--------- 768

allele 14 CTGCGGACAGCCGGTTAAGCG---CACCGGCGACAA---------CGACGA--------- 756

allele 8 CTGCGGACAGCCGGTTACGCG---CGCCAAAGCCAAA---GACGCCGACGACG------T 765

allele 19 CTGCGGACAGCCGGTTAAGCG---CACCGAAGCCAA------CGCCGACG---------- 758

allele 11 CTGCGGACAGCCGGTTAAGCG---CGAC---GCCAAC---AACGCCAACAACGACGCCGT 768

allele 21 CTGCGGACAGCCGGTTAAGCG---CGCC---GACAAC---AACGGCAACA---------T 759

allele 17 CTGCGGACAGCCGGTTAAGCGCGACGCCGGCGCCAAAACCGGCGCCGACGACG------- 770

allele 6 CTGCGGACAGCCGGTTACGCG---CAACGACGCCAAA------GCCGACGA--------- 759

allele 29 CTGCGGACAGCCGGTTACGCG---CAACGACGCCAAA------GCCGA------------ 756

allele 1 CTGCGGACAGCCGGTTAAGCGCGACGCCGGCGCCAAA------GCCGACGA--------- 762

allele 27 CTGCGGACAGCCGGTTAAGCGCGACGCCGGCGCCAAA------GCCGACGA--------- 762

allele 9 CTGCGGACAGCCGGTTAAGCGCGACGCCGGCGCCAAA------GCCGACGA--------- 762

allele 22 CTGCGGACAGCCGGTTAAGCGCGACGCCGGCGCCAAA------GCCGACGA--------- 762

allele 13 CTGCGGACAGCCGGTTAAGCGCGACGCCGGCGCCAAA------GCCGACGA--------- 762

allele 24 CTGCGGACAGCCGGTTAAGCGCGACGCCGGCGCCAAA------GCCGACGA--------- 762

allele 18 CTGCGGACAGCCGGTTAAGCGCGACGCCGGCGCCAAA------GCCGACGA--------- 760

allele 20 CTGCGGACAGCCGGTTAAGCGCGACGCCGGCGCCAAA------GCCGACGA--------- 763

allele 7 CTGCGGACAGCCGGTTAAGCGCGACGCCGGCGCCAAA------GCCGACGA--------- 762

allele 4 CTGCGGACAGCCGGTTAAGCGCGACGCCGGCGCCAAA------GCCGACGA--------- 762

allele 28 CTGCGGACAGCCGGTTAAGCGCGACGCCGGCGCCAAA------GCCGACGA--------- 762

allele 5 CTGCGGACAGCCGGTTAAGCGCGACGCCGGCGCCAAA------GCCGACGA--------- 760

allele 16 CTGCGGACAGCCGGTTAAGCGCGACGCCGGCGCCAAA------GCCGACGA--------- 760

allele 26 CTGCGGACAGCCGGTTAAGCGCGACGCCGGCGCCAAA------GCCGACGA--------- 762

allele 12 CTGCGGACAGCCGGTTAAGCGCGACGCCGGCGCCAAA------GCCGACGA--------- 763

allele 23 CTGCGGACAGCCGGTTAAGCGCGACGCCGGCGCCAAA------GCCGACGA--------- 763

allele 15 CTGCGGACAGCCGGTTAAGCGCGACGCCGGCGCCAAA------GCCGACGA--------- 760

allele 25 CTGCGGACAGCCGGTTAAGCGCGACGCCGGCGCCAAA------GCCGACGA--------- 760

allele 10 CTGCGGACAGCCGGTTAAGCGCGACGCCGGCGCCAAA------GCCGACGA--------- 763

allele 2 CTGCGGACAGCCGGTTAAGCGCGACGCCGGCGCCAAA------GCCGACGA--------- 760

***************** *** * * * *** * *

allele 3 ---CGTCAAAGCCGACGGCAACAACGGC------------ATCAACACCAAGCACCTGCC 813

allele 14 ---CACCGTTGCCGACGCCAACAACGCC------------ATCGACACCAAGCACCTGCC 801

allele 8 TACCGACGACGCCGGCACCGACAACGGCGGCAAAGGCAAAATCGACACCAAGCACCTGCC 825

allele 19 --CCGCCGGCAAAGACACCACCAACGGC------------ATCAACACCAAGCACCTGCC 804

allele 11 CACCGACGACAC---CACCGGCAACGGCAAC---GAAAAAATCGAAACCAAGCACCTGCC 822

allele 21 TACCG------------CCGACAACGGCAAC---GCCA---TCGAAACCAAGCACCTGCC 801

allele 17 --TCAAAGCCGACGGCAAAGACACCGAC---------AAAATCAACACCAAGCACCTGCC 819

allele 6 ---CGTCAAAGCCGACGCCGCCAACGCC------------ATCGAAACCAAGCACCTGCC 804

allele 29 ---CGCCAAAGACGACACCGTCACCGCC------------ATCGAAACCAAGCACCTGCC 801

allele 1 ---CGTCAAAGCCGACGCCGCCAACGCC------------ATCGAAACCAAGCACCTGCC 807

allele 27 ---CGTCAAAGCCGACGCCGCCAACGCC------------ATCGAAACCAAGCACCTGCC 807

allele 9 ---CGTCAAAGCCGACGCCGCCAACGCC------------ATCGAAACCAAGCACCTGCC 807

allele 22 ---CGTCAAAGCCGACGCCGCCAACGCC------------ATCGAAACCAAGCACCTGCC 807

allele 13 ---CGTCAAAGCCGACGCCGCCAACGCC------------ATCGAAACCAAGCACCTGCC 807

allele 24 ---CGTCAAAGCCGACGCCGCCAACGCC------------ATCGAAACCAAGCACCTGCC 807

allele 18 ---CGTCAAAGCCGACGCCGCCAACGCC------------ATCGAAACCAAGCACCTGCC 805

allele 20 ---CGTCAAAGCCGACGCCGCCAACGCC------------ATCGAAACCAAGCACCTGCC 808

allele 7 ---CGTCAAAGCCGACGCCGCCAACGCC------------ATCGAAACCAAGCACCTGCC 807

allele 4 ---CGTCAAAGCCGACGCCGCCAACGCC------------ATCGAAACCAAGCACCTGCC 807

allele 28 ---CGTCAAAGCCGACGCCGCCAACGCC------------ATCGAAACCAAGCACCTGCC 807

allele 5 ---CGTCAAAGCCGACGCCGCCAACGCC------------ATCGAAACCAAGCACCTGCC 805

allele 16 ---CGTCAAAGCCGACGCCGCCAACGCC------------ATCGAAACCAAGCACCTGCC 805

allele 26 ---CGTCAAAGCCGACGCCGCCAACGCC------------ATCGAAACCAAGCACCTGCC 807

allele 12 ---CGTCAAAGCCGACGCCGCCAACGCC------------ATCGAAACCAAGCACCTGCC 808

allele 23 ---CGTCAAAGCCGACGCCGCCAACGCC------------ATCGAAACCAAGCACCTGCC 808

allele 15 ---CGTCAAAGCCGACGCCGCCAACGCC------------ATCGAAACCAAGCACCTGCC 805

allele 25 ---CGTCAAAGCCGACGCCGCCAACGCC------------ATCGAAACCAAGCACCTGCC 805

allele 10 ---CGTCAAAGCCGACGCCGCCAACGCC------------ATCGAAACCAAGCACCTGCC 808

allele 2 ---CGTCAAAGCCGACGCCGCCAACGCC------------ATCGAAACCAAGCACCTGCC 805

* ** ** * ** * **************

allele 3 GTCAACCTGCCGCGATAAACATGATGCCAAATGAGGCAAATTAGGCCTTAAATTTTAAAT 873

allele 14 GTCAACCTGCCGCGATAAACATGATGCCAAATGAGGCAAATTAGGCCTTAAATTTTAAAT 861

allele 8 GTCAACCTGCCGCGATAAATCAACTGCCAAATAAGGCAAATTAGGCCTTAAATTTTAAAT 885

allele 19 GTCAACCTGCCGCGATGAATCATCTGCCACCTAAGGCAAATTAGGCCTTAAATTTTAAAT 864

allele 11 GTCAACCTGCCGCGATGAATCATCTGCCACCTAAGGCAAATTAGGCCTTAAATTTTAAAT 882

allele 21 GTCAACCTGCCGCGATGAATCATCTGCCACCTAAGGCAAATTAGGCCTTAAATTTTAAAT 861

allele 17 GTCAACCTGCCGCGATGAATCATCTGCCACCTAAGGCAAATTAGGCCTTAAATTTTAAAT 879

allele 6 GTCAACCTGCCGCGATGAATCATCTGCCACCTAAGGCAAATTAGGCCTTAAATTTTAAAT 864

allele 29 GTCAACCTGCCGCGATGAATCATCTGCCACCTAAGGCAAATTAGGCCTTAAATTTTAAAT 861

allele 1 GTCAACCTGCCGCGATGAATCATCTGCCACCTAAGGCAAATTAGGCCTTAAATTTTAAAT 867

allele 27 GTCAACCTGCCGCGATGAACCAACTGCCACCTAAGGCAAATTAGGCCTTAAATTTTAAAT 867

allele 9 GTCAACCTGCCGCGATGAATCATCTGCCACCTAAGGCAAATTAGGCCTTAAATTTTAAAT 867

allele 22 GTCAACCTGCCGCGATGAATCATCTGCCACCTAAGGCAAATTAGGCCTTAAATTTTAAAT 867

allele 13 GTCAACCTGCCGCGATGAATCATCTGCCACCTAAGGCAAATTAGGCCTTAAATTTTAAAT 867

allele 24 GTCAACCTGCCGCGATGAATCATCTGCCACCTAAGGCAAATTAGGCCTTAAATTTTAAAT 867

allele 18 GTCAACCTGCCGCGATGAATCATCTGCCACCTAAGGCAAATTAGGCCTTAAATTTTAAAT 865

allele 20 GTCAACCTGCCGCGATGAATCATCTGCCACCTAAGGCAAATTAGGCCTTAAATTTTAAAT 868

allele 7 GTCAACCTGCCGCGATGAATCATCTGCCACCTAAGGCAAATTAGGCCTTAAATTTTAAAT 867

allele 4 GTCAACCTGCCGCGATGAATCATCTGCCACCTAAGGCAAATTAGGCCTTAAATTTTAAAT 867

allele 28 GTCAACCTGCCGCGATGAATCATCTGCCACCTAAGGCAAATTAGGCCTTAAATTTTAAAT 867

allele 5 GTCAACCTGCCGCGATGAATCATCTGCCACCTAAGGCAAATTAGGCCTTAAATTTTAAAT 865

allele 16 GTCAACCTGCCGCGATGAATCATCTGCCACCTAAGGCAAATTAGGCCTTAAATTTTAAAT 865

allele 26 GTCAACCTGCCGCGATGAATCATCTGCCACCTAAGGCAAATTAGGCCTTAAATTTTAAAT 867

allele 12 GTCAACCTGCCGCGATGAATCATCTGCCACCTAAGGCAAATTAGGCCTTAAATTTTAAAT 868

allele 23 GTCAACCTGCCGCGATGAATCATCTGCCACCTAAGGCAAATTAGGCCTTAAATTTTAAAT 868

allele 15 GTCAACCTGCCGCGATGAATCATCTGCCACCTAAGGCAAATTAGGCCTTAAATTTTAAAT 865

allele 25 GTCAACCTGCCGCGATGAATCATCTGCCACCTAAGGCAAATTAGGCCTTAAATTTTAAAT 865

allele 10 GTCAACCTGCCGCGATGAATCATCTGCCACCTAAGGCAAATTAGGCCTTAAATTTTAAAT 868

allele 2 GTCAACCTGCCGCGATGAATCATCTGCCACCTAAGGCAAATTAGGCCTTAAATTTTAAAT 865

**************** ** ***** * ***************************

allele 3 AAATCAAGCGGTAAGTGATTTCCCACGGCCGCCCGGATCAACCCGGGCGGCTTGTCTTTT 933

allele 14 AAATCAAGCGGTAAGTGATTTCCCACGGCCGCCCGGATCAACCCGGGCGGCTTGTCTTTT 921

allele 8 AAATCAAACGGTAAGTGATTTCCCACGGCCGCCCGGATCAACCCGGGCGGCTTGTCTTTT 945

allele 19 AAATCAAGCGGTAAGTGATTTCCCACGGCCGCCCGGATCAACCCGGGCGGCTTGTCTTTT 924

allele 11 AAATCAAGCGGTAAGTGATTTCCCACGGCCGCCCGGATCAACCCGGGCGGCTTGTCTTTT 942

allele 21 AAATCAAGCGGTAAGTGATTTCCCACGGCCGCCCGGATCAACCCGGGCGGCTTGTCTTTT 921

allele 17 AAATCAAGCGGTAAGTGATTTCCCACGGCCGCCCGGATCAACCCGGGCGGCTTGTCTTTT 939

allele 6 AAATCAAGCGGTAAGTGATTTCCCACGGCCGCCCGGATCAACCCGGGCGGCTTGTCTTTT 924

allele 29 AAATCAAGCGGTAAGTGATTTCCCACGGCCGCCCGGATCAACCCGGGCGGCTTGTCTTTT 921

allele 1 AAATCAAGCGGTAAGTGATTTCCCACGGCCGCCCGGATCAACCCGGGCGGCTTGTCTTTT 927

allele 27 AAATCAAGCGGTAAGTGATTTCCCACGGCCGCCCGGATCAACCCGGGCGGCTTGTCTTTT 927

allele 9 AAATCAAGCGGTAAGTGATTTCCCACGGCCGCCCGGATCAACCCGGGCGGCTTGTCTTTT 927

allele 22 AAATCAAGCGGTAAGTGATTTCCCACGGCCGCCCGGATCAACCCGGGCGGCTTGTCTTTT 927

allele 13 AAATCAAGCGGTAAGTGATTTCCCACGGCCGCCCGGATCAACCCGGGCGGCTTGTCTTTT 927

allele 24 AAATCAAGCGGTAAGTGATTTCCCACGGCCGCCCGGATCAACCCGGGCGGCTTGTCTTTT 927

allele 18 AAATCAAGCGGTAAGTGATTTCCCACGGCCGCCCGGATCAACCCGGGCGGCTTGTCTTTT 925

allele 20 AAATCAAGCGGTAAGTGATTTCCCACGGCCGCCCGGATCAACCCGGGCGGCTTGTCTTTT 928

allele 7 AAATCAAGCGGTAAGTGATTTCCCACGGCCGCCCGGATCAACCCGGGCGGCTTGTCTTTT 927

allele 4 AAATCAAGCGGTAAGTGATTTCCCACGGCCGCCCGGATCAACCCGGGCGGCTTGTCTTTT 927

allele 28 AAATCAAGCGGTAAGTGATTTCCCACGGCCGCCCGGATCAACCCGGGCGGCTTGTCTTTT 927

allele 5 AAATCAAGCGGTAAGTGATTTCCCACGGCCGCCCGGATCAACCCGGGCGGCTTGTCTTTT 925

allele 16 AAATCAAGCGGTAAGTGATTTCCCACGGCCGCCCGGATCAACCCGGGCGGCTTGTCTTTT 925

allele 26 AAATCAAGCGGTAAGTGATTTCCCACGGCCGCCCGGATCAACCCGGGCGGCTTGTCTTTT 927

allele 12 AAATCAAGCGGTAAGTGATTTCCCACGGCCGCCCGGATCAACCCGGGCGGCTTGTCTTTT 928

allele 23 AAATCAAGCGGTAAGTGATTTCCCACGGCCGCCCGGATCAACCCGGGCGGCTTGTCTTTT 928

allele 15 AAATCAAGCGGTAAGTGATTTCCCACGGCCGCCCGGATCAACCCGGGCGGCTTGTCTTTT 925

allele 25 AAATCAAGCGGTAAGTGATTTCCCACGGCCGCCCGGATCAACCCGGGCGGCTTGTCTTTT 925

allele 10 AAATCAAGCGGTAAGTGATTTCCCACGGCCGCCCGGATCAACCCGGGCGGCTTGTCTTTT 928

allele 2 AAATCAAGCGGTAAGTGATTTCCCACGGCCGCCCGGATCAACCCGGGCGGCTTGTCTTTT 925

******* ****************************************************

allele 3 AAGGGTTTGCAAGGCGGGCGGGGTCGTCCGTTCCGGTGGAAATAATATATCGATTGCGCT 993

allele 14 AAGGGTTTGCAAGGCGGGCGGGGTCGTCCGTTCCGGTGGAAATAATATATCGATTGCGCT 981

allele 8 AAGGGTTTGCAAGGCGGGCGGGGTCGTCCGTTCCGGTGGAAATAATATATCGATTGCGCT 1005

allele 19 AAGGGTTTGCAAGGCGGGCGGGGTCGTCCGTTCCGGTGGAAATAATATATCGATTGCGCT 984

allele 11 AAGGGTTTGCAAGGCGGGCGGGGTCGTCCGTTCCGGTGGAAATAATATATCGATTGCGCT 1002

allele 21 AAGGGTTTGCAAGGCGGGCGGGGTCGTCCGTTCCGGTGGAAATAATATATCGATTGCGCT 981

allele 17 AAGGGTTTGCAAGGCGGGCGGGGTCGTCCGTTCCGGTGGAAATAATATATCGATTGCGCT 999

allele 6 AAGGGTTTGCAAGGCGGGCGGGGTCGTCCGTTCCGGTGGAAATAATATATCGATTGCGCT 984

allele 29 AAGGGTTTGCAAGGCGGGCGGGGTCGTCCGTTCCGGTGGAAATAATATATCGATTGCGCT 981

allele 1 AAGGGTTTGCAAGGCGGGCGGGGTCGTCCGTTCCGGTGGAAATAATATATCGATTGCGCT 987

allele 27 AAGGGTTTGCAAGGCGGGCGGGGTCGTCCGTTCCGGTGGAAATAATATATCGATTGCGCT 987

allele 9 AAGGGTTTGCAAGGCGGGCGGGGTCGTCCGTTCCGGTGGAAATAATATATCGATTGCGCT 987

allele 22 AAGGGTTTGCAAGGCGGGCGGGGTCGTCCGTTCCGGTGGAAATAATATATCGATTGCGCT 987

allele 13 AAGGGTTTGCAAGGCGGGCGGGGTCGTCCGTTCCGGTGGAAATAATATATCGATTGCGCT 987

allele 24 AAGGGTTTGCAAGGCGGGCGGGGTCGTCCGTTCCGGTGGAAATAATATATCGATTGCGCT 987

allele 18 AAGGGTTTGCAAGGCGGGCGGGGTCGTCCGTTCCGGTGGAAATAATATATCGATTGCGCT 985

allele 20 AAGGGTTTGCAAGGCGGGCGGGGTCGTCCGTTCCGGTGGAAATAATATATCGATTGCGCT 988

allele 7 AAGGGTTTGCAAGGCGGGCGGGGTCGTCCGTTCCGGTGGAAATAATATATCGATTGCGCT 987

allele 4 AAGGGTTTGCAAGGCGGGCGGGGTCGTCCGTTCCGGTGGAAATAATATATCGATTGCGCT 987

allele 28 AAGGGTTTGCAAGGCGGGCGGGGTCGTCCGTTCCGGTGGAAATAATATATCGATTGCGCT 987

allele 5 AAGGGTTTGCAAGGCGGGCGGGGTCGTCCGTTCCGGTGGAAATAATATATCGATTGCGCT 985

allele 16 AAGGGTTTGCAAGGCGGGCGGGGTCGTCCGTTCCGGTGGAAATAATATATCGATTGCGCT 985

allele 26 AAGGGTTTGCAAGGCGGGCGGGGTCGTCCGTTCCGGTGGAAATAATATATCGATTGCGCT 987

allele 12 AAGGGTTTGCAAGGCGGGCGGGGTCGTCCGTTCCGGTGGAAATAATATATCGATTGCGCT 988

allele 23 AAGGGTTTGCAAGGCGGGCGGGGTCGTCCGTTCCGGTGGAAATAATATATCGATTGCGCT 988

allele 15 AAGGGTTTGCAAGGCGGGCGGGGTCGTCCGTTCCGGTGGAAATAATATATCGATTGCGCT 985

allele 25 AAGGGTTTGCAAGGCGGGCGGGGTCGTCCGTTCCGGTGGAAATAATATATCGATTGCGCT 985

allele 10 AAGGGTTTGCAAGGCGGGCGGGGTCGTCCGTTCCGGTGGAAATAATATATCGATTGCGCT 988

allele 2 AAGGGTTTGCAAGGCGGGCGGGGTCGTCCGTTCCGGTGGAAATAATATATCGATTGCGCT 985

************************************************************

allele 3 TCAAGGCCCTGCATGTGCCTCATTGCCACCCGTTTAAACACGGTTTTTATCTGACAGGCG 1053

allele 14 TCAAGGCCCTGCATGTGCCTCATTGCCACCCGTTTAAACACGGTTTTTATCTGACAGGCG 1041

allele 8 TCAAGGCCCTGCATGTGCCTCATTGCCACCCGTTTAAACACGGTTTTTATCTGACAGGCG 1065

allele 19 TCAAGGCCCTGCATGTGCCTCATTGCCACCCGTTTAAACACGGTTTTTATCTGACAGGCG 1044

allele 11 TCAAGGCCCTGCATGTGCCTCATTGCCACCCGTTTAAACACGGTTTTTATCTGACAGGCG 1062

allele 21 TCAAGGCCCTGCATGTGCCTCATTGCCACCCGTTTAAACACGGTTTTTATCTGACAGGCG 1041

allele 17 TCAAGGCCCTGCATGTGCCTCATTGCCACCCGTTTAAACACGGTTTTTATCTGACAGGCG 1059

allele 6 TCAAGGCCCTGCATGTGCCTCATTGCCACCCGTTTAAACACGGTTTTTATCTGACAGGCG 1044

allele 29 TCAAGGCCCTGCATGTGCCTCATTGCCACCCGTTTAAACACGGTTTTTATCTGACAGGCG 1041

allele 1 TCAAGGCCCTGCATGTGCCTCATTGCCACCCGTTTAAACACGGTTTTTATCTGACAGGCG 1047

allele 27 TCAAGGCCCTGCATGTGCCTCATTGCCACCCGTTTAAACACGGTTTTTATCTGACAGGCG 1047

allele 9 TCAAGGCCCTGCATGTGCCTCATTGCCACCCGTTTAAACACGGTTTTTATCTGACAGGCG 1047

allele 22 TCAAGGCCCTGCATGTGCCTCATTGCCACCCGTTTAAACACGGTTTTTATCTGACAGGCG 1047

allele 13 TCAAGGCCCTGCATGTGCCTCATTGCCACCCGTTTAAACACGGTTTTTATCTGACAGGCG 1047

allele 24 TCAAGGCCCTGCATGTGCCTCATTGCCACCCGTTTAAACACGGTTTTTATCTGACAGGCG 1047

allele 18 TCAAGGCCCTGCATGTGCCTCATTGCCACCCGTTTAAACACGGTTTTTATCTGACAGGCG 1045

allele 20 TCAAGGCCCTGCATGTGCCTCATTGCCACCCGTTTAAACACGGTTTTTATCTGACAGGCG 1048

allele 7 TCAAGGCCCTGCATGTGCCTCATTGCCACCCGTTTAAACACGGTTTTTATCTGACAGGCG 1047

allele 4 TCAAGGCCCTGCATGTGCCTCATTGCCACCCGTTTAAACACGGTTTTTATCTGACAGGCG 1047

allele 28 TCAAGGCCCTGCATGTGCCTCATTGCCACCCGTTTAAACACGGTTTTTATCTGACAGGCG 1047

allele 5 TCAAGGCCCTGCATGTGCCTCATTGCCACCCGTTTAAACACGGTTTTTATCTGACAGGCG 1045

allele 16 TCAAGGCCCTGCATGTGCCTCATTGCCACCCGTTTAAACACGGTTTTTATCTGACAGGCG 1045

allele 26 TCAAGGCCCTGCATGTGCCTCATTGCCACCCGTTTAAACACGGTTTTTATCTGACAGGCG 1047

allele 12 TCAAGGCCCTGCATGTGCCTCATTGCCACCCGTTTAAACACGGTTTTTATCTGACAGGCG 1048

allele 23 TCAAGGCCCTGCATGTGCCTCATTGCCACCCGTTTAAACACGGTTTTTATCTGACAGGCG 1048

allele 15 TCAAGGCCCTGCATGTGCCTCATTGCCACCCGTTTAAACACGGTTTTTATCTGACAGGCG 1045

allele 25 TCAAGGCCCTGCATGTGCCTCATTGCCACCCGTTTAAACACGGTTTTTATCTGACAGGCG 1045

allele 10 TCAAGGCCCTGCATGTGCCTCATTGCCACCCGTTTAAACACGGTTTTTATCTGACAGGCG 1048

allele 2 TCAAGGCCCTGCATGTGCCTCATTGCCACCCGTTTAAACACGGTTTTTATCTGACAGGCG 1045

************************************************************

allele 3 CGCAATCCGCCCCCTCATTTGTTAATCCGCCATATTGTATTGAAACACCGCCCGGAACCC 1113

allele 14 CGCAATCCGCCCCCTCATTTGTTAATCCGCCATATTGTATTGAAACACCGCCCGGAACCC 1101

allele 8 CGCAATCCGCCCCCTCATTTGTTAATCCGCCATATTGTATTGAAACACCGCCCGGAACCC 1125

allele 19 CGCAATCCGCCCCCTCATTTGTTAATCCGCCATATTGTATTGAAACACCGCCCGGAACCC 1104

allele 11 CGCAATCCGCCCCCTCATTTGTTAATCCGCCATATTGTATTGAAACACCGCCCGGAACCC 1122

allele 21 CGCAATCCGCCCCCTCATTTGTTAATCCGCCATATTGTATTGAAACACCGCCCGGAACCC 1101

allele 17 CGCAATCCGCCCCCTCATTTGTTAATCCGCCATATTGTATTGAAACACCGCCCGGAACCC 1119

allele 6 CGCAATCCGCCCCCTCATTTGTTAATCCGCCATATTGTATTGAAACACCGCCCGGAACCC 1104

allele 29 CGCAATCCGCCCCCTCATTTGTTAATCCGCCATATTGTATTGAAACACCGCCCGGAACCC 1101

allele 1 CGCAATCCGCCCCCTCATTTGTTAATCCGCCATATTGTATTGAAACACCGCCCGGAACCC 1107

allele 27 CGCAATCCGCCCCCTCATTTGTTAATCCGCCATATTGTATTGAAACACCGCCCGGAACCC 1107

allele 9 CGCAATCCGCCCCCTCATTTGTTAATCCGCCATATTGTATTGAAACACCGCCCGGAACCC 1107

allele 22 CGCAATCCGCCCCCTCATTTGTTAATCCGCCATATTGTATTGAAACACCGCCCGGAACCC 1107

allele 13 CGCAATCCGCCCCCTCATTTGTTAATCCGCCATATTGTATTGAAACACCGCCCGGAACCC 1107

allele 24 CGCAATCCGCCCCCTCATTTGTTAATCCGCCATATTGTATTGAAACACCGCCCGGAACCC 1107

allele 18 CGCAATCCGCCCCCTCATTTGTTAATCCGCCATATTGTATTGAAACACCGCCCGGAACCC 1105

allele 20 CGCAATCCGCCCCCTCATTTGTTAATCCGCCATATTGTATTGAAACACCGCCCGGAACCC 1108

allele 7 CGCAATCCGCCCCCTCATTTGTTAATCCGCCATATTGTATTGAAACACCGCCCGGAACCC 1107

allele 4 CGCAATCCGCCCCCTCATTTGTTAATCCGCCATATTGTATTGAAACACCGCCCGGAACCC 1107

allele 28 CGCAATCCGCCCCCTCATTTGTTAATCCGCCATATTGTATTGAAACACCGCCCGGAACCC 1107

allele 5 CGCAATCCGCCCCCTCATTTGTTAATCCGCCATATTGTATTGAAACACCGCCCGGAACCC 1105

allele 16 CGCAATCCGCCCCCTCATTTGTTAATCCGCCATATTGTATTGAAACACCGCCCGGAACCC 1105

allele 26 CGCAATCCGCCCCCTCATTTGTTAATCCGCCATATTGTATTGAAACACCGCCCGGAACCC 1107

allele 12 CGCAATCCGCCCCCTCATTTGTTAATCCGCCATATTGTATTGAAACACCGCCCGGAACCC 1108

allele 23 CGCAATCCGCCCCCTCATTTGTTAATCCGCCATATTGTATTGAAACACCGCCCGGAACCC 1108

allele 15 CGCAATCCGCCCCCTCATTTGTTAATCCGCCATATTGTATTGAAACACCGCCCGGAACCC 1105

allele 25 CGCAATCCGCCCCCTCATTTGTTAATCCGCCATATTGTATTGAAACACCGCCCGGAACCC 1105

allele 10 CGCAATCCGCCCCCTCATTTGTTAATCCGCCATATTGTATTGAAACACCGCCCGGAACCC 1108

allele 2 CGCAATCCGCCCCCTCATTTGTTAATCCGCCATATTGTATTGAAACACCGCCCGGAACCC 1105

************************************************************

allele 3 GATATAATCCGCCCTTCAACATCAGTGAAAATCTTTTTTTAACCGGTTAAACCGAATAAG 1173

allele 14 GATATAATCCGCCCTTCAACATCAGTGAAAATCTTTTTTTAACCGGTTAAACCGAATAAG 1161

allele 8 GATATAATCCGCCCTTCAACATCAGTGAAAATCTTTTTTTAACCGGTTAAACCGAATAAG 1185

allele 19 GATATAATCCGCCCTTCAACATCAGTGAAAATCTTTTTTTAACCGGTTAAACCGAATAAG 1164

allele 11 GATATAATCCGCCCTTCAACATCAGTGAAAATCTTTTTTTAACCGGTTAAACCGAATAAG 1182

allele 21 GATATAATCCGCCCTTCAACATCAGTGAAAATCTTTTTTTAACCGGTTAAACCGAATAAG 1161

allele 17 GATATAATCCGCCCTTCAACATCAGTGAAAATCTTTTTTTAACCGGTTAAACCGAATAAG 1179

allele 6 GATATAATCCGCCCTTCAACATCAGTGAAAATCTTTTTTTAACCGGTTAAACCGAATAAG 1164

allele 29 GATATAATCCGCCCTTCAACATCAGTGAAAATCTTTTTTTAACCGGTTAAACCGAATAAG 1161

allele 1 GATATAATCCGCCCTTCAACATCAGTGAAAATCTTTTTTTAACCGGTTAAACCGAATAAG 1167

allele 27 GATATAATCCGCCCTTCAACATCAGTGAAAATCTTTTTTTAACCGGTTAAACCGAATAAG 1167

allele 9 GATATAATCCGCCCTTCAACATCAGTGAAAATCTTTTTTTAACCGGTTAAACCGAATAAG 1167

allele 22 GATATAATCCGCCCTTCAACATCAGTGAAAATCTTTTTTTAACCGGTTAAACCGAATAAG 1167

allele 13 GATATAATCCGCCCTTCAACATCAGTGAAAATCTTTTTTTAACCGGTTAAACCGAATAAG 1167

allele 24 GATATAATCCGCCCTTCAACATCAGTGAAAATCTTTTTTTAACCGGTTAAACCGAATAAG 1167

allele 18 GATATAATCCGCCCTTCAACATCAGTGAAAATCTTTTTTTAACCGGTTAAACCGAATAAG 1165

allele 20 GATATAATCCGCCCTTCAACATCAGTGAAAATCTTTTTTTAACCGGTTAAACCGAATAAG 1168

allele 7 GATATAATCCGCCCTTCAACATCAGTGAAAATCTTTTTTTAACCGGTTAAACCGAATAAG 1167

allele 4 GATATAATCCGCCCTTCAACATCAGTGAAAATCTTTTTTTAACCGGTTAAACCGAATAAG 1167

allele 28 GATATAATCCGCCCTTCAACATCAGTGAAAATCTTTTTTTAACCGGTTAAACCGAATAAG 1167

allele 5 GATATAATCCGCCCTTCAACATCAGTGAAAATCTTTTTTTAACCGGTTAAACCGAATAAG 1165

allele 16 GATATAATCCGCCCTTCAACATCAGTGAAAATCTTTTTTTAACCGGTTAAACCGAATAAG 1165

allele 26 GATATAATCCGCCCTTCAACATCAGTGAAAATCTTTTTTTAACCGGTTAAACCGAATAAG 1167

allele 12 GATATAATCCGCCCTTCAACATCAGTGAAAATCTTTTTTTAACCGGTTAAACCGAATAAG 1168

allele 23 GATATAATCCGCCCTTCAACATCAGTGAAAATCTTTTTTTAACCGGTTAAACCGAATAAG 1168

allele 15 GATATAATCCGCCCTTCAACATCAGTGAAAATCTTTTTTTAACCGGTTAAACCGAATAAG 1165

allele 25 GATATAATCCGCCCTTCAACATCAGTGAAAATCTTTTTTTAACCGGTTAAACCGAATAAG 1165

allele 10 GATATAATCCGCCCTTCAACATCAGTGAAAATCTTTTTTTAACCGGTTAAACCGAATAAG 1168

allele 2 GATATAATCCGCCCTTCAACATCAGTGAAAATCTTTTTTTAACCGGTTAAACCGAATAAG 1165

************************************************************

allele 3 GAGCCGAA 1181

allele 14 GAGCCGAA 1169

allele 8 GAGCCGAA 1193

allele 19 GAGCCGAA 1172

allele 11 GAGCCGAA 1190

allele 21 GAGCCGAA 1169

allele 17 GAGCCGAA 1187

allele 6 GAGCCGAA 1172

allele 29 GAGCCGAA 1169

allele 1 GAGCCGAA 1175

allele 27 GAGCCGAA 1175

allele 9 GAGCCGAA 1175

allele 22 GAGCCGAA 1175

allele 13 GAGCCGAA 1175

allele 24 GAGCCGAA 1175

allele 18 GAGCCGAA 1173

allele 20 GAGCCGAA 1176

allele 7 GAGCCGAA 1175

allele 4 GAGCCGAA 1175

allele 28 GAGCCGAA 1175

allele 5 GAGCCGAA 1173

allele 16 GAGCCGAA 1173

allele 26 GAGCCGAA 1175

allele 12 GAGCCGAA 1176

allele 23 GAGCCGAA 1176

allele 15 GAGCCGAA 1173

allele 25 GAGCCGAA 1173

allele 10 GAGCCGAA 1176

allele 2 GAGCCGAA 1173

********

**Figure S3:** Alignment of the variant sequences detected in the first experiment with *pilE* in *N. gonorrhoeae* FA1090. The allele 1 assembly is identical to the reference sequence obtained by Sanger sequencing of the amplicon. Blue text indicates sequence flanking the *pilE* gene (black text). Sequence differences are highlighted in yellow. The grey shading highlights the extent of the sequence identity between the *pilE* sequence and the various silent copies, flanking the variant sequence. Where the variant sequence was identical to part of two silent copies, the larger of the two regions of sequence identity is shown.
