# Supplementary material for: The Use of High-Throughput DNA Sequencing in the Investigation of Antigenic Variation: Application to Neisseria Species
Source: PLoS One. 2014 Jan 22;9(1):e86704. doi: 10.1371/journal.pone.0086704 (PMC3899283; doi:10.1371/journal.pone.0086704)
Supplement: Figure S4 — Alignment of the variant sequences detected in the repeat experiment with pilE in N. gonorrhoeae FA1090. The seq1 assembly is identical to the reference sequence obtained by Sanger sequencing of the amplicon. Blue text indicates sequence flanking the pilE gene (black text). Sequence differences are highlighted in yellow. The grey shading highlights the extent of the sequence identity between the pilE sequence and the various silent copies, flanking the variant sequence. (DOC) [file pone.0086704.s004.doc]

allele 1 TATTCTAACGCGTAAATTCAAAAATCTCAAATTCCGACCCAATCAACACACCCGATACCC 60

allele 3 TATTCTAACGCGTAAATTCAAAAATCTCAAATTCCGACCCAATCAACACACCCGATACCC 60

allele 4 TATTCTAACGCGTAAATTCAAAAATCTCAAATTCCGACCCAATCAACACACCCGATACCC 60

allele 31 TATTCTAACGCGTAAATTCAAAAATCTCAAATTCCGACCCAATCAACACACCCGATACCC 60

allele 30 TATTCTAACGCGTAAATTCAAAAATCTCAAATTCCGACCCAATCAACACACCCGATACCC 60

allele 32 TATTCTAACGCGTAAATTCAAAAATCTCAAATTCCGACCCAATCAACACACCCGATACCC 60

************************************************************

allele 1 CATGCCAATAAAAAAGTAACGAAAATCGGCACTAAAACTGACAATTTTCGACACTGCCGC 120

allele 3 CATGCCAATAAAAAAGTAACGAAAATCGGCACTAAAACTGACAATTTTCGACACTGCCGC 120

allele 4 CATGCCAATAAAAAAGTAACGAAAATCGGCACTAAAACTGACAATTTTCGACACTGCCGC 120

allele 31 CATGCCAATAAAAAAGTAACGAAAATCGGCACTAAAACTGACAATTTTCGACACTGCCGC 120

allele 30 CATGCCAATAAAAAAGTAACGAAAATCGGCACTAAAACTGACAATTTTCGACACTGCCGC 120

allele 32 CATGCCAATAAAAAAGTAACGAAAATCGGCACTAAAACTGACAATTTTCGACACTGCCGC 120

************************************************************

allele 1 CCCCTACTTCCGCAAACCACACCCACCTAAAAGAAAATACAAAATAAAAACAATTATATA 180

allele 3 CCCCTACTTCCGCAAACCACACCCACCTAAAAGAAAATACAAAATAAAAACAATTATATA 180

allele 4 CCCCTACTTCCGCAAACCACACCCACCTAAAAGAAAATACAAAATAAAAACAATTATATA 180

allele 31 CCCCTACTTCCGCAAACCACACCCACCTAAAAGAAAATACAAAATAAAAACAATTATATA 180

allele 30 CCCCTACTTCCGCAAACCACACCCACCTAAAAGAAAATACAAAATAAAAACAATTATATA 180

allele 32 CCCCTACTTCCGCAAACCACACCCACCTAAAAGAAAATACAAAATAAAAACAATTATATA 180

************************************************************

allele 1 GAGATAAACGCATAAAATTTCACCTCAAAACATAAAATCGGCACGAATCTTGCTTTATAA 240

allele 3 GAGATAAACGCATAAAATTTCACCTCAAAACATAAAATCGGCACGAATCTTGCTTTATAA 240

allele 4 GAGATAAACGCATAAAATTTCACCTCAAAACATAAAATCGGCACGAATCTTGCTTTATAA 240

allele 31 GAGATAAACGCATAAAATTTCACCTCAAAACATAAAATCGGCACGAATCTTGCTTTATAA 240

allele 30 GAGATAAACGCATAAAATTTCACCTCAAAACATAAAATCGGCACGAATCTTGCTTTATAA 240

allele 32 GAGATAAACGCATAAAATTTCACCTCAAAACATAAAATCGGCACGAATCTTGCTTTATAA 240

************************************************************

allele 1 TACGCAGTTGTCGCAACAAAAAACCGATGGTTAAATACATTGCATGATGCCGATGGCGTA 300

allele 3 TACGCAGTTGTCGCAACAAAAAACCGATGGTTAAATACATTGCATGATGCCGATGGCGTA 300

allele 4 TACGCAGTTGTCGCAACAAAAAACCGATGGTTAAATACATTGCATGATGCCGATGGCGTA 300

allele 31 TACGCAGTTGTCGCAACAAAAAACCGATGGTTAAATACATTGCATGATGCCGATGGCGTA 300

allele 30 TACGCAGTTGTCGCAACAAAAAACCGATGGTTAAATACATTGCATGATGCCGATGGCGTA 300

allele 32 TACGCAGTTGTCGCAACAAAAAACCGATGGTTAAATACATTGCATGATGCCGATGGCGTA 300

************************************************************

allele 1 AGCCTGAGGCATTTCCCCTTTCAATTAGGAGTAATTTTATGAATACCCTTCAAAAAGGCT 360

allele 3 AGCCTGAGGCATTTCCCCTTTCAATTAGGAGTAATTTTATGAATACCCTTCAAAAAGGCT 360

allele 4 AGCCTGAGGCATTTCCCCTTTCAATTAGGAGTAATTTTATGAATACCCTTCAAAAAGGCT 360

allele 31 AGCCTGAGGCATTTCCCCTTTCAATTAGGAGTAATTTTATGAATACCCTTCAAAAAGGCT 360

allele 30 AGCCTGAGGCATTTCCCCTTTCAATTAGGAGTAATTTTATGAATACCCTTCAAAAAGGCT 360

allele 32 AGCCTGAGGCATTTCCCCTTTCAATTAGGAGTAATTTTATGAATACCCTTCAAAAAGGCT 360

************************************************************

allele 1 TTACCCTTATCGAGCTGATGATTGTGATCGCTATCGTCGGCATTTTGGCGGCAGTCGCCC 420

allele 3 TTACCCTTATCGAGCTGATGATTGTGATCGCTATCGTCGGCATTTTGGCGGCAGTCGCCC 420

allele 4 TTACCCTTATCGAGCTGATGATTGTGATCGCTATCGTCGGCATTTTGGCGGCAGTCGCCC 420

allele 31 TTACCCTTATCGAGCTGATGATTGTGATCGCTATCGTCGGCATTTTGGCGGCAGTCGCCC 420

allele 30 TTACCCTTATCGAGCTGATGATTGTGATCGCTATCGTCGGCATTTTGGCGGCAGTCGCCC 420

allele 32 TTACCCTTATCGAGCTGATGATTGTGATCGCTATCGTCGGCATTTTGGCGGCAGTCGCCC 420

************************************************************

allele 1 TTCCCGCCTACCAAGACTACACCGCCCGCGCGCAAGTTTCCGAAGCCATCCTTTTGGCCG 480

allele 3 TTCCCGCCTACCAAGACTACACCGCCCGCGCGCAAGTTTCCGAAGCCATCCTTTTGGCCG 480

allele 4 TTCCCGCCTACCAAGACTACACCGCCCGCGCGCAAGTTTCCGAAGCCATCCTTTTGGCCG 480

allele 31 TTCCCGCCTACCAAGACTACACCGCCCGCGCGCAAGTTTCCGAAGCCATCCTTTTGGCCG 480

allele 30 TTCCCGCCTACCAAGACTACACCGCCCGCGCGCAAGTTTCCGAAGCCATCCTTTTGGCCG 480

allele 32 TTCCCGCCTACCAAGACTACACCGCCCGCGCGCAAGTTTCCGAAGCCATCCTTTTGGCCG 480

************************************************************

allele 1 AAGGTCAAAAATCAGCCGTTACCGGGTATTACCTGAATCACGGCATATGGCCGGAAGACA 540

allele 3 AAGGTCAAAAATCAGCCGTTACCGGGTATTACCTGAATCACGGCATATGGCCGGAAGACA 540

allele 4 AAGGTCAAAAATCAGCCGTTACCGGGTATTACCTGAATCACGGCATATGGCCGGAAGACA 540

allele 31 AAGGTCAAAAATCAGCCGTTACCGGGTATTACCTGAATCACGGCATATGGCCGGAAGACA 540

allele 30 AAGGTCAAAAATCAGCCGTTACCGGGTATTACCTGAATCACGGCATATGGCCGGAAGACA 540

allele 32 AAGGTCAAAAATCAGCCGTTACCGGGTATTACCTGAATCACGGCATATGGCCGGAAGACA 540

************************************************************

allele 1 ACACTTCTGCCGGCGTGGCATCCCCCCCTCCGACATCAAAGGCAAATATGTTCAAAGCGT 600

allele 3 ACACTTCTGCCGGCGTGGCATCCCCCCCTCCGACATCAAAGGCAAATATGTTCAAAGCGT 600

allele 4 ACACTTCTGCCGGCGTGGCATCCCCCCCACCGACATCAAAGGCAAATATGTTAAAAGCGT 600

allele 31 ACACTTCTGCCGGCGTGGCATCCCCC--GCCGAAATCAAAGGCAAATATGTTAAAAGCGT 598

allele 30 ACACTTCTGCCGGCGTGGCATCCCCCCCTCCGACATCAAAGGCAAATATGTTCAAAGCGT 600

allele 32 ACACTTCTGCCGGCGTGGCATCCCCCCCTCCGACATCAAAGGCAAATATGTTCAAAGCGT 600

************************** **** ****************** *******

allele 1 TACGGTCGCAAACGGCGTCGTTACCGCCGAAATGAAACCAAGCGGCGTAAACAAAGAAAT 660

allele 3 TACGGTCGCAAACGGCGTCGTTACCGCCGAAATGAAACCAAGCGGCGTAAACAAAGAAAT 660

allele 4 TACGGTCGCAAAAGGCGTCGTCACCGCCGAAATGGCTTCAACCGGCGTAAACAATGAAAT 660

allele 31 TACGGTCGCAAAAGGCGTCGTTACCGCCCAAATGAATCCAAGCGGCGTAAACAATGAAAT 658

allele 30 TACGGTCGCAAACGGCGTCGTTACCGCCGAAATGAAATCAGACGGCGTAAACAAAGAAAT 660

allele 32 TACGGTCGCAAACGGCGTCGTTACCGCCGAAATGAAATCAGACGGCGTAAACAAAGAAAT 660

************ ******** ****** ***** ** ************ *****

allele 1 CAAAGGCAAAAAACTCTCCCTGTGGGCCAAGCGTGAAGACGGTTCGGTAAAATGGTTCTG 720

allele 3 CAAAGGCAAAAAACTCTCCCTGTGGGCCAAGCGTGAAGACGGTTCGGTAAAATGGTTCTG 720

allele 4 CAAAGGCAAAAAACTCTCCCTGTGGGCCAAGCGTCAAGACGGTTCGGTAAAATGGTTCTG 720

allele 31 CAAAGACAAAAAACTCTCCCTGTGGGCCAAGCGTGAAAACGGTTCGGTAAAATGGTTCTG 718

allele 30 CAAAGGCAAAAAACTCTCCCTGTGGGGCAGGCGTGAAAACGGTTCGGTAAAATGGTTCTG 720

allele 32 CCAAGGCAAAAGACTCTCCCTGTGGGCCAGGCGTGAAGCCGGTTCGGTAAAATGGTTCTG 720

* *** ***** ************** ** **** ** *********************

allele 1 CGGACAGCCGGTTAAGCGCGAC---GCCG---GCGCCAAA------------GCCGACGA 762

allele 3 CGGACAGCCGGTTAAGCGCGAC---GCCG---GCGCCAAAA------CCGGCGCCGACGA 768

allele 4 CGGACAGCCGGTTAAGCGCGAC---GCCG---GCGCCAAA------------GCCGACGA 762

allele 31 CGGACAGCCGGTTACGCGCAAC---G------ACGCCAAA------------GCCGACGA 757

allele 30 CGGACAGCCGGTTAAGCGCGAC---GCCAACAACGCCAACAACGACGCCGTCACCGACGA 777

allele 32 CGGACAGCCGGTTACGCGCGCCAAAGCCAAAGACGCCGACGA------CGTTACCGACGA 774

************** **** * * **** * *******

allele 1 CGTCAAAGCCGAC---GCCGCCAACGCCA---TCGAAACCAAGCACCTGCCGTCAACCTG 816

allele 3 CGTCAAAGCCGAC---GGCAACAACGGCA---TCAACACCAAGCACCTGCCGTCAACCTG 822

allele 4 CGTCAAAGCCGAC---GCCGCCAACGCCA---TCGAAACCAAGCACCTGCCGTCAACCTG 816

allele 31 CGTCAAAGCCGAC---GCCGCCAACGCCA---TCGAAACCAAGCACCTGCCGTCAACCTG 811

allele 30 CACCACCGGCAAC---GGCAACGAAAAAA---TCGAAACCAAGCACCTGCCGTCAACCTG 831

allele 32 CGCCGGCACCGACAACGGCGGCAAAGGCAAAATCGACACCAAGCACCTGCCGTCAACCTG 834

* * * ** * * * * * ** * ***********************

allele 1 CCGCGATGAATCATC-TGCCACCTAAGGCAAATTAGGCCTTAAATTTTAAATAAATCAAG 875

allele 3 CCGCGATAAA-CATGATGCCAAATGAGGCAAATTAGGCCTTAAATTTTAAATAAATCAAG 881

allele 4 CCGCGATGAATCATC-TGCCACCTAAGGCAAATTAGGCCTTAAATTTTAAATAAATCAAG 875

allele 31 CCGCGATGAATCATC-TGCCACCTAAGGCAAATTAGGCCTTAAATTTTAAATAAATCAAG 870

allele 30 CCGCGATGAATCATC-TGCCACCTAAGGCAAATTAGGCCTTAAATTTTAAATAAATCAAG 890

allele 32 CCGCGATAAATCA-ACTGCCAAATAAGGCAAATTAGGCCTTAAATTTTAAATAAATCAAA 893

******* ** ** ***** * **********************************

allele 1 CGGTAAGTGATTTCCCACGGCCGCCCGGATCAACCCGGGCGGCTTGTCTTTTAAGGGTTT 935

allele 3 CGGTAAGTGATTTCCCACGGCCGCCCGGATCAACCCGGGCGGCTTGTCTTTTAAGGGTTT 941

allele 4 CGGTAAGTGATTTCCCACGGCCGCCCGGATCAACCCGGGCGGCTTGTCTTTTAAGGGTTT 935

allele 31 CGGTAAGTGATTTCCCACGGCCGCCCGGATCAACCCGGGCGGCTTGTCTTTTAAGGGTTT 930

allele 30 CGGTAAGTGATTTCCCACGGCCGCCCGGATCAACCCGGGCGGCTTGTCTTTTAAGGGTTT 950

allele 32 CGGTAAGTGATTTCCCACGGCCGCCCGGATCAACCCGGGCGGCTTGTCTTTTAAGGGTTT 953

************************************************************

allele 1 GCAAGGCGGGCGGGGTCGTCCGTTCCGGTGGAAATAATATATCGATTGCGCTTCAAGGCC 995

allele 3 GCAAGGCGGGCGGGGTCGTCCGTTCCGGTGGAAATAATATATCGATTGCGCTTCAAGGCC 1001

allele 4 GCAAGGCGGGCGGGGTCGTCCGTTCCGGTGGAAATAATATATCGATTGCGCTTCAAGGCC 995

allele 31 GCAAGGCGGGCGGGGTCGTCCGTTCCGGTGGAAATAATATATCGATTGCGCTTCAAGGCC 990

allele 30 GCAAGGCGGGCGGGGTCGTCCGTTCCGGTGGAAATAATATATCGATTGCGCTTCAAGGCC 1010

allele 32 GCAAGGCGGGCGGGGTCGTCCGTTCCGGTGGAAATAATATATCGATTGCGCTTCAAGGCC 1013

************************************************************

allele 1 CTGCATGTGCCTCATTGCCACCCGTTTAAACACGGTTTTTATCTGACAGGCGCGCAATCC 1055

allele 3 CTGCATGTGCCTCATTGCCACCCGTTTAAACACGGTTTTTATCTGACAGGCGCGCAATCC 1061

allele 4 CTGCATGTGCCTCATTGCCACCCGTTTAAACACGGTTTTTATCTGACAGGCGCGCAATCC 1055

allele 31 CTGCATGTGCCTCATTGCCACCCGTTTAAACACGGTTTTTATCTGACAGGCGCGCAATCC 1050

allele 30 CTGCATGTGCCTCATTGCCACCCGTTTAAACACGGTTTTTATCTGACAGGCGCGCAATCC 1070

allele 32 CTGCATGTGCCTCATTGCCACCCGTTTAAACACGGTTTTTATCTGACAGGCGCGCAATCC 1073

************************************************************

allele 1 GCCCCCTCATTTGTTAATCCGCCATATTGTATTGAAACACCGCCCGGAACCCGATATAAT 1115

allele 3 GCCCCCTCATTTGTTAATCCGCCATATTGTATTGAAACACCGCCCGGAACCCGATATAAT 1121

allele 4 GCCCCCTCATTTGTTAATCCGCCATATTGTATTGAAACACCGCCCGGAACCCGATATAAT 1115

allele 31 GCCCCCTCATTTGTTAATCCGCCATATTGTATTGAAACACCGCCCGGAACCCGATATAAT 1110

allele 30 GCCCCCTCATTTGTTAATCCGCCATATTGTATTGAAACACCGCCCGGAACCCGATATAAT 1130

allele 32 GCCCCCTCATTTGTTAATCCGCCATATTGTATTGAAACACCGCCCGGAACCCGATATAAT 1133

************************************************************

allele 1 CCGCCCTTCAACATCAGTGAAAATCTTTTTTTAACCGGTTAAACCGAATAAGGAGCCGAA 1175

allele 3 CCGCCCTTCAACATCAGTGAAAATCTTTTTTTAACCGGTTAAACCGAATAAGGAGCCGAA 1181

allele 4 CCGCCCTTCAACATCAGTGAAAATCTTTTTTTAACCGGTTAAACCGAATAAGGAGCCGAA 1175

allele 31 CCGCCCTTCAACATCAGTGAAAATCTTTTTTTAACCGGTTAAACCGAATAAGGAGCCGAA 1170

allele 30 CCGCCCTTCAACATCAGTGAAAATCTTTTTTTAACCGGTTAAACCGAATAAGGAGCCGAA 1190

allele 32 CCGCCCTTCAACATCAGTGAAAATCTTTTTTTAACCGGTTAAACCGAATAAGGAGCCGAA 1193

************************************************************

**Figure S4:** Alignment of the variant sequences detected in the repeat experiment with *pilE* in *N. gonorrhoeae* FA1090. The seq1 assembly is identical to the reference sequence obtained by Sanger sequencing of the amplicon. Blue text indicates sequence flanking the *pilE* gene (black text). Sequence differences are highlighted in yellow. The grey shading highlights the extent of the sequence identity between the *pilE* sequence and the various silent copies, flanking the variant sequence.
