# Supplementary material for: The Use of High-Throughput DNA Sequencing in the Investigation of Antigenic Variation: Application to Neisseria Species
Source: PLoS One. 2014 Jan 22;9(1):e86704. doi: 10.1371/journal.pone.0086704 (PMC3899283; doi:10.1371/journal.pone.0086704)
Supplement: Figure S5 — Alignment of the variant sequences detected in the first experiment with pilE in N. gonorrhoeae MS11. The allele 1 assembly is identical to the reference sequence obtained by Sanger sequencing of the amplicon. Blue text indicates sequence flanking the pilE gene (black text). Sequence differences are highlighted in yellow. The grey shading highlights the extent of the sequence identity between the pilE sequence and the various silent copies, flanking the variant sequence. (DOC) [file pone.0086704.s005.doc]

Allele 5 CTTTCAATTAGGAGTAATTTTATGAATACCCTTCAAAAAGGCTTTACCCTTATCGAGCTG 60

Allele 7 CTTTCAATTAGGAGTAATTTTATGAATACCCTTCAAAAAGGCTTTACCCTTATCGAGCTG 60

Allele 1 CTTTCAATTAGGAGTAATTTTATGAATACCCTTCAAAAAGGCTTTACCCTTATCGAGCTG 60

Allele 10 CTTTCAATTAGGAGTAATTTTATGAATACCCTTCAAAAAGGCTTTACCCTTATCGAGCTG 60

Allele 14 CTTTCAATTAGGAGTAATTTTATGAATACCCTTCAAAAAGGCTTTACCCTTATCGAGCTG 60

Allele 11 CTTTCAATTAGGAGTAATTTTATGAATACCCTTCAAAAAGGCTTTACCCTTATCGAGCTG 60

Allele 13 CTTTCAATTAGGAGTAATTTTATGAATACCCTTCAAAAAGGCTTTACCCTTATCGAGCTG 60

Allele 15 CTTTCAATTAGGAGTAATTTTATGAATACCCTTCAAAAAGGCTTTACCCTTATCGAGCTG 60

Allele 8 CTTTCAATTAGGAGTAATTTTATGAATACCCTTCAAAAAGGCTTTACCCTTATCGAGCTG 60

Allele 12 CTTTCAATTAGGAGTAATTTTATGAATACCCTTCAAAAAGGCTTTACCCTTATCGAGCTG 60

Allele 4 CTTTCAATTAGGAGTAATTTTATGAATACCCTTCAAAAAGGCTTTACCCTTATCGAGCTG 60

Allele 9 CTTTCAATTAGGAGTAATTTTATGAATACCCTTCAAAAAGGCTTTACCCTTATCGAGCTG 60

Allele 3 CTTTCAATTAGGAGTAATTTTATGAATACCCTTCAAAAAGGCTTTACCCTTATCGAGCTG 60

Allele 2 CTTTCAATTAGGAGTAATTTTATGAATACCCTTCAAAAAGGCTTTACCCTTATCGAGCTG 60

Allele 6 CTTTCAATTAGGAGTAATTTTATGAATACCCTTCAAAAAGGCTTTACCCTTATCGAGCTG 60

************************************************************

Allele 5 ATGATTGTGATCGCTATCGTCGGCATTTTGGCGGCAGTCGCCCTTCCCGCCTACCAAGAC 120

Allele 7 ATGATTGTGATCGCTATCGTCGGCATTTTGGCGGCAGTCGCCCTTCCCGCCTACCAAGAC 120

Allele 1 ATGATTGTGATCGCTATCGTCGGCATTTTGGCGGCAGTCGCCCTTCCCGCCTACCAAGAC 120

Allele 10 ATGATTGTGATCGCTATCGTCGGCATTTTGGCGGCAGTCGCCCTTCCCGCCTACCAAGAC 120

Allele 14 ATGATTGTGATCGCTATCGTCGGCATTTTGGCGGCAGTCGCCCTTCCCGCCTACCAAGAC 120

Allele 11 ATGATTGTGATCGCTATCGTCGGCATTTTGGCGGCAGTCGCCCTTCCCGCCTACCAAGAC 120

Allele 13 ATGATTGTGATCGCTATCGTCGGCATTTTGGCGGCAGTCGCCCTTCCCGCCTACCAAGAC 120

Allele 15 ATGATTGTGATCGCTATCGTCGGCATTTTGGCGGCAGTCGCCCTTCCCGCCTACCAAGAC 120

Allele 8 ATGATTGTGATCGCTATCGTCGGCATTTTGGCGGCAGTCGCCCTTCCCGCCTACCAAGAC 120

Allele 12 ATGATTGTGATCGCTATCGTCGGCATTTTGGCGGCAGTCGCCCTTCCCGCCTACCAAGAC 120

Allele 4 ATGATTGTGATCGCTATCGTCGGCATTTTGGCGGCAGTCGCCCTTCCCGCCTACCAAGAC 120

Allele 9 ATGATTGTGATCGCTATCGTCGGCATTTTGGCGGCAGTCGCCCTTCCCGCCTACCAAGAC 120

Allele 3 ATGATTGTGATCGCTATCGTCGGCATTTTGGCGGCAGTCGCCCTTCCCGCCTACCAAGAC 120

Allele 2 ATGATTGTGATCGCTATCGTCGGCATTTTGGCGGCAGTCGCCCTTCCCGCCTACCAAGAC 120

Allele 6 ATGATTGTGATCGCTATCGTCGGCATTTTGGCGGCAGTCGCCCTTCCCGCCTACCAAGAC 120

************************************************************

Allele 5 TACACCGCCCGCGCGCAAGTTTCCGAAGCCATCCTTTTGGCCGAAGGTCAAAAATCAGCC 180

Allele 7 TACACCGCCCGCGCGCAAGTTTCCGAAGCCATCCTTTTGGCCGAAGGTCAAAAATCAGCC 180

Allele 1 TACACCGCCCGCGCGCAAGTTTCCGAAGCCATCCTTTTGGCCGAAGGTCAAAAATCAGCC 180

Allele 10 TACACCGCCCGCGCGCAAGTTTCCGAAGCCATCCTTTTGGCCGAAGGTCAAAAATCAGCC 180

Allele 14 TACACCGCCCGCGCGCAAGTTTCCGAAGCCATCCTTTTGGCCGAAGGTCAAAAATCAGCC 180

Allele 11 TACACCGCCCGCGCGCAAGTTTCCGAAGCCATCCTTTTGGCCGAAGGTCAAAAATCAGCC 180

Allele 13 TACACCGCCCGCGCGCAAGTTTCCGAAGCCATCCTTTTGGCCGAAGGTCAAAAATCAGCC 180

Allele 15 TACACCGCCCGCGCGCAAGTTTCCGAAGCCATCCTTTTGGCCGAAGGTCAAAAATCAGCC 180

Allele 8 TACACCGCCCGCGCGCAAGTTTCCGAAGCCATCCTTTTGGCCGAAGGTCAAAAATCAGCC 180

Allele 12 TACACCGCCCGCGCGCAAGTTTCCGAAGCCATCCTTTTGGCCGAAGGTCAAAAATCAGCC 180

Allele 4 TACACCGCCCGCGCGCAAGTTTCCGAAGCCATCCTTTTGGCCGAAGGTCAAAAATCAGCC 180

Allele 9 TACACCGCCCGCGCGCAAGTTTCCGAAGCCATCCTTTTGGCCGAAGGTCAAAAATCAGCC 180

Allele 3 TACACCGCCCGCGCGCAAGTTTCCGAAGCCATCCTTTTGGCCGAAGGTCAAAAATCAGCC 180

Allele 2 TACACCGCCCGCGCGCAAGTTTCCGAAGCCATCCTTTTGGCCGAAGGTCAAAAATCAGCC 180

Allele 6 TACACCGCCCGCGCGCAAGTTTCCGAAGCCATCCTTTTGGCCGAAGGTCAAAAATCAGCC 180

*************************************** ** ** ************

Allele 5 GTCACCGAGTATTACCTGAATCACGGCAAATGGCCGGAAAACAACACTTCTGCCGGCGTG 240

Allele 7 GTCACCGAGTATTACCTGAATCACGGCAAATGGCCGGAAAACAACACTTCTGCCGGCGTG 240

Allele 1 GTCACCGAGTATTACCTGAATCACGGCAAATGGCCGGAAAACAACACTTCTGCCGGCGTG 240

Allele 10 GTCACCGAGTATTACCTGAATCACGGCAAATGGCCGGAAAACAACACTTCTGCCGGCGTG 240

Allele 14 GTCACCGAGTATTACCTGAATCACGGCAAATGGCCGGAAAACAACACTTCTGCCGGCGTG 240

Allele 11 GTCACCGAGTATTACCTGAATCACGGCAAATGGCCGGAAAACAACACTTCTGCCGGCGTG 240

Allele 13 GTCACCGAGTATTACCTGAATCACGGCAAATGGCCGGAAAACAACACTTCTGCCGGCGTG 240

Allele 15 GTCACCGAGTATTACCTGAATCACGGCAAATGGCCGGAAAACAACACTTCTGCCGGCGTG 240

Allele 8 GTCACCGAGTATTACCTGAATCACGGCAAATGGCCGGAAAACAACACTTCTGCCGGCGTG 240

Allele 12 GTCACCGAGTATTACCTGAATCACGGCAAATGGCCGGAAAACAACACTTCTGCCGGCGTG 240

Allele 4 GTCACCGAGTATTACCTGAATCACGGCGAATGGCCCAAAGACAACGACTCTGCCGGCGTG 240

Allele 9 GTCACCGAGTATTACCTGAATCACGGCATATGGCCGAAAGACAACACTTCTGCCGGCGTG 240

Allele 3 GTCACCGAGTATTACCTGAATCACGGCGAATGGCCGGAAGACAACACTTCTGCCGGCGTG 240

Allele 2 GTTACCGAGTATTACCTGAATCACGGCGAATGGCCGAAAGACAACACTTCTGCCGGCGTG 240

Allele 6 GTCACCGAGTATTACCTGAATCACGGCAAATGGCCGGAAAACAACACTTCTGCCGGCGTG 240

** ************************ ****** ** ***** ************

Allele 5 GCATCCCCCCCCTCCGACATCAAAGGCAAATATGTTAAAGAGGTTGAAGTTAAAAACGGC 300

Allele 7 GCATCCCCCCCCTCCGACATCAAAGGCAAATATGTTAAAGAGGTTGAAGTTAAAAACGGC 300

Allele 1 GCATCCCCCCCCTCCGACATCAAAGGCAAATATGTTAAAGAGGTTGAAGTTAAAAACGGC 300

Allele 10 GCATCCCCCCCCTCCGACATCAAAGGCAAATATGTTAAAGAGGTTGAAGTTAAAAACGGC 300

Allele 14 GCATCCCCCCCCTCCGACATCAAAGGCAAATATGTTAAAGAGGTTGAAGTTAAAAACGGC 300

Allele 11 GCATCCCCCCCCTCCGACATCAAAGGCAAATATGTTAAAGAGGTTGAAGTTAAAAACGGC 300

Allele 13 GCATCCCCCCCCTCCGACATCAAAGGCAAATATGTTAAAGAGGTTGAAGTTAAAAACGGC 300

Allele 15 GCATCCCCCCCCTCCGACATCAAAGGCAAATATGTTAAAGAGGTTGAAGTTAAAAACGGC 300

Allele 8 GCATCCCCCCCCTCCGACATCAAAGGCAAATATGTTAAAGAGGTTGAAGTTAAAAACGGC 300

Allele 12 GCATCCCCCCCCTCCGACATCAAAGGCAAATATGTTAAAGAGGTTGAAGTTAAAAACGGC 300

Allele 4 GCATCCGCTTCAA---AAATCATAGGCAAATATGTTAAGCAAGTTGAAGTCAAAAACGGC 297

Allele 9 GCATCTTCTTCAT---CAATCAAAGGCAAATATGTTAAGGAAGTTAAAGTCGAAAACGGC 297

Allele 3 GCATCCTCCCCCACCGACATCAAAGGCAAATATGTTCAAAGCGTTACGGTCGCAAACGGC 300

Allele 2 GCATCCTCCGACA---AAATCAAAGGCAAATATGTTCAGAAAGTTGAAGTCGCAAAAGGC 297

Allele 6 GCATCCCCCGCCG---AAATCAAAGGCAAATATGTTAAAAGCGTTACGGTCGCAAAAGGC 297

***** * **** ************* * *** ** *** ***

Allele 5 GTCGTTACCGCCACAATGCTTTCAAGCGGCGTAAACAATGAAATCAAAGGCAAAAAACTC 360

Allele 7 GTCGTTACCGCCACAATGCTTTCAAGCGGCGTAAACAATGAAATCAAAGGCAAAAAACTC 360

Allele 1 GTCGTTACCGCCACAATGCTTTCAAGCGGCGTAAACAATGAAATCAAAGGCAAAAAACTC 360

Allele 10 GTCGTTACCGCCACAATGCTTTCAAGCGGCGTAAACAATGAAATCAAAGGCAAAAAACTC 360

Allele 14 GTCGTTACCGCCACAATGCTTTCAAGCGGCGTAAACAATGAAATCAAAGGCAAAAAACTC 360

Allele 11 GTCGTTACCGCCACAATGCTTTCAAGCGGCGTAAACAATGAAATCAAAGGCAAAAAACTC 360

Allele 13 GTCGTTACCGCCACAATGCTTTCAAGCGGCGTAAACAATGAAATCAAAGGCAAAAAACTC 360

Allele 15 GTCGTTACCGCCACAATGCTTTCAAGCGGCGTAAACAATGAAATCAAAGGCAAAAAACTC 360

Allele 8 GTCGTTACCGCCACAATGCTTTCAAGCGGCGTAAACAATGAAATCAAAGGCAAAAAACTC 360

Allele 12 GTCGTTACCGCCACAATGCTTTCAAGCGGCGTAAACAATGAAATCAAAGGCAAAAAACTC 360

Allele 4 GTCGTTACCGCCCAAATGAAATCAGACGGCGTAAACAAAGAAATCAAAAACAAAAAACTC 357

Allele 9 GTCGTCACCGCCACAATGAATTCAAGCGGCGTAAACAAAGAAATCCAAGGCAAAAGACTC 357

Allele 3 GTCGTTACCGCCGAAATGGCTTCAACCGGCGTAAACAAAGAAATCCAAGGCAAAAGACTC 360

Allele 2 GTCGTTACCGCCACAATGGCTTCAAGCAACGTAAACAAAGAAATCAAAGACAAAAAACTC 357

Allele 6 GTCGTTACCGCCCAAATGAATCCAAGCGGCGTAAACAATGAAATCAAAGACAAAAAACTC 357

***** ****** **** ** * ********* ****** ** ***** ****

Allele 5 TCCCTGTGGGCCAGGCGTGAAAACGGTTCGGTAAAATGGTTCTGCGGACAGCCGGTTACG 420

Allele 7 TCCCTGTGGGCCAGGCGTGAAAACGGTTCGGTAAAATGGTTCTGCGGACAGCCGGTTACG 420

Allele 1 TCCCTGTGGGCCAGGCGTGAAAACGGTTCGGTAAAATGGTTCTGCGGACAGCCGGTTACG 420

Allele 10 TCCCTGTGGGCCAGGCGTGAAAACGGTTCGGTAAAATGGTTCTGCGGACAGCCGGTTACG 420

Allele 14 TCCCTGTGGGCCAGGCGTGAAAACGGTTCGGTAAAATGGTTCTGCGGACAGCCGGTTACG 420

Allele 11 TCCCTGTGGGCCAGGCGTGAAAACGGTTCGGTAAAATGGTTCTGCGGACAGCCGGTTACG 420

Allele 13 TCCCTGTGGGCCAGGCGTGAAAACGGTTCGGTAAAATGGTTCTGCGGACAGCCGGTTACG 420

Allele 15 TCCCTGTGGGCCAGGCGTGAAAACGGTTCGGTAAAATGGTTCTGCGGACAGCCGGTTAAG 420

Allele 8 TCCCTGTGGGCCAGGCGTGAAAACGGTTCGGTAAAATGGTTCTGCGGACAGCCGGTTACG 420

Allele 12 TCCCTGTGGGCCAGGCGTGAAAACGGTTCGGTAAAATGGTTCTGCGGACAGCCGGTTACG 420

Allele 4 TCCCTGTGGGCCAAGCGTGAAAACGGTTCGGTAAAATGGTTCTGCGGACAGCCGGTTACG 417

Allele 9 TCCCTGTGGGCCAAGCGTGAAAACGGTTCGGTAAAATGGTTCTGCGGACAGCCGGTTACG 417

Allele 3 TCCCTGTGGGCCAAGCGTGAAAACGGTTCGGTAAAATGGTTCTGCGGACAGCCGGTTACG 420

Allele 2 TCCCTGTGGGCCAGGCGTCAAGACGGTTCGGTAAAATGGTTCTGCGGACAGCCGGTTACG 417

Allele 6 TCCCTGTGGGCCAGGCGTGAAAACGGTTCGGTAAAATGGTTCTGCGGACAGCCGGTTACG 417

************* **** ** ************************************ *

Allele 5 CGCACC---GACGACGACA---------------CCGTTGCCGACGCCAAAGACGGCA-- 460

Allele 7 CGCACC---GACGACGACA---------------CCGTTGCCGACGCCAAAGACGGCA-- 460

Allele 1 CGCACC---GACGACGACA---------------CCGTTGCCGACGCCAAAGACGGCA-- 460

Allele 10 CGCGCC---GCCAAAGACG---------------ACGACGCCGTCACCGCCGACGGCA-- 460

Allele 14 CGCGCCAAAGCCAAAGACGCCGACGACGTTACCGACGACGCCGGCACCGACAACGGCG-- 478

Allele 11 CGCAAC---GCCAACGACG------------------ACACCGTCACCGCCGACGGCACC 459

Allele 13 CGCAAC---GCCAAAGCC---------------AACGACACCGTTGCCGCCGACGGCACC 462

Allele 15 CGCAAC---GACAACGCCG------------ACAACGACGACGTTACCGCCGACGGCACC 465

Allele 8 CGCGGCGCCGGCAACGCCGGC---------AAAGCCGACGACGTCACCAAAGCCGGCAAC 471

Allele 12 CGCACC---GACGACGCCG---------------CCAAAGACGCCGTTACCGCCGACGC- 461

Allele 4 CGCACC---GACGACGACA---------------CCGTTGCCGACGCCAAAGACGGCA-- 457

Allele 9 CGCACC---GACGACGACA---------------CCGTTGCCGACGCCAAAGACGGCA-- 457

Allele 3 CGCACC---GACGACGACA---------------CCGTTGCCGACGCCAAAGACGGCA-- 460

Allele 2 CGCACC---GACGACGACA---------------CCGTTGCCGACGCCAAAGACGGCA-- 457

Allele 6 CGCACC---GACGACGACA---------------CCGTTGCCGACGCCAAAGACGGCA-- 457

*** * * * * * * ** ** *

Allele 5 ----------AAGAAATCGACACCAAGCACCTGCCGTCAACCTGCCGCGATAA---CTTT 507

Allele 7 ----------AAGAAATCGACACCAAGCACCTGCCGTCAACCTGCCGCGACA----CTTC 506

Allele 1 ----------AAGAAATCGACACCAAGCACCTGCCGTCAACCTGCCGCGATAAGGCATCT 510

Allele 10 ----ACAA---CAAAATCGACACCAAGCACCTGCCGTCAACCTGCCGCGATAAGGCATCT 513

Allele 14 ----GCAAAGGCAAAATCGACACCAAGCACCTGCCGTCAACCTGCCGCGATAAGGCATCT 534

Allele 11 GGCAACGACGGCAAAATCGACACCAAGCACCTGCCGTCAACCTGCCGCGATAAGGCATCT 519

Allele 13 G---GCAACGACAAAATCGAAACCAAGCACCTGCCGTCAACCTGCCGCGATAAGGCATCT 519

Allele 15 GACGGCAAAGACAAAATCGAAACCAAGCACCTGCCGTCAACCTGCCGCGATAAGGCATCT 525

Allele 8 G---ACAACGAAAAAATCAACACCAAGCACCTGCCGTCAACCTGCCGCGATAAGGCATCT 528

Allele 12 -----CAAAGACGCCATCGAAACCAAGCACCTGCCGTCAACCTGCCGCGATAAGGCATCT 516

Allele 4 ----------AAGAAATCGACACCAAGCACCTGCCGTCAACCTGCCGCGATAAGGCATCT 507

Allele 9 ----------AAGAAATCGACACCAAGCACCTGCCGTCAACCTGCCGCGATAAGGCATCT 507

Allele 3 ----------AAGAAATCGACACCAAGCACCTGCCGTCAACCTGCCGCGATAAGGCATCT 510

Allele 2 ----------AAGAAATCGACACCAAGCACCTGCCGTCAACCTGCCGCGATAAGGCATCT 507

Allele 6 ----------AAGAAATCGACACCAAGCACCTGCCGTCAACCTGCCGCGATAAGGCATCT 507

*** * ***************************** * *

Allele 5 GATGCCAGCTGAGGCAAATTAGGCCTTAAATTTCAAATAAATCAAACGGTAAGTGATTTT 567

Allele 7 ----------------------------------------ATCTGCCGGTAAGTGATTTT 526

Allele 1 GATGCCAAATGAGGCAAATTAGGCCTTAAATTTTAAATAAATCAAGCGGTAAGTGATTTT 570

Allele 10 GATGCCAAATGAGGCAAATTAGGCCTTAAATTTTAAATAAATCAAGCGGTAAGTGATTTT 573

Allele 14 GATGCCAAATGAGGCAAATTAGGCCTTAAATTTTAAATAAATCAAGCGGTAAGTGATTTT 594

Allele 11 GATGCCAAATGAGGCAAATTAGGCCTTAAATTTTAAATAAATCAAGCGGTAAGTGATTTT 579

Allele 13 GATGCCAAATGAGGCAAATTAGGCCTTAAATTTTAAATAAATCAAGCGGTAAGTGATTTT 579

Allele 15 GATGCCAAATGAGGCAAATTAGGCCTTAAATTTTAAATAAATCAAGCGGTAAGTGATTTT 585

Allele 8 GATGCCAAATGAGGCAAATTAGGCCTTAAATTTTAAATAAATCAAGCGGTAAGTGATTTT 588

Allele 12 GATGCCAAATGAGGCAAATTAGGCCTTAAATTTTAAATAAATCAAGCGGTAAGTGATTTT 576

Allele 4 GATGCCAAATGAGGCAAATTAGGCCTTAAATTTTAAATAAATCAAGCGGTAAGTGATTTT 567

Allele 9 GATGCCAAATGAGGCAAATTAGGCCTTAAATTTTAAATAAATCAAGCGGTAAGTGATTTT 567

Allele 3 GATGCCAAATGAGGCAAATTAGGCCTTAAATTTTAAATAAATCAAGCGGTAAGTGATTTT 570

Allele 2 GATGCCAAATGAGGCAAATTAGGCCTTAAATTTTAAATAAATCAAGCGGTAAGTGATTTT 567

Allele 6 GATGCCAAATGAGGCAAATTAGGCCTTAAATTTTAAATAAATCAAGCGGTAAGTGATTTT 567

*** **************

Allele 5 CCACGGCCGCCCGGATCAACCCGGGCGGCTTGTCTTTTAAGGGTTTGCAAGGCGGGCGGG 627

Allele 7 CCACGGCCGCCCGGATCAACCCGGGCGGCTTGTCTTTTAAGGGTTTGCAAGGCGGGCGGG 586

Allele 1 CCACGGCCGCCCGGATCAACCCGGGCGGCTTGTCTTTTAAGGGTTTGCAAGGCGGGCGGG 630

Allele 10 CCACGGCCGCCCGGATCAACCCGGGCGGCTTGTCTTTTAAGGGTTTGCAAGGCGGGCGGG 633

Allele 14 CCACGGCCGCCCGGATCAACCCGGGCGGCTTGTCTTTTAAGGGTTTGCAAGGCGGGCGGG 654

Allele 11 CCACGGCCGCCCGGATCAACCCGGGCGGCTTGTCTTTTAAGGGTTTGCAAGGCGGGCGGG 639

Allele 13 CCACGGCCGCCCGGATCAACCCGGGCGGCTTGTCTTTTAAGGGTTTGCAAGGCGGGCGGG 639

Allele 15 CCACGGCCGCCCGGATCAACCCGGGCGGCTTGTCTTTTAAGGGTTTGCAAGGCGGGCGGG 645

Allele 8 CCACGGCCGCCCGGATCAACCCGGGCGGCTTGTCTTTTAAGGGTTTGCAAGGCGGGCGGG 648

Allele 12 CCACGGCCGCCCGGATCAACCCGGGCGGCTTGTCTTTTAAGGGTTTGCAAGGCGGGCGGG 636

Allele 4 CCACGGCCGCCCGGATCAACCCGGGCGGCTTGTCTTTTAAGGGTTTGCAAGGCGGGCGGG 627

Allele 9 CCACGGCCGCCCGGATCAACCCGGGCGGCTTGTCTTTTAAGGGTTTGCAAGGCGGGCGGG 627

Allele 3 CCACGGCCGCCCGGATCAACCCGGGCGGCTTGTCTTTTAAGGGTTTGCAAGGCGGGCGGG 630

Allele 2 CCACGGCCGCCCGGATCAACCCGGGCGGCTTGTCTTTTAAGGGTTTGCAAGGCGGGCGGG 627

Allele 6 CCACGGCCGCCCGGATCAACCCGGGCGGCTTGTCTTTTAAGGGTTTGCAAGGCGGGCGGG 627

************************************************************

Allele 5 GTCGTCCGTTCCGGTGGAAATAATATAT 655

Allele 7 GTCGTCCGTTCCGGTGGAAATAATATAT 614

Allele 1 GTCGTCCGTTCCGGTGGAAATAATATAT 658

Allele 10 GTCGTCCGTTCCGGTGGAAATAATATAT 661

Allele 14 GTCGTCCGTTCCGGTGGAAATAATATAT 682

Allele 11 GTCGTCCGTTCCGGTGGAAATAATATAT 667

Allele 13 GTCGTCCGTTCCGGTGGAAATAATATAT 667

Allele 15 GTCGTCCGTTCCGGTGGAAATAATATAT 673

Allele 8 GTCGTCCGTTCCGGTGGAAATAATATAT 676

Allele 12 GTCGTCCGTTCCGGTGGAAATAATATAT 664

Allele 4 GTCGTCCGTTCCGGTGGAAATAATATAT 655

Allele 9 GTCGTCCGTTCCGGTGGAAATAATATAT 655

Allele 3 GTCGTCCGTTCCGGTGGAAATAATATAT 658

Allele 2 GTCGTCCGTTCCGGTGGAAATAATATAT 655

Allele 6 GTCGTCCGTTCCGGTGGAAATAATATAT 655

****************************

**Figure S5:** Alignment of the variant sequences detected in the first experiment with *pilE* in *N. gonorrhoeae* MS11. The allele 1 assembly is identical to the reference sequence obtained by Sanger sequencing of the amplicon. Blue text indicates sequence flanking the *pilE* gene (black text). Sequence differences are highlighted in yellow. The grey shading highlights the extent of the sequence identity between the *pilE* sequence and the various silent copies, flanking the variant sequence.
