# Supplementary material for: The Use of High-Throughput DNA Sequencing in the Investigation of Antigenic Variation: Application to Neisseria Species
Source: PLoS One. 2014 Jan 22;9(1):e86704. doi: 10.1371/journal.pone.0086704 (PMC3899283; doi:10.1371/journal.pone.0086704)
Supplement: Figure S6 — Alignment of the variant sequences detected in the repeat experiment with pilE in N. gonorrhoeae MS11. The allele 1 assembly is identical to the reference sequence obtained by Sanger sequencing of the amplicon. Blue text indicates sequence flanking the pilE gene (black text). Sequence differences are highlighted in yellow. The grey shading highlights the extent of the sequence identity between the pilE sequence and the various silent copies, flanking the variant sequence. (DOC) [file pone.0086704.s006.doc]

Allele 1 AATTAGGAGTCCCCTTTCAATTAGGAGTCCCCTTTCAATTAGGAGTAATTTTATGAATAC 60

Allele 11 AATTAGGAGTCCCCTTTCAATTAGGAGTCCCCTTTCAATTAGGAGTAATTTTATGAATAC 60

Allele 19 AATTAGGAGTCCCCTTTCAATTAGGAGTCCCCTTTCAATTAGGAGTAATTTTATGAATAC 60

Allele 17 AATTAGGAGTCCCCTTTCAATTAGGAGTCCCCTTTCAATTAGGAGTAATTTTATGAATAC 60

Allele 20 AATTAGGAGTCCCCTTTCAATTAGGAGTCCCCTTTCAATTAGGAGTAATTTTATGAATAC 60

Allele 16 AATTAGGAGTCCCCTTTCAATTAGGAGTCCCCTTTCAATTAGGAGTAATTTTATGAATAC 60

Allele 9 AATTAGGAGTCCCCTTTCAATTAGGAGTCCCCTTTCAATTAGGAGTAATTTTATGAATAC 60

Allele 18 AATTAGGAGTCCCCTTTCAATTAGGAGTCCCCTTTCAATTAGGAGTAATTTTATGAATAC 60

************************************************************

Allele 1 CCTTCAAAAAGGCTTTACCCTTATCGAGCTGATGATTGTGATCGCTATCGTCGGCATTTT 120

Allele 11 CCTTCAAAAAGGCTTTACCCTTATCGAGCTGATGATTGTGATCGCTATCGTCGGCATTTT 120

Allele 19 CCTTCAAAAAGGCTTTACCCTTATCGAGCTGATGATTGTGATCGCTATCGTCGGCATTTT 120

Allele 17 CCTTCAAAAAGGCTTTACCCTTATCGAGCTGATGATTGTGATCGCTATCGTCGGCATTTT 120

Allele 20 CCTTCAAAAAGGCTTTACCCTTATCGAGCTGATGATTGTGATCGCTATCGTCGGCATTTT 120

Allele 16 CCTTCAAAAAGGCTTTACCCTTATCGAGCTGATGATTGTGATCGCTATCGTCGGCATTTT 120

Allele 9 CCTTCAAAAAGGCTTTACCCTTATCGAGCTGATGATTGTGATCGCTATCGTCGGCATTTT 120

Allele 18 CCTTCAAAAAGGCTTTACCCTTATCGAGCTGATGATTGTGATCGCTATCGTCGGCATTTT 120

************************************************************

Allele 1 GGCGGCAGTCGCCCTTCCCGCCTACCAAGACTACACCGCCCGCGCGCAAGTTTCCGAAGC 180

Allele 11 GGCGGCAGTCGCCCTTCCCGCCTACCAAGACTACACCGCCCGCGCGCAAGTTTCCGAAGC 180

Allele 19 GGCGGCAGTCGCCCTTCCCGCCTACCAAGACTACACCGCCCGCGCGCAAGTTTCCGAAGC 180

Allele 17 GGCGGCAGTCGCCCTTCCCGCCTACCAAGACTACACCGCCCGCGCGCAAGTTTCCGAAGC 180

Allele 20 GGCGGCAGTCGCCCTTCCCGCCTACCAAGACTACACCGCCCGCGCGCAAGTTTCCGAAGC 180

Allele 16 GGCGGCAGTCGCCCTTCCCGCCTACCAAGACTACACCGCCCGCGCGCAAGTTTCCGAAGC 180

Allele 9 GGCGGCAGTCGCCCTTCCCGCCTACCAAGACTACACCGCCCGCGCGCAAGTTTCCGAAGC 180

Allele 18 GGCGGCAGTCGCCCTTCCCGCCTACCAAGACTACACCGCCCGCGCGCAAGTTTCCGAAGC 180

************************************************************

Allele 1 CATCCTTTTGGCCGAAGGTCAAAAATCAGCCGTCACCGAGTATTACCTGAATCACGGCAA 240

Allele 11 CATCCTTTTGGCCGAAGGTCAAAAATCAGCCGTCACCGAGTATTACCTGAATCACGGCAA 240

Allele 19 CATCCTTTTGGCCGAAGGTCAAAAATCAGCCGTCACCGAGTATTACCTGAATCACGGCAT 240

Allele 17 CATCCTTTTGGCCGAAGGTCAAAAATCAGCCGTCACCGAGTATTACCTGAATCACGGCGA 240

Allele 20 CATCCTTTTGGCCGAAGGTCAAAAATCAGCCGTCACCGAGTATTACCTGAATCACGGCAA 240

Allele 16 CATCCTTTTGGCCGAAGGTCAAAAATCAGCCGTCACCGAGTATTACCTGAATCACGGCAA 240

Allele 9 CATCCTTTTGGCCGAAGGTCAAAAATCAGCCGTCACCGAGTATTACCTGAATCACGGCAA 240

Allele 18 CATCCTTTTGGCCGAAGGTCAAAAATCAGCCGTCACCGAGTATTACCTGAATCACGGCAA 240

**********************************************************

Allele 1 ATGGCCGGAAAACAACACTTCTGCCGGCGTGGCATCCCCCCCCTCCGACATCAAAGGCAA 300

Allele 11 ATGGCCGGAAAACAACACTTCTGCCGGCGTGGCATCCCCCCCCTCCGACATCAAAGGCAA 300

Allele 19 ATGGCCGAAAGACAACACTTCTGCCGGCGTGGCATCCCCCCCCTCCGACATCAAAGGCAA 300

Allele 17 ATGGCCGGAAGACAACACTTCTGCCGGCGTGGCATCCTCCCCCACCGACATCAAAGGCAA 300

Allele 20 ATGGCCGGAAAACAACACTTCTGCCGGCGTGGCATCCCCCCCCTCCGACATCAAAGGCAA 300

Allele 16 ATGGCCCAAAGACAACGACTCTGCCGGCGTGGCATCCGCT---TCAAAAATCATAGGCAA 297

Allele 9 ATGGCCGGAAAACAACACTTCTGCCGGCGTGGCATCTTCTTCATC---AATCAAAGGCAA 297

Allele 18 ATGGCCGGAAAACAACACTTCTGCCGGCGTGGCATCCCCCCCCTCCGACATCAAAGGCAA 300

****** ** ***** ***************** * * **** ******

Allele 1 ATATGTTAAAGAGGTTGAAGTTAAAAACGGCGTCGTTACCGCCACAATGCTTTCAAGCGG 360

Allele 11 ATATGTTAAAGAGGTTGAAGTTAAAAACGGCGTCGTTACCGCCACAATGCTTTCAAGCGG 360

Allele 19 ATATGTTAAAGAGGTTGAAGTTAAAAACGGCGTCGTTACCGCCACAATGCTTTCAAGCGG 360

Allele 17 ATATGTTCAAAGCGTTACGGTCGCAAACGGCGTCGTTACCGCCGAAATGGCTTCAACCGG 360

Allele 20 ATATGTTAAAGAGGTTGAAGTTAAAAACGGCGTCGTTACCGCCACAATGAATTCAAGCAA 360

Allele 16 ATATGTTAAGCAAGTTGAAGTCAAAAACGGCGTCGTTACCGCCCAAATGAAATCAGACGG 357

Allele 9 ATATGTTAAGGAAGTTAAAGTCGAAAACGGCGTCGTCACCGCCACAATGAATTCAAGCGG 357

Allele 18 ATATGTTAAAGAGGTTGAAGTTAAAAACGGCGTCGTTACCGCCGAAATGGCTTCAACCGG 360

******* * *** ** ************ ****** **** *** *

Allele 1 CGTAAACAATGAAATCAAAGGCAAAAAACTCTCCCTGTGGGCCAGGCGTGAAAACGGTTC 420

Allele 11 CGTAAACAATGAAATCAAAGGCAAAAAACTCTCCCTGTGGGCCAGGCGTGAAAACGGTTC 420

Allele 19 CGTAAACAATGAAATCAAAGGCAAAAAACTCTCCCTGTGGGCCAGGCGTGAAAACGGTTC 420

Allele 17 CGTAAACAAAGAAATCAAAGGCAAAAAACTCTCCCTGTGGGCCAGGCGTGAAAACGGTTC 420

Allele 20 CGTAAACAAAGAAATCAAAGACAAAAGACTCTCCCTGTGGGGCAGGCGTGAAAACGGTTC 420

Allele 16 CGTAAACAAAGAAATCAAAAACAAAAAACTCTCCCTGTGGGCCAAGCGTGAAAACGGTTC 417

Allele 9 CGTAAACAAAGAAATCCAAGGCAAAAGACTCTCCCTGTGGGCCAAGCGTGAAAACGGTTC 417

Allele 18 CGTAAACAAAGAAATCCAAGGCAAAAGACTCTCCCTGTGGGCCAAGCGTGAAAACGGTTC 420

********* ****** ** ***** ************** ** ***************

Allele 1 GGTAAAATGGTTCTGCGGACAGCCGGTTACGCGCA---CCGACGACGACACCGTT---GC 474

Allele 11 GGTAAAATGGTTCTGCGGACAGCCGGTTACGCGCAACGCCAACGACGACACCGTCACCGC 480

Allele 19 GGTAAAATGGTTCTGCGGACAGCCGGTTACGCGCA---CCGACGACGACACCGTT---GC 474 Allele 17 GGTAAAATGGTTCTGCGGACAGCCGGTTACGCGCA---CCGACGACGACACCGTT---GC 474

Allele 20 GGTAAAATGGTTCTGCGGACAGCCGGTTACGCGCA---CCGACGACGACACCGTT---GC 474

Allele 16 GGTAAAATGGTTCTGCGGACAGCCGGTTACGCGCA---CCGACGACGACACCGTT---GC 471

Allele 9 GGTAAAATGGTTCTGCGGACAGCCGGTTACGCGCA---CCGACGACGACACCGTT---GC 471

Allele 18 GGTAAAATGGTTCTGCGGACAGCCGGTTACGCGCGCCGCCAAAGACGACGACGCC---GT 477

********************************** ** * ****** ** *

Allele 1 CGACG---CCAAAGACGGCAAAGAAATCGACACCAAGCACCTGCCGTCAACCTGCCGCGA 531

Allele 11 CGACGGCACCGGCAACGACGGCAAAATCGACACCAAGCACCTGCCGTCAACCTGCCGCGA 540

Allele 19 CGACG---CCAAAGACGGCAAAGAAATCGACACCAAGCACCTGCCGTCAACCTGCCGCGA 531

Allele 17 CGACG---CCAAAGACGGCAAAGAAATCGACACCAAGCACCTGCCGTCAACCTGCCGCGA 531

Allele 20 CGACG---CCAAAGACGGCAAAGAAATCGACACCAAGCACCTGCCGTCAACCTGCCGCGA 531

Allele 16 CGACG---CCAAAGACGGCAAAGAAATCGACACCAAGCACCTGCCGTCAACCTGCCGCGA 528

Allele 9 CGACG---CCAAAGACGGCAAAGAAATCGACACCAAGCACCTGCCGTCAACCTGCCGCGA 528

Allele 18 CACCG---CCGACGGCAACAACAAAATCGACACCAAGCACCTGCCGTCAACCTGCCGCGA 534

* ** ** * * *************************************

Allele 1 TAAGGCATCTGATGCCAAATGAGGCAAATTAGGCCTTAAATTTTAAATAAATCAAGCGGT 591

Allele 11 TAAGGCATCTGATGCCAAATGAGGCAAATTAGGCCTTAAATTTTAAATAAATCAAGCGGT 600

Allele 19 TAAGGCATCTGATGCCAAATGAGGCAAATTAGGCCTTAAATTTTAAATAAATCAAGCGGT 591

Allele 17 TAAGGCATCTGATGCCAAATGAGGCAAATTAGGCCTTAAATTTTAAATAAATCAAGCGGT 591

Allele 20 TAAGGCATCTGATGCCAAATGAGGCAAATTAGGCCTTAAATTTTAAATAAATCAAGCGGT 591

Allele 16 TAAGGCATCTGATGCCAAATGAGGCAAATTAGGCCTTAAATTTTAAATAAATCAAGCGGT 588

Allele 9 TAAGGCATCTGATGCCAAATGAGGCAAATTAGGCCTTAAATTTTAAATAAATCAAGCGGT 588

Allele 18 CACTTCATCTGC--------------------------------------------CGGT 550

* ****** ****

Allele 1 AAGTGATTTTCCACGGCCGCCCGGATCAACCCGGGCGGCTTGTCTTTTAAGGGTTTGCAA 651

Allele 11 AAGTGATTTTCCACGGCCGCCCGGATCAACCCGGGCGGCTTGTCTTTTAAGGGTTTGCAA 660

Allele 19 AAGTGATTTTCCACGGCCGCCCGGATCAACCCGGGCGGCTTGTCTTTTAAGGGTTTGCAA 651

Allele 17 AAGTGATTTTCCACGGCCGCCCGGATCAACCCGGGCGGCTTGTCTTTTAAGGGTTTGCAA 651

Allele 20 AAGTGATTTTCCACGGCCGCCCGGATCAACCCGGGCGGCTTGTCTTTTAAGGGTTTGCAA 651

Allele 16 AAGTGATTTTCCACGGCCGCCCGGATCAACCCGGGCGGCTTGTCTTTTAAGGGTTTGCAA 648

Allele 9 AAGTGATTTTCCACGGCCGCCCGGATCAACCCGGGCGGCTTGTCTTTTAAGGGTTTGCAA 648

Allele 18 AAGTGATTTTCCACGGCCGCCCGGATCAACCCGGGCGGCTTGTCTTTTAAGGGTTTGCAA 610

************************************************************

**Figure S6:** Alignment of the variant sequences detected in the repeat experiment with *pilE* in *N. gonorrhoeae* MS11. The allele 1 assembly is identical to the reference sequence obtained by Sanger sequencing of the amplicon. Blue text indicates sequence flanking the *pilE* gene (black text). Sequence differences are highlighted in yellow. The grey shading highlights the extent of the sequence identity between the *pilE* sequence and the various silent copies, flanking the variant sequence.
