# Supplementary material for: The Use of High-Throughput DNA Sequencing in the Investigation of Antigenic Variation: Application to Neisseria Species
Source: PLoS One. 2014 Jan 22;9(1):e86704. doi: 10.1371/journal.pone.0086704 (PMC3899283; doi:10.1371/journal.pone.0086704)
Supplement: Figure S7 — Alignment of the variant sequences detected in the first experiment with opaK in N. gonorrhoeae FA1090. The allele 1 assembly is identical to the reference sequence obtained by Sanger sequencing of the amplicon. Blue text indicates sequence flanking the opaK gene (black text). Sequence differences are highlighted in yellow. (DOC) [file pone.0086704.s007.doc]

allele 1 AAATATGTTCAAAGCGTTACGGTCGCAAACGGCGTCGTTACCGCCGAAATGAAACCAAGC 60

allele 4 AAATATGTTCAAAGCGTTACGGTCGCAAACGGCGTCGTTACCGCCGAAATGAAACCAAGC 60

allele 2 AAATATGTTCAAAGCGTTACGGTCGCAAACGGCGTCGTTACCGCCGAAATGAAACCAAGC 60

allele 3 AAATATGTTCAAAGCGTTACGGTCGCAAACGGCGTCGTTACCGCCGAAATGAAACCAAGC 60

************************************************************

allele 1 GGCGTAAACAAAGAAATCAAAGGCAAAAAACTCTCCCTGTGGGCCAAGCGTGAAGACGGT 120

allele 4 GGCGTAAACAAAGAAATCAAAGGCAAAAAACTCTCCCTGTGGGCCAAGCGTGAAGACGGT 120

allele 2 GGCGTAAACAAAGAAATCAAAGGCAAAAAACTCTCCCTGTGGGCCAAGCGTGAAGACGGT 120

allele 3 GGCGTAAACAAAGAAATCAAAGGCAAAAAACTCTCCCTGTGGGCCAAGCGTGAAGACGGT 120

************************************************************

allele 1 TCGGTAAAATGGTTCTGCGGACAGCCGGTTAAGCGCGACGCCGGCGCCAAAGCCGACGAC 180

allele 4 TCGGTAAAATGGTTCTGCGGACAGCCGGTTAAGCGCGACGCCGGCGCCAAAGCCGACGAC 180

allele 2 TCGGTAAAATGGTTCTGCGGACAGCCGGTTAAGCGCGACGCCGGCGCCAAAGCCGACGAC 180

allele 3 TCGGTAAAATGGTTCTGCGGACAGCCGGTTAAGCGCGACGCCGGCGCCAAAGCCGACGAC 180

************************************************************

allele 1 GTCAAAGCCGACGCCGCCAACGCCATCGAAACCAAGCACCTGCCGTCAACCTGCCGCGAT 240

allele 4 GTCAAAGCCGACGCCGCCAACGCCATCGAAACCAAGCACCTGCCGTCAACCTGCCGCGAT 240

allele 2 GTCAAAGCCGACGCCGCCAACGCCATCGAAACCAAGCACCTGCCGTCAACCTGCCGCGAT 240

allele 3 GTCAAAGCCGACGCCGCCAACGCCATCGAAACCAAGCACCTGCCGTCAACCTGCCGCGAT 240

************************************************************

allele 1 GAATCATCTGCCACCTAAGGCAAATTAGGCCTTAAATTTTAAATAAATCAAGCGGTAAGT 300

allele 4 GAATCATCTGCCACCTAAGGCAAATTAGGCCTTAAATTTTAAATAAATCAAGCGGTAAGT 300

allele 2 GAATCATCTGCCACCTAAGGCAAATTAGGCCTTAAATTTTAAATAAATCAAGCGGTAAGT 300

allele 3 GAATCATCTGCCACCTAAGGCAAATTAGGCCTTAAATTTTAAATAAATCAAGCGGTAAGT 300

************************************************************

allele 1 GATTTCCCACGGCCGCCCGGATCAACCCGGGCGGCTTGTCTTTTAAGGGTTTGCAAGGCG 360

allele 4 GATTTCCCACGGCCGCCCGGATCAACCCGGGCGGCTTGTCTTTTAAGGGTTTGCAAGGCG 360

allele 2 GATTTCCCACGGCCGCCCGGATCAACCCGGGCGGCTTGTCTTTTAAGGGTTTGCAAGGCG 360

allele 3 GATTTCCCACGGCCGCCCGGATCAACCCGGGCGGCTTGTCTTTTAAGGGTTTGCAAGGCG 360

************************************************************

allele 1 GGCGGGGTCGTCCGTTCCGGTGGAAATAATATATCGATTGCGCTTCAAGGCCCTGCATGT 420

allele 4 GGCGGGGTCGTCCGTTCCGGTGGAAATAATATATCGATTGCGCTTCAAGGCCCTGCATGT 420

allele 2 GGCGGGGTCGTCCGTTCCGGTGGAAATAATATATCGATTGCGCTTCAAGGCCCTGCATGT 420

allele 3 GGCGGGGTCGTCCGTTCCGGTGGAAATAATATATCGATTGCGCTTCAAGGCCCTGCATGT 420

************************************************************

allele 1 GCCTCATTGCCACCCGTTTAAACACGGTTTTTATCTGACAGGCGCGCAATCCGCCCCCTC 480

allele 4 GCCTCATTGCCACCCGTTTAAACACGGTTTTTATCTGACAGGCGCGCAATCCGCCCCCTC 480

allele 2 GCCTCATTGCCACCCGTTTAAACACGGTTTTTATCTGACAGGCGCGCAATCCGCCCCCTC 480

allele 3 GCCTCATTGCCACCCGTTTAAACACGGTTTTTATCTGACAGGCGCGCAATCCGCCCCCTC 480

************************************************************

allele 1 ATTTGTTAATCCGCCATATTGTATTGAAACACCGCCCGGAACCCGATATAATCCGCCCTT 540

allele 4 ATTTGTTAATCCGCCATATTGTATTGAAACACCGCCCGGAACCCGATATAATCCGCCCTT 540

allele 2 ATTTGTTAATCCGCCATATTGTATTGAAACACCGCCCGGAACCCGATATAATCCGCCCTT 540

allele 3 ATTTGTTAATCCGCCATATTGTATTGAAACACCGCCCGGAACCCGATATAATCCGCCCTT 540

************************************************************

allele 1 CAACATCAGTGAAAATCTTTTTTTAACCGGTTAAACCGAATAAGGAGCCGAAAATGAATC 600

allele 4 CAACATCAGTGAAAATCTTTTTTTAACCGGTTAAACCGAATAAGGAGCCGAAAATGAATC 600

allele 2 CAACATCAGTGAAAATCTTTTTTTAACCGGTTAAACCGAATAAGGAGCCGAAAATGAATC 600

allele 3 CAACATCAGTGAAAATCTTTTTTTAACCGGTTAAACCGAATAAGGAGCCGAAAATGAATC 600

************************************************************

allele 1 CAGCCCGCAAAAAACCTTCTCTTCTCTTCTCTTCTCTTCTCTTCTCTTCTCTTCTCTTCT 660

allele 4 CAGCCCGCAAAAAACCTTCTCTTCTCTTCTCTTCTCTTCTCTTCTCTTCTCTTCTCTTCT 660

allele 2 CAGCCCGCAAAAAACCTTCTCTTCTCTTCTCTTCTCTTCTCTTCTCTTCTCTTCTCTTCT 660

allele 3 CAGCCCGCAAAAAACCTTCTCTTCTCTTCTCTTCTCTTCTCTTCTCTTCTCTTCTCTTCT 660

************************************************************

allele 1 CTTCTCTTCGGCAGCGCAGGCGGCAAGTGAAGGCAATGGCCGCGGCCCGTATGTGCAGGC 720

allele 4 CTTCTCTTCGGCAGCGCAGGCGGCAAGTGAAGGCAATGGCCGCGGCCCGTATGTGCAGGC 720

allele 2 CTTCTCTTCGGCAGCGCAGGCGGCAAGTGAAGGCAATGGCCGCGGCCCGTATGTGCAGGC 720

allele 3 CTTCTCTTCGGCAGCGCAGGCGGCAAGTGAAGGCAATGGCCGCGGCCCGTATGTGCAGGC 720

************************************************************

allele 1 GGATTTAGCCTACGCCGCCGAACGCATTACCCACGATTATCCGGAACCAACCGCTCCAGG 780

allele 4 GGATTTAGCCTACGCCGCCGAACGCATTACCCACGATTATCCGGAACCAACCGCTCCAGG 780

allele 2 GGATTTAGCCTACGCCGCCGAACGCATTACCCACGATTATCCGGAACCAACCGCTCCAGG 780

allele 3 GGATTTAGCCTACGCCGCCGAACGCATTACCCACGATTATCCGGAACCAACCGCTCCAGG 780

************************************************************

allele 1 CAAAAACAAAATAAGCACGGTAAGCGATTATTTCAGAAACATCCGTACGCATTCCATCCA 840

allele 4 CAAAAACAAAATAAGCACGGTAAGCGATTATTTCAGAAACATCCGTACGCATTCCATCCA 840

allele 2 CAAAAACAAAATAAGCACGGTAAGCGATTATTTCAGAAACATCCGTACGCATTCCATCCA 840

allele 3 CAAAAACAAAATAAGCACGGTAAGCGATTATTTCAGAAACATCCGTACGCATTCCATCCA 840

************************************************************

allele 1 CCCCAGGGTGTCGGTCGGCTACGACTTCGGCGGCTGGAGGATAGCGGCAGATTATGCCCG 900

allele 4 CCCCAGGGTGTCGGTCGGCTACGACTTCGGCGGCTGGAGGATAGCGGCAGATTATGCCCG 900

allele 2 CCCCAGGGTGTCGGTCGGCTACGACTTCGGCGGCTGGCGCATCGCCGCGGATTATGCCCG 900

allele 3 CCCCAGGGTGTCGGTCGGCTACGACTTCGGCGGCTGGCGCATCGCCGCGGATTATGCCCG 900

************************************* * ** ** ** ***********

allele 1 TTACAGAAAGTGGAACGACAATAAATATTCCGTCGACATAAAAGAGTTGGAAAACAAGAA 960

allele 4 TTACAGAAAGTGGAACGACAATAAATATTCCGTCGACATAAAAGAGTTGGAAAACAAGAA 960

allele 2 TTACAGGAAATGGCACAACAATAAATATTCCGTGAACATAAAAGAGTTGGAAAGAAAGAA 960

allele 3 TTACAGGAAATGGCACAACAATAAATATTCCGTGAACATAAAAGAGTTGGAAAGAAAGAA 960

****** ** *** ** **************** ****************** *****

allele 1 T---------------------CAG---AATAAGAGAGACCTGAAGACGGAAAATCAGGA 996

allele 4 TAATAAAACTTTTGGCGGCAACCAGCTTAACATAAAATACCAAAAGACGGAACATCAGGA 1020

allele 2 TAATAAAACTTTTGGCGGCAACCAGCTTAACATAAAATACCAAAAGACGGAACATCAGGA 1020

allele 3 TAATAAAACTTTTGGCGGCAACCAGCTTAACATAAAATACCAAAAGACGGAAAATCAGGA 1020

* *** ** * * * *** ********* *******

allele 1 AAACGGCAGCTTCCACGCCGTTTCTTCTCTCGGCTTATCAGCCGTTTACGATTTCAAACT 1056

allele 4 AAACGGCACATTCCACGCCGTTTCTTCTCTCGGCTTGTCCGCCGTTTACGATTTCAAACT 1080

allele 2 AAACGGCACATTCCACGCCGTTTCTTCTCTCGGCTTGTCCGCCGTTTACGATTTCAAACT 1080

allele 3 AAACGGCAGCTTCCACGCCGTTTCTTCTCTCGGCTTATCAGCCGTTTACGATTTCAAACT 1080

******** ************************** ** ********************

allele 1 CAACGACAAATTCAAACCCTATATCGGTGCGCGCGTCGCCTACGGACACGTCAGACACAG 1116

allele 4 CAACGACAAATTCAAACCCTATATCGGTGCGCGCGTCGCCTACGGACACGTCAGACACAG 1140

allele 2 CAACGACAAATTCAAACCCTATATCGGTGCGCGCGTCGCCTACGGACACGTCAGACACAG 1140

allele 3 CAACGACAAATTCAAACCCTATATCGGTGCGCGCGTCGCCTACGGACACGTCAGACACAG 1140

************************************************************

allele 1 CATCGATTCGACTAAAAAAATAACAGGTACTCTTACCGCCTACCCTAGTGATGCTGACGC 1176

allele 4 CATCGATTCGACTAAAAAAATAACAGGTACTCTTACCGCCTACCCTAGTGATGCTGACGC 1200

allele 2 CATCGATTCGACTAAAAAAATAACAGGTACTCTTACCGCCTACCCTAGTGATGCTGACGC 1200

allele 3 CATCGATTCGACTAAAAAAATAACAGGTACTCTTACCGCCTACCCTAGTGATGCTGACGC 1200

************************************************************

allele 1 AGCAGTTACGGTTTATCCTGACGGACATCCGCAAAAAAACACCTATCAAAAAAGCAACAG 1236

allele 4 AGCAGTTACGGTTTATCCTGACGGACATCCGCAAAAAAACACCTATCAAAAAAGCAACAG 1260

allele 2 AGCAGTTACGGTTTATCCTGACGGACATCCGCAAAAAAACACCTATCAAAAAAGCAACAG 1260

allele 3 AGCAGTTACGGTTTATCCTGACGGACATCCGCAAAAAAACACCTATCAAAAAAGCAACAG 1260

************************************************************

allele 1 CAGCCGCCGCTTGGGCTTCGGCGCGATGGCGGGCGTGGGCATAGACGTCGCGCCCGGCCT 1296

allele 4 CAGCCGCCGCTTGGGCTTCGGCGCGATGGCGGGCGTGGGCATAGACGTCGCGCCCGGCCT 1320

allele 2 CAGCCGCCGCTTGGGCTTCGGCGCGATGGCGGGCGTGGGCATAGACGTCGCGCCCGGCCT 1320

allele 3 CAGCCGCCGCTTGGGCTTCGGCGCGATGGCGGGCGTGGGCATAGACGTCGCGCCCGGCCT 1320

************************************************************

allele 1 GACCTTGGACGCCGGCTACCGCTACCACAACTGGGGACGCTTGGAAAACACCCGCTTCAA 1356

allele 4 GACCTTGGACGCCGGCTACCGCTACCACAACTGGGGACGCTTGGAAAACACCCGCTTCAA 1380

allele 2 GACCTTGGACGCCGGCTACCGCTACCACAACTGGGGACGCTTGGAAAACACCCGCTTCAA 1380

allele 3 GACCTTGGACGCCGGCTACCGCTACCACAACTGGGGACGCTTGGAAAACACCCGCTTCAA 1380

************************************************************

allele 1 AACCCACGAAGCCTCATTGGGCATGCGCTACCGCTTCTGATTCCCCGATACCGATGCCGT 1416

allele 4 AACCCACGAAGCCTCATTGGGCATGCGCTACCGCTTCTGATTCCCCGATACCGATGCCGT 1440

allele 2 AACCCACGAAGCCTCATTGGGCATGCGCTACCGCTTCTGATTCCCCGATACCGATGCCGT 1440

allele 3 AACCCACGAAGCCTCATTGGGCATGCGCTACCGCTTCTGATTCCCCGATACCGATGCCGT 1440

************************************************************

allele 1 CTGAACCTTCAGACGGCATTTTTAATCGCCCGCCGTTTACAGGCGCGGGGCGGGCGCGGG 1476

allele 4 CTGAACCTTCAGACGGCATTTTTAATCGCCCGCCGTTTACAGGCGCGGGGCGGGCGCGGG 1500

allele 2 CTGAACCTTCAGACGGCATTTTTAATCGCCCGCCGTTTACAGGCGCGGGGCGGGCGCGGG 1500

allele 3 CTGAACCTTCAGACGGCATTTTTAATCGCCCGCCGTTTACAGGCGCGGGGCGGGCGCGGG 1500

************************************************************

allele 1 GAAATACCCGAACCGTCATTCCCGACAATACCGCAATCTCGAAACCCGTCCGACAACACC 1536

allele 4 GAAATACCCGAACCGTCATTCCCGACAATACCGCAATCTCGAAACCCGTCCGACAACACC 1560

allele 2 GAAATACCCGAACCGTCATTCCCGACAATACCGCAATCTCGAAACCCGTCCGACAACACC 1560

allele 3 GAAATACCCGAACCGTCATTCCCGACAATACCGCAATCTCGAAACCCGTCCGACAACACC 1560

************************************************************

allele 1 GCAATCTCGAAATTCGTCATTCCCGCGCAGGCGGAAATCCGGACCTGTCCGCACGGAAAC 1596

allele 4 GCAATCTCGAAATTCGTCATTCCCGCGCAGGCGGAAATCCGGACCTGTCCGCACGGAAAC 1620

allele 2 GCAATCTCGAAATTCGTCATTCCCGCGCAGGCGGAAATCCGGACCTGTCCGCACGGAAAC 1620

allele 3 GCAATCTCGAAATTCGTCATTCCCGCGCAGGCGGAAATCCGGACCTGTCCGCACGGAAAC 1620

************************************************************

allele 1 TTATCGGATAAAACGGTTGCCCAAACCCCGCGTCCTAGATTCCCACTTCCGTGGGAATGA 1656

allele 4 TTATCGGATAAAACGGTTGCCCAAACCCCGCGTCCTAGATTCCCACTTCCGTGGGAATGA 1680

allele 2 TTATCGGATAAAACGGTTGCCCAAACCCCGCGTCCTAGATTCCCACTTCCGTGGGAATGA 1680

allele 3 TTATCGGATAAAACGGTTGCCCAAACCCCGCGTCCTAGATTCCCACTTCCGTGGGAATGA 1680

************************************************************

allele 1 CGGTTCGGTCTGCCGTTTTCGGACGGCATTTCGACTCAATCCAGCAGTGCGTCCAC 1712

allele 4 CGGTTCGGTCTGCCGTTTTCGGACGGCATTTCGACTCAATCCAGCAGTGCGTCCAC 1736

allele 2 CGGTTCGGTCTGCCGTTTTCGGACGGCATTTCGACTCAATCCAGCAGTGCGTCCAC 1736

allele 3 CGGTTCGGTCTGCCGTTTTCGGACGGCATTTCGACTCAATCCAGCAGTGCGTCCAC 1736

********************************************************

**Figure S7:** Alignment of the variant sequences detected in the first experiment with *opaK* in *N. gonorrhoeae* FA1090. The allele 1 assembly is identical to the reference sequence obtained by Sanger sequencing of the amplicon. Blue text indicates sequence flanking the *opaK* gene (black text). Sequence differences are highlighted in yellow.
