# Supplementary material for: The Use of High-Throughput DNA Sequencing in the Investigation of Antigenic Variation: Application to Neisseria Species
Source: PLoS One. 2014 Jan 22;9(1):e86704. doi: 10.1371/journal.pone.0086704 (PMC3899283; doi:10.1371/journal.pone.0086704)
Supplement: Figure S8 — Alignment of the variant sequences detected in the repeat experiment with opaK in N. gonorrhoeae FA1090. The allele 1 assembly is identical to the reference sequence obtained by Sanger sequencing of the amplicon. Blue text indicates sequence flanking the opaK gene (black text). Sequence differences are highlighted in yellow. (DOC) [file pone.0086704.s008.doc]

allele 1 AAATATGTTCAAAGCGTTACGGTCGCAAACGGCGTCGTTACCGCCGAAATGAAACCAAGC 60

allele 6 AAATATGTTCAAAGCGTTACGGTCGCAAACGGCGTCGTTACCGCCGAAATGAAACCAAGC 60

allele 5 AAATATGTTCAAAGCGTTACGGTCGCAAACGGCGTCGTTACCGCCGAAATGAAACCAAGC 60

************************************************************

allele 1 GGCGTAAACAAAGAAATCAAAGGCAAAAAACTCTCCCTGTGGGCCAAGCGTGAAGACGGT 120

allele 6 GGCGTAAACAAAGAAATCAAAGGCAAAAAACTCTCCCTGTGGGCCAAGCGTGAAGACGGT 120

allele 5 GGCGTAAACAAAGAAATCAAAGGCAAAAAACTCTCCCTGTGGGCCAAGCGTGAAGACGGT 120

************************************************************

allele 1 TCGGTAAAATGGTTCTGCGGACAGCCGGTTAAGCGCGACGCCGGCGCCAAAGCCGACGAC 180

allele 6 TCGGTAAAATGGTTCTGCGGACAGCCGGTTAAGCGCGACGCCGGCGCCAAAGCCGACGAC 180

allele 5 TCGGTAAAATGGTTCTGCGGACAGCCGGTTAAGCGCGACGCCGGCGCCAAAGCCGACGAC 180

************************************************************

allele 1 GTCAAAGCCGACGCCGCCAACGCCATCGAAACCAAGCACCTGCCGTCAACCTGCCGCGAT 240

allele 6 GTCAAAGCCGACGCCGCCAACGCCATCGAAACCAAGCACCTGCCGTCAACCTGCCGCGAT 240

allele 5 GTCAAAGCCGACGCCGCCAACGCCATCGAAACCAAGCACCTGCCGTCAACCTGCCGCGAT 240

************************************************************

allele 1 GAATCATCTGCCACCTAAGGCAAATTAGGCCTTAAATTTTAAATAAATCAAGCGGTAAGT 300

allele 6 GAATCATCTGCCACCTAAGGCAAATTAGGCCTTAAATTTTAAATAAATCAAGCGGTAAGT 300

allele 5 GAATCATCTGCCACCTAAGGCAAATTAGGCCTTAAATTTTAAATAAATCAAGCGGTAAGT 300

************************************************************

allele 1 GATTTCCCACGGCCGCCCGGATCAACCCGGGCGGCTTGTCTTTTAAGGGTTTGCAAGGCG 360

allele 6 GATTTCCCACGGCCGCCCGGATCAACCCGGGCGGCTTGTCTTTTAAGGGTTTGCAAGGCG 360

allele 5 GATTTCCCACGGCCGCCCGGATCAACCCGGGCGGCTTGTCTTTTAAGGGTTTGCAAGGCG 360

************************************************************

allele 1 GGCGGGGTCGTCCGTTCCGGTGGAAATAATATATCGATTGCGCTTCAAGGCCCTGCATGT 420

allele 6 GGCGGGGTCGTCCGTTCCGGTGGAAATAATATATCGATTGCGCTTCAAGGCCCTGCATGT 420

allele 5 GGCGGGGTCGTCCGTTCCGGTGGAAATAATATATCGATTGCGCTTCAAGGCCCTGCATGT 420

************************************************************

allele 1 GCCTCATTGCCACCCGTTTAAACACGGTTTTTATCTGACAGGCGCGCAATCCGCCCCCTC 480

allele 6 GCCTCATTGCCACCCGTTTAAACACGGTTTTTATCTGACAGGCGCGCAATCCGCCCCCTC 480

allele 5 GCCTCATTGCCACCCGTTTAAACACGGTTTTTATCTGACAGGCGCGCAATCCGCCCCCTC 480

************************************************************

allele 1 ATTTGTTAATCCGCCATATTGTATTGAAACACCGCCCGGAACCCGATATAATCCGCCCTT 540

allele 6 ATTTGTTAATCCGCCATATTGTATTGAAACACCGCCCGGAACCCGATATAATCCGCCCTT 540

allele 5 ATTTGTTAATCCGCCATATTGTATTGAAACACCGCCCGGAACCCGATATAATCCGCCCTT 540

************************************************************

allele 1 CAACATCAGTGAAAATCTTTTTTTAACCGGTTAAACCGAATAAGGAGCCGAAAATGAATC 600

allele 6 CAACATCAGTGAAAATCTTTTTTTAACCGGTTAAACCGAATAAGGAGCCGAAAATGAATC 600

allele 5 CAACATCAGTGAAAATCTTTTTTTAACCGGTTAAACCGAATAAGGAGCCGAAAATGAATC 600

************************************************************

allele 1 CAGCCCGCAAAAAACCTTCTCTTCTCTTCTCTTCTCTTCTCTTCTCTTCTCTTCTCTTCT 660

allele 6 CAGCCCGCAAAAAACCTTCTCTTCTCTTCTCTTCTCTTCTCTTCTCTTCTCTTCTCTTCT 660

allele 5 CAGCCCGCAAAAAACCTTCTCTTCTCTTCTCTTCTCTTCTCTTCTCTTCTCTTCTCTTCT 660

************************************************************

allele 1 CTTCTCTTCGGCAGCGCAGGCGGCAAGTGAAGGCAATGGCCGCGGCCCGTATGTGCAGGC 720

allele 6 CTT-----CGGCAGCGCAGGCGGCAAGTGAAGGCAATGGCCGCGGCCCGTATGTGCAGGC 715

allele 5 CTT-----CGGCAGCGCAGGCGGCAAGTGAAGGCAATGGCCGCGGCCCGTATGTGCAGGC 715

*** ****************************************************

allele 1 GGATTTAGCCTACGCCGCCGAACGCATTACCCACGATTATCCGGAACCAACCGCTCCAGG 780

allele 6 GGATTTAGCCTACGCCGCCGAACGCATTACCCACGATTATCCGGAACCAACCGCTCCAGG 775

allele 5 GGATTTAGCCTACGCCGCCGAACGCATTACCCACGATTATCCGGAACCAACCGCTCCAGG 775

************************************************************

allele 1 CAAAAACAAAATAAGCACGGTAAGCGATTATTTCAGAAACATCCGTACGCATTCCATCCA 840

allele 6 CAAAAACAAAATAAGCACGGTAAGCGATTATTTCAGAAACATCCGTACGCATTCCATCCA 835

allele 5 CAAAAACAAAATAAGCACGGTAAGCGATTATTTCAGAAACATCCGTACGCATTCCATCCA 835

************************************************************

allele 1 CCCCAGGGTGTCGGTCGGCTACGACTTCGGCGGCTGGAGGATAGCGGCAGATTATGCCCG 900

allele 6 CCCCAGGGTGTCGGTCGGCTACGACTTCGGCGGCTGGAGGATAGCGGCAGATTATGCCCG 895

allele 5 CCCCAGGGTGTCGGTCGGCTACGACTTCGGCGGCTGGCGCATCGCCGCGGATTATGCCCG 895

************************************* * ** ** ** ***********

allele 1 TTACAGAAAGTGGAACGACAATAAATATTCCGTCGACATAAAAGAGTTGGAAAACAAGAA 960

allele 6 TTACAGAAAGTGGAACGACAATAAATATTCCGTCGACATAAAAGAGTTGGAAAACAAGAA 955

allele 5 TTACAGGAAATGGCACAACAATAAATATTCCGTGAACATAAAAGAGTTGGAAAGAAAGAA 955

****** ** *** ** **************** ****************** *****

allele 1 T---------------------CAG---AATAAGAGAGACCTGAAGACGGAAAATCAGGA 996

allele 6 T---------------------CAG---AATAAGAGAGACCTGAAGACGGAACATCAGGA 991

allele 5 TAATAAAACTTTTGGCGGCAACCAGCTTAACATAAAATACCAAAAGACGGAACATCAGGA 1015

* *** ** * * * *** ********* *******

allele 1 AAACGGCAGCTTCCACGCCGTTTCTTCTCTCGGCTTATCAGCCGTTTACGATTTCAAACT 1056

allele 6 AAACGGCACATTCCACGCCGTTTCTTCTCTCGGCTTGTCCGCCGTTTACGATTTCAAACT 1051

allele 5 AAACGGCACATTCCACGCCGTTTCTTCTCTCGGCTTGTCCGCCGTTTACGATTTCAAACT 1075

******** ************************** ** ********************

allele 1 CAACGACAAATTCAAACCCTATATCGGTGCGCGCGTCGCCTACGGACACGTCAGACACAG 1116

allele 6 CAACGACAAATTCAAACCCTATATCGGTGCGCGCGTCGCCTACGGACACGTCAGACACAG 1111

allele 5 CAACGACAAATTCAAACCCTATATCGGTGCGCGCGTCGCCTACGGACACGTCAGACACAG 1135

************************************************************

allele 1 CATCGATTCGACTAAAAAAATAACAGGTACTCTTACCGCCTACCCTAGTGATGCTGACGC 1176

allele 6 CATCGATTCGACTAAAAAAATAACAGGTACTCTTACCGCCTACCCTAGTGATGCTGACGC 1171

allele 5 CATCGATTCGACTAAAAAAATAACAGGTACTCTTACCGCCTACCCTAGTGATGCTGACGC 1195

************************************************************

allele 1 AGCAGTTACGGTTTATCCTGACGGACATCCGCAAAAAAACACCTATCAAAAAAGCAACAG 1236

allele 6 AGCAGTTACGGTTTATCCTGACGGACATCCGCAAAAAAACACCTATCAAAAAAGCAACAG 1231

allele 5 AGCAGTTACGGTTTATCCTGACGGACATCCGCAAAAAAACACCTATCAAAAAAGCAACAG 1255

************************************************************

allele 1 CAGCCGCCGCTTGGGCTTCGGCGCGATGGCGGGCGTGGGCATAGACGTCGCGCCCGGCCT 1296

allele 6 CAGCCGCCGCTTGGGCTTCGGCGCGATGGCGGGCGTGGGCATAGACGTCGCGCCCGGCCT 1291

allele 5 CAGCCGCCGCTTGGGCTTCGGCGCGATGGCGGGCGTGGGCATAGACGTCGCGCCCGGCCT 1315

************************************************************

allele 1 GACCTTGGACGCCGGCTACCGCTACCACAACTGGGGACGCTTGGAAAACACCCGCTTCAA 1356

allele 6 GACCTTGGACGCCGGCTACCGCTACCACAACTGGGGACGCTTGGAAAACACCCGCTTCAA 1351

allele 5 GACCTTGGACGCCGGCTACCGCTACCACAACTGGGGACGCTTGGAAAACACCCGCTTCAA 1375

************************************************************

allele 1 AACCCACGAAGCCTCATTGGGCATGCGCTACCGCTTCTGATTCCCCGATACCGATGCCGT 1416

allele 6 AACCCACGAAGCCTCATTGGGCATGCGCTACCGCTTCTGATTCCCCGATACCGATGCCGT 1411

allele 5 AACCCACGAAGCCTCATTGGGCATGCGCTACCGCTTCTGATTCCCCGATACCGATGCCGT 1435

************************************************************

allele 1 CTGAACCTTCAGACGGCATTTTTAATCGCCCGCCGTTTACAGGCGCGGGGCGGGCGCGGG 1476

allele 6 CTGAACCTTCAGACGGCATTTTTAATCGCCCGCCGTTTACAGGCGCGGGGCGGGCGCGGG 1471

allele 5 CTGAACCTTCAGACGGCATTTTTAATCGCCCGCCGTTTACAGGCGCGGGGCGGGCGCGGG 1495

************************************************************

allele 1 GAAATACCCGAACCGTCATTCCCGACAATACCGCAATCTCGAAACCCGTCCGACAACACC 1536

allele 6 GAAATACCCGAACCGTCATTCCCGACAATACCGCAATCTCGAAACCCGTCCGACAACACC 1531

allele 5 GAAATACCCGAACCGTCATTCCCGACAATACCGCAATCTCGAAACCCGTCCGACAACACC 1555

************************************************************

allele 1 GCAATCTCGAAATTCGTCATTCCCGCGCAGGCGGAAATCCGGACCTGTCCGCACGGAAAC 1596

allele 6 GCAATCTCGAAATTCGTCATTCCCGCGCAGGCGGAAATCCGGACCTGTCCGCACGGAAAC 1591

allele 5 GCAATCTCGAAATTCGTCATTCCCGCGCAGGCGGAAATCCGGACCTGTCCGCACGGAAAC 1615

************************************************************

allele 1 TTATCGGATAAAACGGTTGCCCAAACCCCGCGTCCTAGATTCCCACTTCCGTGGGAATGA 1656

allele 6 TTATCGGATAAAACGGTTGCCCAAACCCCGCGTCCTAGATTCCCACTTCCGTGGGAATGA 1651

allele 5 TTATCGGATAAAACGGTTGCCCAAACCCCGCGTCCTAGATTCCCACTTCCGTGGGAATGA 1675

************************************************************

allele 1 CGGTTCGGTCTGCCGTTTTCGGACGGCATTTCGACTCAATCCAGCAGTGCGTCCAC 1712

allele 6 CGGTTCGGTCTGCCGTTTTCGGACGGCATTTCGACTCAATCCAGCAGTGCGTCCAC 1707

allele 5 CGGTTCGGTCTGCCGTTTTCGGACGGCATTTCGACTCAATCCAGCAGTGCGTCCAC 1731

********************************************************

**Figure S8:** Alignment of the variant sequences detected in the repeat experiment with *opaK* in *N. gonorrhoeae* FA1090. The allele 1 assembly is identical to the reference sequence obtained by Sanger sequencing of the amplicon. Blue text indicates sequence flanking the *opaK* gene (black text). Sequence differences are highlighted in yellow.
