# Supplementary material for: The Use of High-Throughput DNA Sequencing in the Investigation of Antigenic Variation: Application to Neisseria Species
Source: PLoS One. 2014 Jan 22;9(1):e86704. doi: 10.1371/journal.pone.0086704 (PMC3899283; doi:10.1371/journal.pone.0086704)
Supplement: Table S1 — Oligonucleotide primers used to amplify the genes of interest. (DOC) [file pone.0086704.s009.doc]

**Table S1: Oligonucleotide primers used to amplify the genes of interest.**

| ***N. gonorrhoeae* FA1090** | | |
| --- | --- | --- |
| Target gene | Oligonucleotide number | Sequence |
| *pilE* | DAP2008  DAP2005 | CCCTATTCTAACGCGTAAATTC  CATTTTCGGCTCCTTATTCGG |
| *opaJ*  (NGO1922) | DAP2006  DAP2007 | ACCATATCAACGCAATTGGGAA  CGGTATTATACAAGACCTGTCGAAG |
| *opaK*  (NGO2132) | DAP2066  DAP2067 | GGCAAATATGTTCAAAGCGT  CGTTTGTGGACGCACTGCTG |
| ***N. gonorrhoeae* MS11** | | |
| *pilE* | DAP2113  DAP2114 | TCCCCTTTCAATTAGGAGT  TCGATATATTATTTCCACC |
| ***N. meningitidis* NMB** | | |
| *pilE* (class II) | DAP2092  DAP2094 | GTCACAACTGACAAGAAACG  CTTGTTTTCAGGGCGTGC |
| *pilE* in CKNM397  (MC58 class I) | DAP2095  DAP2096 | AATTACTCAAACCACGCC  GACTTTTGGAACACATCCG |
| ***N. meningitidis* FAM18** | | |
| *pilE* (class II) | DAP2092  DAP2093 | GTCACAACTGACAAGAAACG  CCGAATCCCAACGGTTCG |
