# Supplementary material for: The Use of High-Throughput DNA Sequencing in the Investigation of Antigenic Variation: Application to Neisseria Species
Source: PLoS One. 2014 Jan 22;9(1):e86704. doi: 10.1371/journal.pone.0086704 (PMC3899283; doi:10.1371/journal.pone.0086704)
Supplement: Table S2 — Sequence quality data. (DOC) [file pone.0086704.s010.doc]

| Strain | Gene | Experiment | Condition | k used | Raw pair count | Read length | Q10 average length^a^ | Q20 average length^b^ | Q30 average length^c^ | Pairs longer than k after clipping | Single reads longer than k after clipping | Average read length of read longer than k after clipping |
| --- | --- | --- | --- | --- | --- | --- | --- | --- | --- | --- | --- | --- |
| FA1090 | *pilE* | 1 | RecA^-^ | 30 | 3570393 | 101 | 86.7 | 73.9 | 52.1 | 2871938 | 391521 | 76.8 |
| FA1090 | *pilE* | 1 | RecA^+^ | 30 | 15423940 | 101 | 86.5 | 73.6 | 51.6 | 12414274 | 1674442 | 76.8 |
| FA1090 | *pilE* | 2 | RecA^-^ | 50 | 8231485 | 101 | 62.7 | 49.9 | 34.3 | 5614198 | 411232 | 75.5 |
| FA1090 | *pilE* | 2 | RecA^+^ | 50 | 5399387 | 101 | 61.9 | 48.8 | 33.7 | 3593646 | 314371 | 75.3 |
| MS11 | *pilE* | 1 | RecA^-^ | 30 | 4841714 | 76 | 51.7 | 45.7 | 34.5 | 3749972 | 420492 | 49.0 |
| MS11 | *pilE* | 1 | RecA^+^ | 30 | 3888814 | 76 | 52.4 | 46.4 | 35.0 | 3068367 | 343934 | 49.6 |
| MS11 | *pilE* | 2 | RecA^-^ | 30 | 7684482 | 79 | 44.4 | 28.7 | 11.0 | 4139825 | 1436614 | 48.2 |
| MS11 | *pilE* | 2 | RecA^+^ | 30 | 7691690 | 79 | 45.2 | 29.2 | 11.2 | 4303951 | 1445555 | 48.6 |
| FAM18 | *pilE* | 1 | RecA^+^ | 30 | 22239209 | 75 | 52.8 | 46.0 | 35.0 | 13278485 | 4733138 | 51.6 |
| FAM18 | *pilE* | 2 | RecA^+^ | 30 | 12089013 | 101 | 86.4 | 70.9 | 46.4 | 9679915 | 1104181 | 75.5 |
| NMB | *pilE* | 1 | RecA^+^ | 30 | 11509861 | 101 | 82.9 | 67.1 | 43.1 | 8704569 | 1270132 | 73.2 |
| CKNM397 | *pilE* | 1 | RecA^+^ | 50 | 7237777 | 101 | 66.0 | 53.2 | 36.8 | 5232278 | 325200 | 77.5 |
| CKNM397 | *pilE* | 2 | RecA^+^ | 50 | 5562633 | 101 | 65.0 | 51.8 | 35.5 | 3939626 | 254958 | 76.9 |
| FA1090 | *opaK* | 1 | RecA^-^ | 50 | 12125942 | 101 | 68.0 | 55.2 | 39.7 | 6091224 | 877613 | 74.3 |
| FA1090 | *opaK* | 1 | RecA^+^ | 50 | 14154924 | 101 | 69.4 | 56.4 | 40.6 | 7572874 | 1052849 | 74.4 |
| FA1090 | *opaK* | 2 | RecA^-^ | 50 | 5369370 | 101 | 59.1 | 46.0 | 31.2 | 3381187 | 280238 | 72.7 |
| FA1090 | *opaK* | 2 | RecA^+^ | 50 | 6921549 | 101 | 59.2 | 46.5 | 31.8 | 4380718 | 354293 | 73.1 |

^a^ the average length before the first base with quality less than 10 (10% chance of miscalled base) is encountered.

^b^ the average length before the first base with quality less than 20 (1% chance of miscalled base) is encountered.

^c^ the average length before the first base with quality less than 30 (0.1% chance of miscalled base) is encountered.
