# Supplementary material for: The Use of High-Throughput DNA Sequencing in the Investigation of Antigenic Variation: Application to Neisseria Species
Source: PLoS One. 2014 Jan 22;9(1):e86704. doi: 10.1371/journal.pone.0086704 (PMC3899283; doi:10.1371/journal.pone.0086704)
Supplement: Text File S1 — Assembly of sequence reads. (DOC) [file pone.0086704.s011.doc]

# SUPPLEMENTARY TEXT: ASSEMBLY SOFTWARE

**Initial read cleanup**

Reads were clipped where the quality fell below 20 (1% chance of miscall), and to remove Illumina adaptor sequences.

**Assembly stage**

Each sequence was assembled starting from a seed k-mer. The seed for each sequence in turn was chosen as the most common k-mer that had not been a part of previously assembled sequences. To assemble each sequence, the seed k-mer was progressively extended at either end for so long as the k-mer pair counts clearly indicated a best extension. In total 500 sequences were produced per sample or per RecA^-^/RecA^+^ pair of samples.

Each possible extension (A, C, G or T) at either end was considered. An extension was given a score based on the number of times the new k-mer that would result from the extension was observed paired with k-mers already in the assembly. An observed pairing with a k-mer already in the assembly was weighted in inverse proportion to the number of times the k-mer in the assembly occurred in the reads, so that evidence from the variant part of an assembly was not overwhelmed by evidence from k-mers present in most sequences. Also, the score was reduced if the kmer was observed pairing with the reverse complement of k-mers already in the assembly.

The variance of the score under the assumption that counts were Poisson distributed was also calculated. Extension halted when, at both ends of the sequence, there was no extension that was better than the other options by at least three standard deviations. As what was of interest was variants within sequences, low depth regions at the ends of the sequence were then clipped.

K-mer length k=30 for was used for 36-cycle chemistry and length k=50 for 72-cycle chemistry.

**Selection stage**

From the 500 assembled sequences, 50 sequences of greatest relevance were chosen in turn. Each sequence was chosen for having as great as possible a total count of k-mers that were not present in previously chosen sequences. That is, we added up for each k-mer in the sequence the number of times it occurred in the read data. K-mers that were merely a SNP away from a previously seen k-mer were given a weight of 0.001 times that of other k-mers, so as not to discard them but to give priority to more relevant sequences first.

**Manual validation stage**

Heat-maps of the 50 selected sequences were produced showing the number of occurrences of each pair of k-mers. In these both the x and y axis was position in the assembled sequence of k-mers, and the brightness of each point was the number of times the corresponding k-mer pair was observed in the read data. This allowed for manual checking for mis-assemblies. Mis-assembly could be seen as the unexpected absence of pairs of k-mers between two parts of an assembled sequence. Similar heat maps were produced for the case where one of the k-mers was reversed. Excessive depth of such k-mer pairs could also indicate mis-assembly.

If it happens that most of the 50 sequences turn out to be of interest after manual examination, it is possible to continue generating sequences beyond the initial 500, and sequences of greatest relevance beyond the initial 50.

**Discussion**

We have attempted to create a procedure that produces as complete a set of assemblies as the read data allows, and that produces sequence assemblies that are correct. We expect the set of assemblies to be complete because it has been designed to ensure that there are sequences representing an explanation of the highest frequency k-mers observed in the reads. The initial assembly process ensures there are sequences containing all of the high frequency k-mers in the data. If a mis-assembly of some sequence occurs, a k-mer that should have been in that assembly will not have been included, and will be used as a seed in a later assembly. Then in the selection stage, sequences are chosen so as to include k-mers with as high coverage as possible. We expect the assemblies to be correct because the k-mer pair heatmaps allows manual diagnosis of mis-assemblies.

The assembly procedure is a variant on k-mer based assembly [1]. We also note the recently published SPAdes assembler [2] is also based on k-mer pairs. SPAdes uses k-mer pairs that have as close as possible to a fixed spacing in the sequence to be assembled. Here we have used k-mers that are merely in order in the sequence. For reads of length *n*, containing *m*=*n*-*k*+1 k-mers, SPAdes produces *m* k-mer pairs per read pair, whereas the present algorithm produces 2*m*^2^-*m* k-mer pairs per read pair. This larger number of k-mer pairs per read pair is viable here because the sequences being assembled are short and so the number of unique k-mer pairs is manageably small.

**References**

[1] P. A. Pevzner, H. Tang and M. S. Waterman (2001), An Eulerian path approach to DNA fragment assembly. Proc. Natl. Acad. Sci. USA, 98:9748-9753.

[2] Bankevich A, Nurk S, Antipov D, Gurevich AA, Dvorkin M, Kulikov AS, Lesin VM, Nikolenko SI, Pham S, Prjibelski AD, Pyshkin AV, Sirotkin AV, Vyahhi N, Tesler G, Alekseyev MA, Pevzner PA. SPAdes: a new genome assembly algorithm and its applications to single-cell sequencing. J Comput Biol. 2012 May;19(5):455-77. Epub 2012 Apr 16.
